# Changes over time in creatinine clearance and comparison of emergent adverse events for HIV-positive adults receiving standard doses (300 mg/day) of lamivudine-containing antiretroviral therapy with baseline creatinine clearance of 30–49 vs ≥50 mL/min

**DOI:** 10.1371/journal.pone.0225199

**Published:** 2019-11-14

**Authors:** Lisa L. Ross, A. Sarah Walker, Yu Lou, Allan R. Tenorio, Diana M. Gibb, Julia Double, Charles Gilks, Cynthia C. McCoig, Paula Munderi, Godfrey Musoro, Cissy M. Kityo, Heiner Grosskurth, James Hakim, Peter N. Mugyenyi, Amy Cutrell, Teodora Perger, Mark S. Shaefer

**Affiliations:** 1 Medical Affairs, ViiV Healthcare, Research Triangle Park, NC, United States of America; 2 Medical Research Council Clinical Trials Unit, University College, London, United Kingdom; 3 Statistics, PAREXEL International, Durham, NC, United States of America; 4 Physicians Group, ViiV Healthcare, Research Triangle Park, NC, United States of America; 5 Safety and Medical Governance, GlaxoSmithKline, Stockley Park, United Kingdom; 6 School of Population Health, University of Queensland, Brisbane, Australia; 7 Physicians Group, ViiV Healthcare, Tres Cantos, Spain; 8 HIV Care Research Programme, MRC/UVRI Uganda Research Unit on AIDS, Entebbe, Uganda; 9 Department of Medicine, University of Zimbabwe, Harare, Zimbabwe; 10 HIV Clinical Trials Unit, Joint Clinical Research Centre, Kampala, Uganda; 11 Healthcare Statistics, ViiV Healthcare, Research Triangle Park, NC, United States of America; 12 Safety and Pharmacovigilance, ViiV Healthcare, London, United Kingdom; Imperial College London, UNITED KINGDOM

## Abstract

A retrospective analysis of the randomized controlled DART (Development of AntiRetroviral Therapy in Africa; ISRCTN13968779) trial in HIV-1-positive adults initiating antiretroviral therapy with co-formulated zidovudine/lamivudine plus either tenofovir, abacavir, or nevirapine was conducted to evaluate the safety of initiating standard lamivudine dosing in patients with impaired creatinine clearance (CLcr). Safety data collected through 96 weeks were analyzed after stratification by baseline CLcr (estimated using Cockcroft-Gault) of 30–49 mL/min (n = 168) versus ≥50 mL/min (n = 3,132) and treatment regimen. The Grade 3–4 adverse events (AEs) and serious AEs (for hematological, hepatic and gastrointestinal events), maximal toxicities for liver enzymes, serum creatinine and bilirubin and maximum treatment-emergent hematology toxicities were comparable for groups with baseline CLcr 30–49 versus CLcr≥50 mL/min. No new risks or trends were identified from this dataset. Substantial and similar increases in the mean creatinine clearance (>25 mL/min) were observed from baseline though Week 96 among participants who entered the trial with CLcr 30–49 mL/min, while no increase or smaller median changes in creatinine clearance (<7 mL/min) were observed for participants who entered the trial with CLcr ≥50 mL/min. Substantial increases (> 150 cells/ mm^3^) in mean CD4+ cells counts from baseline to Week 96 were also observed for participants who entered the trial with CLcr 30–49 mL/min and those with baseline CLcr ≥50 mL/min. Though these results are descriptive, they suggest that HIV-positive patients with CLcr of 30–49 mL/min would have similar AE risks in comparison to patients with CLcr ≥50 mL/min when initiating antiretroviral therapy delivering doses of 300 mg of lamivudine daily through 96 weeks of treatment. Overall improvements in CLcr were observed for patients with baseline CLcr 30–49 mL/min.

## Introduction

For HIV-1-positive patients with impaired renal function whose creatinine clearance (CLcr) is between 30–49 mL/min, the current recommended dose of lamivudine (3TC) is 150 mg once-daily (i.e., a 50% reduced daily dose). This dosing recommendation is based on pharmacokinetic data from the NUCA1004 study in HIV-1-positive patients, in which an approximately 2-fold increase in 3TC exposure in plasma was observed for patients with CLcr 30–49 mL/min following oral administration of a 300 mg 3TC tablet; however long-term safety data were not available in that study [1], and no large-scale trials have investigated standard-dose 3TC in patients with impaired renal function. To better understand the impact on longer term safety of initiating a standard 3TC dose or of a 3TC-containing fixed dose combination (FDC) in HIV-positive patients with CLcr 30-49mL/min, a retrospective analysis was performed using data from the Development of AntiRetroviral Therapy in Africa (DART) clinical trial. In DART, HIV-positive patients received combination antiretroviral therapy (cART) containing an FDC of 3TC/zidovudine (ZDV) plus a third antiretroviral drug (ARV) [2, 3]. The incidence of safety events, including adverse events (AEs) and serious adverse events (SAEs) and CLcr dynamics from baseline to Week 96 were compared between trial participants with baseline CLcr 30–49 mL/min and baseline CLcr ≥50 mL/min.

## Materials and methods

### Trial design

DART (ISRCTN13968779) was an open-label, non-inferiority, randomized trial comparing the use of clinically driven monitoring (CDM; n = 1,660) to routine laboratory and clinical monitoring (LCM; n = 1,656) in antiretroviral-naïve, symptomatic HIV-positive adults who were initiating cART with CD4 <200 cells/mm^3^ [DART, 2010]. The trial was conducted at three centers in Uganda and one in Zimbabwe. Each participant received 3TC/ZDV twice-daily (BID) as an FDC (150 mg 3TC and 300 mg ZDV) in combination with a third drug, which was either once-daily (QD) tenofovir (TDF; 245 mg) for 2,469 trial participants, nevirapine (NVP; 200 mg QD for 14 days followed by 200 mg BID thereafter) for 547 trial participants or abacavir (ABC; 300 mg BID) for 300 trial participants [2, 3].

The primary endpoint was progression to a new World Health Organization (WHO)-defined HIV Stage 4 disease event or death. In both groups, trial participants had hematology, liver function tests and CD4 cell counts performed, but these results were not routinely disclosed to clinicians managing participants in the CDM group unless they were a Grade 4 abnormality, or the results were requested by the clinician for a clinical reason (CD4 cell counts were never returned for CDM participants). Additional detail describing the trial design and sub-studies are available and in the protocol (S1 File) [3]. Trial enrollment occurred between January 2003 and October 2004. A planned sub-study within DART examined the impact of structured treatment interruptions (STI) for a subgroup of participants with CD4 cell counts ≥300 cell/mm^3^ after 48 or 72 weeks on continuous ART who were factorially randomized to STI (12 weeks on ART, followed by 12 weeks off ART) versus remaining on continuous ART. The sub-study was terminated in March 2006 due to inferior performance of the STI group compared to continuous ART [2]. Data from trial visits after the start of the first STI for 225/3316 of these sub-study participants were excluded from this post hoc analysis.

Participants gave written informed consent both for screening and, if eligible, enrollment. The trial was approved by research ethics committees from the participating study sites in Uganda (Joint Clinical Research Centre Research Ethics Committee and the Uganda Virus Research Institute Science and Ethics Committee) and Zimbabwe (Joint Research Ethics Committee for the University of Zimbabwe, College of Health Sciences, the Parirenyatwa Group of Hospitals, and the Medical Research Council of Zimbabwe) as well as by ethics committees in the United Kingdom (Liverpool School of Tropical Medicine and Imperial College).

### Laboratory and clinical investigations

Previously collected on treatment data were analyzed through 96 weeks, including Grade 3–4 AEs and SAEs (as per the trial protocol, events solely associated with HIV disease, including deaths related to HIV alone, were exempted from reporting as SAEs and AE events were classified using a coding system developed by the DART investigators that was based on diseases likely to occur in an African setting). Trial participants underwent clinical examinations at baseline, weeks 4 and 12 and every 12 weeks thereafter. Full blood count and selected biochemistry test results (including creatinine, urea, bilirubin, alanine amino transferase and aspartate amino transferase) and CD4 cell counts were collected on the same schedule. Local laboratories determined serum creatinine and no specific calibration was undertaken across centers. Plasma HIV-1 RNA, and resistance testing were not performed prospectively. Treatment switches were guided by immunological (CD4+ cell counts; LCM group only) and clinical parameters only. Additional details, including full inclusion and exclusion criteria for the original trial, are available as supplementary information (S1 File).

### Statistical analysis

Although the randomization schedule assigned the participants to LDM vs CDM, for the purpose of this retrospective analysis that randomization was combined, and treatment groups were summarized according to the third drug in the regimen and by baseline CLcr category. All available clinical laboratory data for trial participants was summarized for Week 0, 24, 48 and 96 and grouped by ART regimen and by baseline CLcr categories for subsequent analysis. The DAIDS grading scale was used for laboratory grading and the National Kidney Foundation (NKF) grading scale was used for the creatinine clearance grading [3, 4].

The incidences of safety events were compared between participants with baseline CLcr ≥50 mL/min, 30–49 mL/min and <30 mL/min. In participants with available CLcr data at Week 24, the incidence of safety events that occurred in the first 24 weeks were also compared between those participants whose baseline CLcr was 30–49 mL/min and remained <50 mL/min at Week 24 versus those whose baseline CLcr was 30–49 mL/min and increased to ≥50 mL/min by Week 24, and those from whom the baseline and Week 24 CLcr results were ≥50 mL/min. The Cockcroft-Gault formula (CGF) was used to calculate CLcr [5]; and CLcr change from baseline, i.e., changes over time were evaluated. Since TDF can impact renal function and TDF plasma levels are affected by renal function, for some analyses, the TDF-treated participants were analyzed separately. The number of Grade 3–4 AEs and SAEs observed was summarized by ART treatment regimen and baseline CGF CLcr values without adjustment for body surface area (BSA). For trial participants who took part in an STI, any data collected after the start of their first STI (if the STI occurred within the first 96 weeks of treatment) were excluded from the analysis.

## Results

### DART trial population

Baseline ART regimens by baseline CLcr for the HIV-positive, ART-naive DART trial participants are summarized in Table 1. There were 168 participants enrolled into DART with CLcr 30–49 mL/min; 46 participants received either ABC or NVP as the third ARV drug.

**Table 1:** Summary of baseline ART regimens by baseline CLcr (mL/min) in the DART Trial.

Regimen	CLcr ≥50 mL/min (n = 3132)	CLcr 30–49 mL/min (n = 168)	CLcr <30 mL/min (n = 13)	Total (n = 3313)^a^
	n (%)			
3TC/ZDV + TDF	2335 (75)	122 (73)	9 (69)	2469 (74)
3TC/ZDV + ABC	272 (9)	27 (16)	1 (8)	300 (9)
3TC/ZDV + NVP	525 (17)	19 (11)	3 (23)	547 (6)

ABC, abacavir; ART, antiretroviral therapy; CLcr, creatinine clearance; 3TC, lamivudine; NVP, nevirapine; TDF, tenofovir; ZDV, zidovudine.

^a^ There were 3 participants with missing baseline CLcr and were excluded from the total N.

The demographics of the DART trial participants were summarized by treatment regimen and by baseline CLcr (Table 2). All trial participants were from Uganda or Zimbabwe. Overall, demographic characteristics were similar across the CLcr-defined groups. The majority were women (65%); women who had previously received only short-course ART to prevent mother-to-child transmission were considered ART-naïve for purposes of enrollment. The median age of the trial participants was 36.8 years; median enrollment CD4 cell count was 86 cells/mm^3^ and 79% had advanced HIV disease (WHO Stage 3/4). The overall median baseline CGF creatinine clearance for the 3,316 DART participants was 83 mL/min.

**Table 2:** DART trial demographic characteristics summarized by treatment regimen and by baseline CLcr (mL/min).

Baseline^a^	TDF-Containing ART		ABC or NVP-Containing ART		Any ART			Overall (N = 3313)
	CLcr ≥50 (n = 2335)	CLcr 30–49 (n = 122)	CLcr ≥50 (n = 797)	CLcr 30–49 (n = 46)	Any CLcr ≥50 (n = 3132)	Any CLcr 30–49 (n = 168)	Any CLcr <30 (n = 13)	
Median age (year)	36.5	41.2	36.6	44.1	36.5	41.3	38.2	36.8
Male n (%)	860 (37)	33 (27)	252 (32)	10 (22)	1112 (36)	43 (26)	3 (23)	1160 (35)
Female n (%)	1475 (63)	89 (73)	545 (68)	36 (78)	2020 (64)	125 (74)	10 (77)	2156 (65)
Median CD4 cells/mm^3^	81.0	85.5	98.0	102.5	85.0	89.0	101.0	86.0
WHO disease stage, n	2335	122	797	46	3132	168	13	3316
Stage 2, n (%)	449 (19)	26 (21)	189 (24)	7 (15)	638 (20)	33 (20)	1 (8)	673 (20)
Stage 3, n (%)	1294 (55)	55 (45)	476 (60)	29 (63)	1770 (57)	84 (50)	8 (62)	1864 (56)
Stage 4, n (%)	592 (25)	41 (34)	132 (17)	10 (22)	724 (23)	51 (30)	4 (31)	779 (23)
HBV status, n	2335	122	797	46	3132	168	13	3316
Positive, n (%)	1236 (53)	65 (53)	443 (56)	24 (52)	1679 (54)	89 (53)	5 (38)	1774 (53)
Negative, n (%)	1097 (47)	57 (47)	352 (44)	22 (48)	1449 (46)	79 (47)	7 (54)	1537 (46)
Missing, n (%)	2 (<1)	0	2 (<1)	0	4 (<1)	0	1 (8)	5 (<1)
Median CLcr (measured by CGF) (mL/min)	85.4	44.3	81.8	44.9	84.4	44.4	27.4	82.8

ABC, abacavir; ART, antiretroviral therapy; CGF, Cockcroft-Gault formula; CLcr, creatinine clearance; CGF, Cockcroft Gault Formula; HBV, hepatitis B virus; 3TC, lamivudine; NVP, nevirapine; TDF, tenofovir; WHO, World Health Organization; ZDV, zidovudine.

^a^ Three participants with missing baseline CLcr are excluded from the total N.

A total of 94% (3126/3313) and 93% (3073/3313) of trial participants had data included in these analyses at Weeks 24 and 48, respectively. After Week 48, 71% (2349/3313) of DART participants, excluding all participants who were enrolled in the STI sub-study had data collected for at least one of the biochemistry test results (including bilirubin, alanine amino transferase and aspartate amino transferase) or CD4 cell counts at their Week 96 visit and were included in these analyses. Additionally, there were 653/3313 (20%) DART participants who were part of the STI sub-study. Of these, 225 participated before Week 96 and any data collected after the start of their first STI was not included. For the other 428 participants whose STIs occurred after Week 96, data collected through Week 96 (prior to the start of their STI) were also included in these analyses.

### Renal function

**Fig 1:**
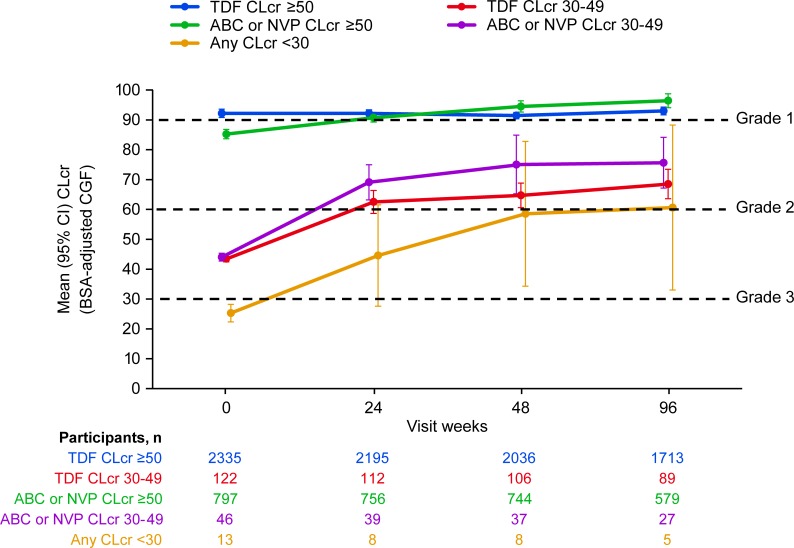
Mean (95% CI) Creatinine Clearance (CLcr) by Visit and Baseline CLcr (mL/min) Through Week 96. ABC, abacavir; BSA, body surface area; CGF, Cockcroft-Gault formula; CI, confidence interval; CLcr, creatinine clearance as calculated by BSA-adjusted CGF; NVP, nevirapine; TDF, tenofovir.

The mean change in CLcr over time and by treatment group is presented in Fig 1. Substantial increases in the mean CLcr (>25 mL/min) were observed over time among participants who entered the trial with CLcr 30–49 mL/min, while no increase or smaller mean changes in CLcr (<10.5 mL/min) were observed for participants who entered the trial with CLcr ≥50 mL/min.

### CD4+ cell counts

There were similar increases in CD4+ cell counts from baseline over time in all groups. The mean (and standard deviation; SD) change from baseline to Week 96 in CD4+ cells counts was 168 (125) cells/mm^3^ in the 3TC/ZDV + TDF baseline CLcr 30–49 mL/min -treated group, 181 (94) cells/mm^3^ in the 3TC/ZDV + ABC or NVP baseline CLcr 30–49 mL/min-treated group, 156 (119) cells/mm^3^ in the 3TC/ZDV + TDF baseline CLcr ≥50 mL/min -treated group and 180 (127) cells/mm^3^ in the 3TC/ZDV + ABC or NVP baseline CLcr ≥50 mL/min-treated group.

### Post hoc analysis of adverse events

Overall, 28% of participants experienced Grade 3–4 AEs, 9% reported SAEs and 5% of participants died during the 96-week treatment period (Table 3). These rates were broadly similar when comparing those who initiated ART with a CLcr 30–49 mL/min versus those who initiated ART with a CLcr ≥50 mL/min. The AE profile of 3TC/ZDV with ABC NVP or TDF in participants with baseline CLcr of 30–49 mL/min was comparable to the overall safety profile in the other treatment groups. Because the majority of Grade 3/4 AEs and SAEs occurred by Week 24, Grade 3/4 AEs that occurred by Week 24 were selected and summarized by baseline CLcr and Week 24 CLcr.

**Table 3:** Summary of Number of participants experiencing grade 3–4 AEs, SAEs or death by treatment and baseline CLcr (mL/min) groups through 96 weeks.

Event	TDF CLcr 30–49 (n = 122)	ABC or NVP CLcr 30–49 (n = 46)	Any ART			Overall^a^ (n = 3316)
			CLcr ≥50 (n = 3132)	CLcr 30–49 (n = 168)	CLcr<30 (n = 13)	
Grade 3–4 AEs n (%) through Week 96	41 (34)	14 (30)	850 (27)	55 (33)	7 (54)	912 (28)
SAEs n (%) through Week 96	12 (10)	7 (15)	261 (8)	19 (11)	2 (15)	283 (9)
Deaths n (%) through Week 96	10 (8)	5 (11)	136 (4)	15 (9)	4 (31)	155 (5)
Deaths n (%) through Week 96 with no associated SAEs	5 (4)	1 (2)	76 (2)	6 (4)	2 (15)	84 (3)

Table 4 presents a summary of the Grade 3–4 events which occurred in at least 2% of trial participants in any treatment group by system organ class and by specific AEs within these classes. Hematological AEs were the only system organ class where events were reported in ≥5% of trial participants in any treatment group, with neutropenia being the most frequent AE. The incidence of Grade 3–4 hematological AEs was comparable across treatment groups (21–24%), as was the incidence of neutropenia (16–18%) and anemia (7–9%). In addition to neutropenia and anemia with clinical symptoms, pancytopenia with possible bone marrow depression and thrombocytopenia were reported in ≤2% in at least one treatment group, but the incidence was comparable across treatment groups. The overall incidence of grade 3–4 AEs for all other system organ classes was comparable across treatment groups and the number of trial participants with specific events within each class were small.

**Table 4:** Summary of grade 3–4 AEs occurring in ≥2% of participants of any treatment group.

DART Defined System Organ Class Preferred Term^a^	TDF CLcr ≥50 (N = 2335)	TDF CLcr 30–49 (N = 122)	ABC or NVP CLcr ≥50 (N = 797)	ABC or NVP CLcr 30–49 (N = 46)
	n (%)			
Any Grade 3 or 4 event	632 (27%)	41 (34%)	218 (27%)	14 (30%)
Hematological				
Any event	518 (22%)	29 (24%)	171 (21%)	11 (24%)
Neutropenia	371 (16%)	22 (18%)	130 (16%)	8 (17%)
Anemia with clinical symptoms	181 (8%)	8 (7%)	53 (7%)	4 (9%)
Pancytopenia, bone marrow depression	11 (<1%)	2 (2%)	3 (<1%)	1 (2%)
Thrombocytopenia	8 (<1%)	1 (<1%)	1 (<1%)	1 (2%)
Cardiovascular				
Any event	29 (1%)	5 (4%)	6 (<1%)	1 (2%)
Deep venous thrombosis	7 (<1%)	0	6 (<1%)	1 (2%)
Hypotension/Shock/Toxic shock	4 (<1%)	2 (2%)	0	0
Hepatic				
Any event	29 (1%)	0	12 (2%)	1 (2%)
Lactic acidosis	14 (<1%)	0	3 (<1%)	1 (2%)
Gastrointestinal				
Any event	30 (1%)	5 (4%)	5 (<1%)	0
Vomiting	13 (<1%)	3 (2%)	2 (<1%)	0
Systemic^b^				
Any event	8 (<1%)	0	12 (2%)	0
Renal				
Any event	12 (<1%)	2 (2%)	1 (<1%)	0
Renal failure (chronic)	1 (<1%)	2 (2%)	0	0
Specific infections				
Any event	14 (<1%)	1 (<1%)	1 (<1%)	1 (2%)
*P falciparum* malaria	14 (<1%)0	0	1 (<1%)	1 (2%)
Lower respiratory tract				
Any event	6 (<1%)	2 (2%)	1 (<1%)	0
Other^c^				
Any event	3 (<1%)	0	0	1 (2%)
Overdose (not suicide attempt)	0	0	0	1 (2%)

ABC, abacavir; AE, adverse event; CLcr, creatinine clearance; NVP, nevirapine; TDF, tenofovir.

^a^ Per protocol, the first term was defined as the primary term and coded to the system organ class; any additional diagnoses, signs and symptoms were listed after the primary term and were not coded separately.

^b^ Systemic events as include events of hypersensitivity reaction, malaise, tiredness, acute febrile episode, dehydration, recurrent fever and Stevens-Johnson syndrome.

^c^ Other events included non-fatal trauma and overdose (not suicide attempt).

As shown in Fig 1, most participants experienced an improvement in CLcr, particularly among those with baseline CLcr <50 mL/min, and as their renal function improved, since lamivudine is largely excreted through the urine, would have therefore had a reduction in plasma 3TC concentrations over time. To determine whether safety events occurred more often among those participants whose CLcr remained <50 mL/min and thus had persistently higher lamivudine exposures, we compared the incidence of events between participants with different CLcr dynamics, focusing on those who had CLcr levels available at Week 24. As shown in Table 5, whilst numbers within some subgroups were small, the incidence of Grade 3–4 AEs and SAEs in the groups of participants whose baseline CLcr was 30–49 mL/min were broadly similar to that observed for the group of participants who entered the trial with CLcr ≥50 mL/min and had CLcr ≥50 mL/min at Week 24.

**Table 5:** Comparison of grade 3 and 4 AEs for trial participants grouped by baseline and week 24 CLcr^a^.

Event	Participants with Baseline CLcr 30–49 mL/min and Week 24 CLcr<50 mL/min	Participants with Baseline CLcr 30–49 mL/min and Week 24 CLcr≥50 mL/min^1^	Participants with Baseline and Week 24 CLcr ≥50mL/min^1^	Participants with Baseline CLcr ≥50mL/min and Week 24 CLcr <50 mL/min
N	36	115	2904	47
Any Grade 3 and 4 AEs by Week 24, n (%)	9 (25%)	31 (27%)	585 (20%)	20 (43%)
SAEs by Week 24	0 (0%)	3 (3%)	116 (4%)	7 (15%)

AE, adverse event; CLcr, creatinine clearance; SAE, serious AE.

^a^ The Week 24 sample date indicated on the laboratory results was used as the cutoff date for AE and SAE week 24 evaluation.

Overall, 283/3316 participants experienced SAEs (including 2 participants whose baseline CLcr was <30 mL/min). Table 6 summarizes the SAEs reported in ≥2% of participants in any treatment group. The most common event was anemia, although overall hematological events were comparable across groups (4% vs. 3%). For the other system organ classes, the incidence of individual SAEs by organ class or by specific event were comparable and ranged from 0 to ≤2% across the treatment groups except for the DART investigator defined category of “other”. Among those with baseline CLcr 30-49mL/min, SAEs were reported for 7 participants in the 3TC/ZDV/ABC (or NVP) group [including one case of *P*. *falciparum* malarial infection and two cases with unknown causes of death (4%)] and SAEs were reported for 12 participants in the 3TC/ZDV/TDF group (including 2 participants with chronic renal failure).

**Table 6:** Summary of serious adverse events by DART defined preferred terms reported in ≥2% of participants in any treatment group.

DART Defined System Organ Class Preferred Term^a^	TDF CLcr ≥50 (N = 2335)	TDF CLcr 30–49 (N = 122)	ABC or NVP CLcr ≥50 (N = 797)	ABC or NVP CLcr 30–49 (N = 46)
	n (%)			
Any event	185 (8%)	12 (10%)	77 (10%)	7 (15%)
Hematological				
Any event	78 (3%)	4 (3%)	20 (3%)	2 (4%)
Anemia	64 (3%)	3 (2%)	13 (2%)	2 (4%)
Cardiovascular				
Any event	19 (<1%)	2 (2%)	6 (<1%)	1 (2%)
Deep venous thrombosis	5 (<1%)	0	6 (<1%)	1 (2%)
Systemic^b^				
Any event	4 (<1%)	0	18 (2%)	0
Hypersensitivity reaction	2 (<1%)	0	18 (2%)	0
Specific infections				
Any event	14 (<1%)	1 (<1%)	1 (<1%)	1 (2%)
*P falciparum* malaria	14 (<1%)	0	1 (<1%)	1 (2%)
Skin				
Any event	0	0	15 (2%)	0
Rash, maculopapular	0	0	13 (2%)	0
Gastrointestinal				
Any event	9 (<1%)	1 (<1%)	3 (<1%)	1 (2%)
Peptic/gastric/duodenal ulcer	0	0	0	1 (2%)0
Renal				
Any event	9 (<1%)	2 (2%)	1 (<1%)	0
Renal failure (chronic)	1 (<1%)	2 (2%)	0	0
Other				
Any event	8 (<1%)	0	2 (<1%)	2 (4%)
Death (cause unknown)	6 (<1%)	0	0	2 (4%)

ABC, abacavir; CLcr, creatinine clearance; NVP, nevirapine; TDF, tenofovir.

^a^ Per protocol, the first term was defined as the primary term which was coded to the system organ class and when trial physicians reported additional diagnoses, signs and symptoms, these were listed after the primary term and were not coded separately.

^b^ Systemic events as defined by the DART team included events of hypersensitivity reaction, febrile episode and Stevens-Johnson syndrome.

**Fig 2:**
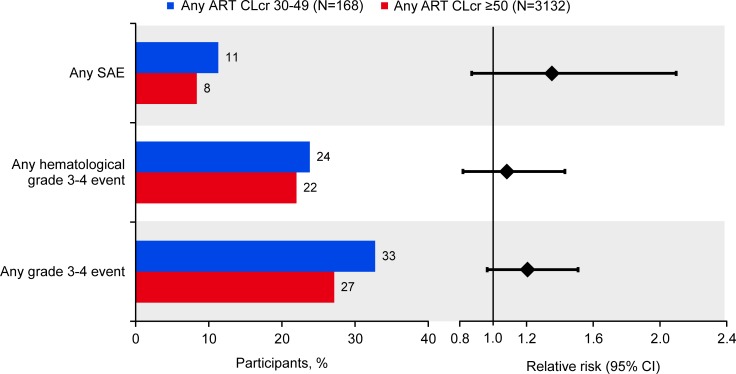
Common Adverse Events and Relative Risks for Any ART-Treated Participants With Baseline CLcr 30–49 mL/min vs Those With Baseline CLcr ≥50 mL/min. ART, antiretroviral therapy; CI, confidence interval; CLcr, creatinine clearance; SAE, serious adverse event.

In Fig 2, the AE event rates for participants treated with any ART with baseline CLcr 30–49 mL/min and those with baseline CLcr ≥50 mL/min are presented in the left panel and relative risks and 95% confidence intervals (CI) are presented on the right panel. None of the AE event rates were considered to be statistically different between the two groups as the 95% CI ranges encompassed 1.

### Hematology

The change from baseline to Week 96 for the hematology parameters (Table 7) in participants with baseline CLcr 30–49 mL/min were very similar to the change seen in participants with baseline CLcr ≥50 mL/min, except for hemoglobin, which had higher mean increases in the participants with CLcr 30–49 mL/min.

**Table 7:** Summary of Mean Change in Hematology Laboratory Results from Baseline to Week 96 by Treatment and Baseline CLcr (mL/min) Categories.

Variable	TDF CLcr≥50 (N = 2335)	TDF CLcr 30–49 (N = 122)	ABC or NVP CLcr≥50 (N = 797)	ABC or NVP CLcr 30–49 (N = 46)
Number of participants at Week 96	1713	90	582	27
Mean change in hemoglobin (g/dL) (min, max)	1.37 (−6.4, 9.4)	1.98 (−7.6, 6.0)	1.27 (−6.4, 6.8)	1.89 (−1.2, 7.2)
Number of participants at Week 96	1709	90	582	27
Mean change in lymphocytes (x10^3^ cells/μL) (min, max)	0.18 (−3.8, 5.4)	0.11 (−2.5, 2.8)	0.14 (−2.7, 4.8)	0.22 (−0.8, 2.0)
Number of participants at Week 96	1711	90	582	27
Mean change in mean corpuscular volume (femtoliters/cell) (min, max)	14.5 (−54.5, 78.9)	17.8 (−16.0, 79.4)	18.9 (−12.5, 86.1)	19.2 (−3.0, 39.0)
Number of participants at Week 96	1705	90	578	27
Mean change in neutrophils (x10^3^ cells/μL) (min, max)	0.00 (−11.5, 10.1)	0.02 (−7.2, 6.6)	0.00 (−6.2, 5.8)	−0.01 (−1.6, 1.8)
Number of participants at Week 96	1710	90	581	27
Mean change in platelets (x10^3^ cells/μL) (min, max)	55.5 (−367.0, 543.0)	55.8 (−189.0, 328.0)	40.5 (−467.0, 323.0)	14.6 (−201.0, 166.0)
Number of participants at Week 96	1711	90	582	27
Mean change in white blood cells (x10^3^ cells/μL) (min, max)	0.17 (−13.4, 13.6)	−0.09 (−8.0, 6.1)	−0.02 (−8.8, 6.7)	0.07 (−1.8, 3.5)

ABC, abacavir; CLcr, creatinine clearance; NVP, nevirapine; TDF, tenofovir.

A small number of participants had Grade 3 or 4 hematological toxicities (Table 8), and these were generally seen across both main CLcr sub-groups.

**Table 8:** Maximum hematology laboratory toxicity results over 96 Weeks by treatment and baseline CLcr (mL/min) categories.

Value	TDF CLcr≥50 (N = 2335)	TDF CLcr 30–49 (N = 122)	ABC or NVP CLcr≥50 (N = 797)	ABC or NVP CLcr 30–49 (N = 46)
Hemoglobin (g/dL)				
Grade 3 n (%)	8 (<1%)	0	3 (<1%)	1 (2%)
Grade 4 n (%)	19 (<1%)	2 (2%)	7 (<1%)	0
Neutrophils (x 10^3^cells/μL)				
Grade 3 n (%)	228 (10%)	15 (12%)	84 (11%)	6 (13%)
Grade 4 n (%)	112 (5%)	9 (7%)	28 (4%)	4 (9%)
Platelets (x 10^3^cells/μl)				
Grade 3 n (%)	17 (<1%)	1 (<1%)	9 (1%)	2 (4%)
Grade 4 n (%)	3 (<1%)	0	2 (<1%)	0
White blood cells (x 10^3^cells/μL)				
Grade 3 n (%)	119 (5%)	10 (8%)	33 (4%)	1 (2%)
Grade 4 n (%)	2 (<1%)	1 (<1%)	0	1 (2%)

ABC, abacavir; CLcr, creatinine clearance; NVP, nevirapine; TDF, tenofovir.

### Clinical chemistry

Overall, the maximal toxicities determined by measurements of alanine aminotransferase (ALT), aspartate aminotransferase (AST), serum creatinine and bilirubin reported across the treatment and CLcr groups were similar, with very few Grade 3 and 4 laboratory toxicities (<1%) and no trend of increase in toxicities grades over time.

## Discussion

The baseline demographics of the DART trial population, including the median enrollment CD4 cell count of 86 cells/mm^3^, were indicative of a population which was more strongly immunosuppressed at initiation of ART as compared with ART-naïve HIV-positive trial participants in resource-rich countries [6, 7]. As seen with other analyses from the DART trial [8, 9], there were greater increases in the mean creatinine clearance for participants who enrolled with impaired renal function (CLcr 30–49 mL/min) over 96 weeks ART, irrespective of treatment group, compared to participants who had CLcr ≥50 mL/min at enrollment, possibly due to a direct positive effect of treatment on renal function. There were 21/3316 trial participants with renal SAEs over the 5- year DART trial period; all 21 participants were either on or had previously been taking TDF at the time of the event [9].

The incidence of overall Grade 3–4 AEs and for specific hematological, hepatic and gastrointestinal events over 96 weeks of treatment were comparable between participants who had moderate renal impairment (CLcr 30–49 mL/min) at baseline and those with better renal clearance at baseline CLcr (≥50 mL/min). The maximal treatment-emergent hematological and chemistry toxicities over 96 weeks of treatment were also comparable across these two renal function groups. Although the overall percentages of any grade 3 /4 hematological events were comparable for participants with CLcr 30–49 mL/min vs those with CLcr ≥50 mL/min (Fig 2), rates in participants with moderate renal impairment were somewhat higher, likely due to the poorer health typically observed in patients with this condition. Finally, by Week 24, the majority (76%) of trial participants with baseline CLcr between 30–49 had normalization of renal function (to ≥50 mL/min) following the initiation of ART. HIV-associated nephropathy is a known complication of HIV infection resulting in impaired renal function; however, renal function will generally improve after ART initiation. Older guidelines recommended (prior to current guidelines, which recommend treatment of HIV regardless of disease stage) early use of combination ART to decrease risk factors associated with HIV-associated nephropathy [10, 11]. For the subset of participants with baseline CLcr between 30-49/mL/min whose Clcr had not normalized by Week 24, compared to participants whose CLcr was ≥50 mL/min at Week 24, there were no striking differences observed in the rates of Grade 3–4 AEs and SAEs.

There are several limitations of this *post hoc* data analysis. The sample size for trial participants with baseline CLcr of 30–49 mL/min was small, relative to the overall DART trial population. In keeping with the trial protocol, routine HIV-1 RNA and resistance testing were not prospectively undertaken and therefore treatment switches were guided by immunological (CD4+ cell counts) (LCM group only) and clinical parameters only. Limited clinical safety data (Grade 3 and 4 events only) were collected and used to classify AE events using a coding system developed by the DART Team of investigators based on diseases likely to occur in an African setting. Data from some of the 225 participants who underwent planned ART interruptions were excluded from this *post hoc* analysis after the start of the interruption as their inclusion would have made interpretation of AEs difficult. Despite these limitations, our data provide useful information regarding the safety of the use of a 3TC-containing FDC in patients with CLcr 30–49 mL/min compared with the safety of 3TC-containing FDC in patients with CLcr ≥50 mL/min.

There have been two recent publications on the use of higher-than-recommended doses of 3TC in patients with renal impairment. One study enrolled 34 HIV-positive patients with varying degrees of renal function and used physiologically-based pharmacokinetic modeling to simulate drug concentrations over time using areas under the curve (AUCs) and drug profiles by CLcr [12]. The authors noted that at their facility, 3TC is generally not dose-adjusted until CLcr <30 mL/min and that 100–150-mg 3TC tablets daily are used in HIV-positive patients on hemodialysis (HD). The observed 3TC peak serum concentrations (Cmax) values were comparable across the CLcr 30–49, 15–29, and 0–15 mL/min subgroups and the simulated 3TC AUC values were consistent with historical data, with fold-errors between 0.5 and 2.0. No adverse effects were reported, and all lactic acid levels were within normal limits. A second case series, in six patients positive for HIV with end-stage renal disease who had been maintained on intermittent hemodialysis, evaluated outcomes following a switch to the fixed-dose single tablet regimen of abacavir/lamivudine/dolutegravir [13]. No further decline in immune function was noted and the regimen was well tolerated; only one patient had an AE (nausea), which resolved without drug discontinuation.

## Conclusions

In this descriptive *post hoc analysis* of clinical trial data up to 96 weeks of observation, no new or additional risks associated with the use of a standard dose of a 3TC or a 3TC-containing FDC in participants with moderate renal impairment (with CLcr levels of 30–49 mL/min) compared to participants with CLcr ≥50 mL/min were suggested. Though *post hoc* analyses are intrinsically limited, the results of this analysis are consistent with recent reports from the literature regarding the safety of a standard 3TC dose when used in HIV-positive patients with moderate renal impairment.

## Supporting information

S1 File Study protocol. (PDF)
