# Supplementary material for: Changes over time in creatinine clearance and comparison of emergent adverse events for HIV-positive adults receiving standard doses (300 mg/day) of lamivudine-containing antiretroviral therapy with baseline creatinine clearance of 30–49 vs ≥50 mL/min
Source: PLoS One. 2019 Nov 14;14(11):e0225199. doi: 10.1371/journal.pone.0225199 (PMC6855468; doi:10.1371/journal.pone.0225199)
Supplement: S1 File — (PDF) [file pone.0225199.s001.pdf]

Development of AntiRetroviral Therapy in Africa

ACRONYM: DART

Full Title of trial:

A randomised trial of monitoring practice and structured treatment interruptions in the management of antiretroviral therapy in adults with HIV infection in Africa

ISRCTN13968779

Protocol number 1.4

Protocol date 29 March 2007

PPD [redacted]

.... 17/4/07 ....

Auth[redacted]ised signature Date

Name Professor [redacted]

(Chair of TSC for final protocols and amended final protocols)

Name of person authorised to sign final protocol and protocol amendments for the sponsor (Chair of TSC)

Professor PPD
Department of Sexually Transmitted Diseases
Mortimer Market Centre
off Capper Street
London WC1E 6AU

PPD
Tel: PPD
Fax: PPD

Trial Sponsor
Medical Research Council (MRC, UK)

Principal Investigators and Co-PIs (in bold) and Investigators responsible for conducting trial

Professor PPD
PPD

PPD
Tel: PPD
Fax:

Professor PPD
Dr PPD **(Co-PI)**
Dr PPD (Trial Manager)
PPD
PPD
PPD Uganda.

PPD
Tel: PPD
Fax:

Professor PPD

Dr PPD **(Co-PI)**

Dr PPD (Project Leader)

PPD (Trial Manager)

PPD
PPD
PPD Uganda

PPD

Tel: PPD
Fax:

Prof PPD
Prof PPD
PPD
PPD Uganda

PPD

Tel: PPD
Fax:

Professor [redacted]
Professor [redacted] **(Co-PI)**
Prof [redacted]
Dr [redacted] **(Project Leader)**

[redacted]

[redacted]
[redacted] Zimbabwe
[redacted] (Trial Manager)

Tel: [redacted]
Fax: [redacted]
[redacted]

Professor [redacted]
Professor [redacted] **(Co-PI)**
Professor [redacted]

[redacted]

Tel: [redacted]
Fax: [redacted]

Trial manager and monitors

[redacted]

Tel: [redacted]
Fax: [redacted]

Dr [redacted]

[redacted]

The trial will be conducted in compliance with the protocol, GCP and regulatory requirements.

Trial statisticians

Professor [redacted]

[redacted]
Tel: [redacted]

Dr [redacted]

Tel: [redacted]

MRC Clinical Trials Unit
222 Euston Road
London NW1 2DA

Fax: [redacted]

Trial Funders

The trial is funded by the Medical Research Council (MRC UK), Rockefeller Foundation, US and Department for International Development, UK (DFID) and the drugs are supplied by Boehringer Ingelheim, Gilead, GlaxoSmithKline and Abbott Labs.

SUBSTANTIVE CHANGES FROM DART PROTOCOL V1.3 TO V1.4 (important changes in bold)

- **Extension of the trial by one calendar year: the DART trial will have a nominal end date of 31 December 2008, though in fact there will be a phased transition to national programmes from late 2008 and the last DART clinic visit will occur in the first quarter of 2009.**
- **The bPI monotherapy section of the second-line randomisation that was added in version 1.3 is removed again in this amendment, having been transferred (largely unchanged, except for the addition of separate informed consent at the point of randomisation to bPI monotherapy maintenance or continued combination ART) to a separate Protocol (the SARA study).**
- **Added an explicit statement that DART participants may co-enrol in any other study provided this does not compromise the integrity of the DART trial. Specifically the restrictions relating to laboratory monitoring must be maintained, section 6.5, page 31.**
- **Addition of sample size calculations for the trial extension, section 10.1, page 43.**
- **Amendment of sample size calculations for second line randomisation, section 10.1, page 43.**
- **Addition of Aluvia to Appendix 3.0 (details of ARVs), page 66.**
- **Amendment of the patient information sheet for the second-line feasibility study to remove bPI monotherapy, Appendix 6.4, page 83.**
- **Addition of Abbott as a donor of ARVs (Aluvia) for second-line.**

SUBSTANTIVE CHANGES FROM DART PROTOCOL V1.2 TO V1.3 (important changes in bold)

- **Termination of STI randomisation throughout. Note that several amendments relating to the STI randomisation for safety had already been implemented and were in draft for Protocol amendment when the STI randomisation was terminated on 15 March 2006. These changes are included for completeness but are no longer being applied as the whole STI arm has been closed with all participants moving to the CT arm.**
- Explicit statement of MRC as Sponsor of the DART Trial.
- Change of contact details and personnel.
- **Amendment of Trial Summary – Abstract, page 13, and Schema, page 14, to include feasibility second line randomisation.**
- **Amendment of flow sheets, section 1.3, page 16**

- **Amendment of rationale to include second line randomisation feasibility study, section 2.5, page 24.**
- **Amendment of DART trial objectives – that STIs will only be evaluated during first line ART, section 3.1, page 25**
- **Addition of section on second line feasibility study to objectives, section 3.4, page 26.**
- **Addition of endpoints for second line feasibility study, section 4.0, page 26.**
- **Amendment of STI/CT randomisation throughout to allow randomisation at week 76 for patients not randomised at week 52 for any reason.**
- **Amendment to criteria for modifying the STI regimen and restarting ART early, section 7.5.2, page 36.**
- **Amendment of criteria for deferring STI - section 7.5.3, page 36.**
- **Addition of information on second line ART to section 2.4, page 22.**
- **Addition of 2nd line ART feasibility randomisation to design, section 5.1.4, page 28.**
- **Amendment of section 7.3.1, page 31, to include 2nd line feasibility randomisation.**
- **Addition of details of 2nd line feasibility randomisation - section 7.3.4, page 33.**
- **Amendment of** Table 5**, page 39, for second line regimens.**
- **Amendment of section 7.6.2, page 37 on switching at failure of first line to include option to switch when the CD4 count is between 100 and 50 cells/mm^3 (LCM only)**
- **Amendment of section 8.1.2, page 40 for second line ART**
- **Addition of section on second line randomisation feasibility study to sample size, section 10.1, page 43**
- **Addition of section on second line randomisation feasibility study to analysis plan, section 10.2, page 46**
- **Additional references, section 14.0, page 47**
- **Addition of lactate, cardiomyopathy and lipodystrophy to AE grading table, appendix 4.1,page 70**
- **Addition of Patient Information Sheet for second line randomisation feasibility study - appendix 6.4, page 83**
- **Addition of Consent Form for second line randomisation feasibility study – appendix 6.5, page 87**
- **Update of section on post-trial issues, Appendix 9.0, page 90**

SUBSTANTIVE CHANGES FROM DART PROTOCOL V1.1 TO V1.2 (important changes in bold)

- Change of various contact details.
- **Amendment of CD4 threshold for STI randomisation to 300 cells/mm^3, throughout.**
- **Amendment of time for STI randomisation to week 52 (using week 48 CD4 count for eligibility), throughout.**
- **Amendment of recruitment target for STI/CT randomisation to 600, throughout.**
- **Recruitment target revised to 3300, throughout.**
- **Recruitment period for CMO/LCM extended into 2nd year, throughout.**
- **Recruitment period for STI/CT extended into 3rd year, throughout.**
- Trial schema revised, section 1.2, page 14.
- **Flow sheets revised for STI changes, section 1.3, page 16.**
- **Flow sheets revised to make 12 weekly plasma storage mandatory and addition of plasma storage at (or near) time of delivery, section 1.3, page 16.**
- NORA will not take place in Zimbabwe, throughout.
- **Addition of specific eligibility criteria for STI randomisation, section 7.3.3, page 32.**
- **Addition of intensive monitoring of first 100 patients randomised to STI/CT, section 7.3.3, page 32.**
- **Revision of criteria for restarting ART early during an STI, section 7.5.2, page 36.**
- **Revision of criteria and procedure for deferring an STI, section 7.5.3, page 36.**
- **Revision of CD4 threshold and time on ART for resuming STIs after switch to second line, removal of recurrent bacterial infection for clinical reason for switching and reduction of time for repeating CD4 counts if <50, section 7.6.2, page 37.**
- **Updating of sample size calculations, section 10.1, page 43.**

SUBSTANTIVE CHANGES FROM DART PROTOCOL V1.0 TO V1.1 (important changes in bold)

- Change of PI details at PPD
- Addition of contact details for Project Leader at PPD
- Addition of Investigators at PPD and PPD
- Update of various contact details.
- Addition of details of trial managers at sites and at CTU.

- **Section 4.2: addition of 2 extra secondary endpoints.**
- Section 5.1.2: CD4 monitoring should not be open for CMO patients in the STI pilot
- **Section 6.2 point 5: replacement of WBC as example exclusion criterion with neutrophils <0.50x10^9/l.**
- **Section 6.3: inclusion of PPD site in the NORA substudy.**
- Section 7.1: removal of cell percentages from required cell counts.
- Section 7.3.1: CMO/LCM stratification corrected to 0-99 and 100-199 CD4 not 1-99 and100-200.
- Section 7.3.1: STI/CT stratification by week number at entry.
- Section 7.3.3: remove requirement to fax CTU.
- Section 7.3.3: extend period on other ARVs from 5 to 7 days on stopping NVP.
- Section 7.3.6: clarification about families and NORA.
- **Section 7.4: change to reporting of grade 4 biochemistry in CMO arm.**
- Section 7.5.1: extend period on other ARVs from 5 to 7 days on stopping NVP.
- Section 7.6.2: allowance for some clinical discretion about reasons for switching ART.
- Section 7.9: clarification of reporting of protocol violations.
- Section 7.9.1: clarification of definition of SAEs and reporting.
- Section 8.1 Table 5: second line ART specified as containing 2 NRTIs rather than limiting to ddI+d4T.
- **Section 8.1.1: revision to allow NORA in PPD**
- Section 8.1.2: second line ART specified as containing 2 NRTIs rather than limiting to ddI+d4T and removal of reference to Zimbabwe as only country where NVP will be first line.
- Sections 9.1 & 9.4: a subgroup of TSC (not of DSMC) will monitor STI pilot and STI.
- Section 9.5: Endpoint Review Committee structure revised.
- Section 12.0: requirement for locked file cabinets replaced with locked locations.
- Section 14.0 ref 10: update of reference to WHO guidelines from draft to final.
- **Appendix 1.0: monitoring of STI pilot revised.**
- Appendix 1.5: pregnancy test corrected to week 48 from 52.
- Appendix 2.4: NORA schema clarified.
- **Appendix 2.5.1: NORA primary endpoint revised.**
- **Appendix 2.5.2: NORA secondary endpoints clarified.**
- **Appendix 2.6.2 pt 5: replacement of WBC as example exclusion criterion with neutrophils <0.50x10^9/l.**

- Appendix 2.9: management of adverse events in NORA expanded.
- Appendix 2.9.5: revision of safety monitoring in NORA.
- Appendix 2.11: NORA flowsheet revised - removal of extra pregnancy test at week 12 and adherence assessment at week 0.
- **Appendix 2.12: NORA patient information sheet revised for inclusion of** PPD
- **Appendix 2.13: NORA consent revised for inclusion of** PPD
- Appendix 3.0: removal of restriction for TDF to be taken with food.
- Appendix 3.0: footnote added to ZDV contraindications to note revised eligibility criteria on neutrophils.
- Appendix 5.0: correction of performance scale 3 to bed-ridden <50% of time.

TABLE OF CONTENTS

1.0 Summary....13
1.1 Abstract....13
1.2 Trial Schema....14
1.3 Flow Sheets....16
1.3 Flow Sheets....16

2.0 Background and Rationale....20
2.1 Introduction....20
2.2 Monitoring for efficacy and toxicity....20
2.3 Structured Treatment Interruptions (STIs)....21
2.3.1 STI randomisation termination....21
2.4 Antiretroviral Therapy Regimens....22
2.5 Rationale....24

3.0 Objectives....25
3.1 Main DART Trial Objectives....25
3.2 STI Pilot Study Objectives....25
3.3 Abacavir Safety Substudy Nevirapine OR Abacavir (NORA) Objectives....25
3.4 Second line feasibility study objectives....26

4.0 Endpoints....26
4.1 Primary Endpoints (CMO/LCM and STI/CT):....26
4.2 Secondary Endpoints (CMO/LCM and STI/CT):....26
4.3 Primary Endpoints (second-line randomisation feasibility study):....27
4.4 Secondary Endpoints (second-line randomisation feasibility study):....27

5.0 Design....27
5.1 Type of design....27
5.1.1 Randomisation to CMO or LCM arm....27
5.1.2 STI Pilot....27
5.1.3 STIs or continuous ART....27
5.1.4 Feasibility randomisation for second line ART for those failing first line ART....28
5.2 Antiretroviral Therapy....29

6.0 Patient Population....29
6.1 Inclusion Criteria (CMO/LCM)....29
6.2 Exclusion criteria (CMO/LCM)....29
6.3 Number and source of subjects (CMO/LCM and STI/CT)....30
6.4 Number and source of subjects (second-line feasibility study randomisation)....30
6.5 Co-enrolment in other studies....31

7.0 Procedures and Management of Subjects....31
7.1 Screening Procedure for CMO/LCM....31
7.2 Baseline procedure (Week 0 for CMO/LCM)....31
7.3 Randomisation and Enrolment....31
7.3.1 Randomisation List....31
7.3.2 First Randomisation to CMO or LCM....32
7.3.3 Second Randomisation – STIs or continuous ART....32
7.3.4 Third randomisation – second line regimen feasibility study....33
7.3.5 Pregnancy....34

7.3.6 Enrolment of family members into the trial 34
7.4 Follow-up Evaluations 34
7.5 Management of patients allocated to the STI arm 36
7.5.1 Timing of STIs 36
7.5.2 Modifying the STI regimen 36
7.5.3 Cessation of STIs 36
7.5.4 Deferring STI during follow-up 37
7.5.5 Termination of the STI study 37
7.6 Changing ART 37
7.6.1 Substituting for Toxicity 37
7.6.2 Switching for failure of first-line therapy 37
7.7 Assessment of adherence 38
7.8 Withdrawal from allocated strategy 38
7.9 Recording and reporting of Adverse Events, Death, HIV progression and protocol violations 38
7.9.1 Serious Adverse Events (SAE): 39

8.0 Drug Regimens 39
8.1 Antiretroviral Therapy 39
8.1.1 First-line antiretroviral drugs 40
8.1.2 Second-line antiretroviral drugs 40
8.1.3 Provision of trial drugs 40
8.1.4 Modification of therapy for toxicity 40
8.2 Prophylaxis against Opportunistic Infections 41
8.3 Medications not permitted/ Precautions 41
8.4 Data on concomitant medications 41

9.0 Management of the Trial 41
9.1 Trial Steering Committee 41
9.2 Trial Management Groups 42
9.3 Data Management and Monitoring 42
9.4 Data and Safety Monitoring Committee (DSMC) 43
9.5 Endpoint Review Committee 43

10.0 Statistics 43
10.1 Sample Size 43
10.2 Analysis Plan 46

11.0 Regulatory/ Ethics Approval 46

12.0 Confidentiality 46

13.0 Publication 47

14.0 References 47

Appendices

Appendix 1.0 Structured Treatment Interruptions (STI) Pilot Study 49
1.1 Monitoring of individual patients 49
1.2 Monitoring of the group data 49
1.3 Guidelines for management during STI pilot. 50
1.4 Guidelines for the DSMC and Steering Committees. 50

1.5 Flow Sheet for STI pilot 51

Appendix 2.0 Nevirapine OR Abacavir (NORA) Substudy 52

2.1 Objective 52
2.2 Background and Rationale 52
2.2.1 Hypersensitivity reaction to Abacavir..52
2.2.2 Adverse reactions to NVP...53
2.3 Trial Design 53
2.4 NORA Trial Schema 54
2.5 Endpoints 54
2.5.1 Primary endpoint:..54
2.5.2 Secondary Endpoints...55
2.6 NORA Criteria for Inclusion/Exclusion 55
2.6.1 Inclusion Criteria..55
2.6.2 Exclusion criteria 55
2.7 Sample size 56
2.8 Analysis Plan 56
2.9 Management of Adverse reactions 56
2.9.1 Management of reactions to combivir 56
2.9.2 Management of reactions to blinded drug...56
2.9.3 Management of nevirapine reactions 57
2.9.4 Management of abacavir reactions 57
2.9.5 Safety monitoring...57
2.10 Hypersensitivity reaction to abacavir and management of safety - as provided by GSK. 58
2.10.1 Description of hypersensitivity to Abacavir 58
2.10.2 Information for patients 59
2.10.3 Medical management for possible hypersensitivity to abacavir 59
2.10.4 Medical management for Abacavir rash not accompanied by systemic symptoms 60
2.10.5 Fever and hypersensitivity to Abacavir 60
2.10.6 Special considerations following an interruption of abacavir therapy........60
2.11 NORA SUBSTUDY FLOW SHEET 62
2.12 DART NORA Substudy Patient Information sheet 63
2.13 DART NORA Substudy Patient consent form 65

Appendix 3.0 Details of Antiretroviral Therapy..66

Appendix 4.0 Toxicity Grading and Management 70

4.1 Table of Common Toxicity Criteria 70
4.2 Table of Clinical Signs, Symptoms, Monitoring and Management of Symptoms of Serious Adverse Effects of Antiretroviral Drugs that Require Drug Discontinuation (adapted from WHO guidelines [ref 10] Annex 11B) 74
4.3 Guide to management of toxicities 75
4.3.1 Grade 1 (clinical or laboratory (LCM arm only)):.......................................75
4.3.2 Grade 2 (clinical or laboratory (LCM arm only)):.......................................75
4.3.3 Grade 3 (clinical or laboratory (LCM arm only)):.......................................75
4.3.4 Grade 4*(clinical or laboratory):...75

Appendix 5.0 Clinical progression and WHO Definitions............................76

Appendix 6.0 Patient Information sheet and Consent Forms (Templates)..77

6.1 Patient information sheet for DART study 77
6.2 Consent for Screening for the DART Trial 81
6.3 Patient consent form for DART Trial 82
6.4 Patient information sheet for the second line randomisation feasibility study 83
6.5 Patient consent form for the feasibility second line randomisation 87

Appendix 7.0 Handling and Storage of Specimens....................................88
7.1 Bloods 88
7.1.1 Processing of EDTA blood for Plasma and cell store..................................88
7.1.2 Storage of samples..88

Appendix 8.0 Adherence protocol..89

Appendix 9.0 Post DART Trial issues...90

Tables

Table 1	Laboratory and Clinical Monitoring (LCM) group (+/- STI co-enrolment)	16
Table 2	LCM group (+/- STI co-enrolment): weeks 84-180	17
Table 3	Clinical Monitoring Only (CMO) Group (+/- STI co-enrolment)	18
Table 4	CMO Group (+/- STI co-enrolment): weeks 84 - 180	19
Table 5	First and second-line drug regimens for DART (For details see Appendix 3.0, page 67)	39
Table 6	Guidelines for Substituting for Toxicity in First-Line	41
Table 7	STI Pilot for 100 patients (completed 24 weeks in CMO or LCM and with CD4 cell counts at week 24 of 200 cells/mm^3 or higher.	51
Table 8	NORA flow sheet	62

1.0 Summary

1.1 Abstract

The DART protocol describes an open-label randomised trial evaluating two strategic approaches for management of antiretroviral therapy (ART) in symptomatic HIV infected adults in Africa. The first strategy compares **clinical monitoring only** (CMO) with **laboratory plus clinical monitoring** (LCM). The second approach compares structured treatment interruptions (STIs: 12 weeks on, 12 weeks off ART) with continuous ART in patients who achieve CD4 cell counts ≥ 300 cells/mm^3 after 48 weeks on continuous ART.

Eligible patients will have symptomatic HIV disease (WHO stage 2, 3 or 4) and CD4 cell counts <200 cells/mm^3, no prior ART and no clinical or laboratory abnormalities contra-indicating start of ART. 3300 patients will be enrolled over 1-2 years into the CMO/LCM comparison from 3 African sites (2 in Uganda, 1 in Zimbabwe) and followed for 3-5 years. In both CMO and LCM arms, patients will have haematology and liver function tests performed but the results will not be returned to clinicians caring for patients in CMO arm unless indicative of a grade 4 adverse event. CD4 counts will be performed both in CMO and LCM arms with results not returned to physicians in the CMO arm but monitored independently by a subgroup of the Data Safety and Monitoring Committee (DSMC).

Following the pilot STI study in 100 patients, it is expected at least 600 will achieve CD4 ≥300 cells/mm^3 by 48 weeks after trial entry and will undergo a second randomisation to structured treatment interruption (STI) or to continuous ART. The second randomisation will open after a non-randomised pilot study of a 100 patients assigned to STIs (see Appendix 1.0, page 49) has been completed and the DSMC and Trial Steering Committees have assessed STIs as safe. During the pilot study and during the first phase of the STI randomisation, all patients (in both CMO and LCM arms) will undergo monthly CD4 cell monitoring.

Patients will initially receive zidovudine (ZDV) and lamivudine (3TC) in combination (combivir, CBV) plus tenofovir (TDF, 2,400 patients) or nevirapine (NVP, 300 patients). In addition, 600 patients enrolled early in the trial will be offered co-enrolment (for which they will consent separately) to a double blind 24-week comparison of the safety of abacavir (ABC) versus NVP for first line therapy (see Appendix 2.0, page 52). These patients will continue to receive ABC or NVP, respectively, after the blinded period is over. For toxicity or intolerance of any individual drug, another drug from the same class will be substituted.

The primary efficacy endpoint will be progression to a new WHO HIV stage 4 disease or death. The decision to change to second-line ART will be based on clinical criteria alone for the CMO arm and on clinical plus laboratory criteria for the LCM arm. Second-line ART consisting of a ritonavir boosted protease inhibitor (bPI) plus (initially at least) either nucleoside or non-nucleoside reverse transcriptase inhibitors or both will be provided according to the feasibility study randomisation detailed in section 7.3.4, page 33 (anyone not consenting to enter this feasibility study will still be offered a bPI containing second line regimen). Patients who fail the second-line regimen will be offered the best combination available from the first and second line drugs.

DART is a six-year trial. Recruitment into the trial will take place over two years for the first randomisation to the 2 monitoring strategies, with follow-up of 4 years after the last patient is randomised. For the second randomisation to STIs or continuous ART, recruitment will occur during the second and third years, to be completed by the end of the third year and follow-up will be for up to 2 years after the last patient is randomised.

1.2 Trial Schema

CMO/LCM and STI/CT

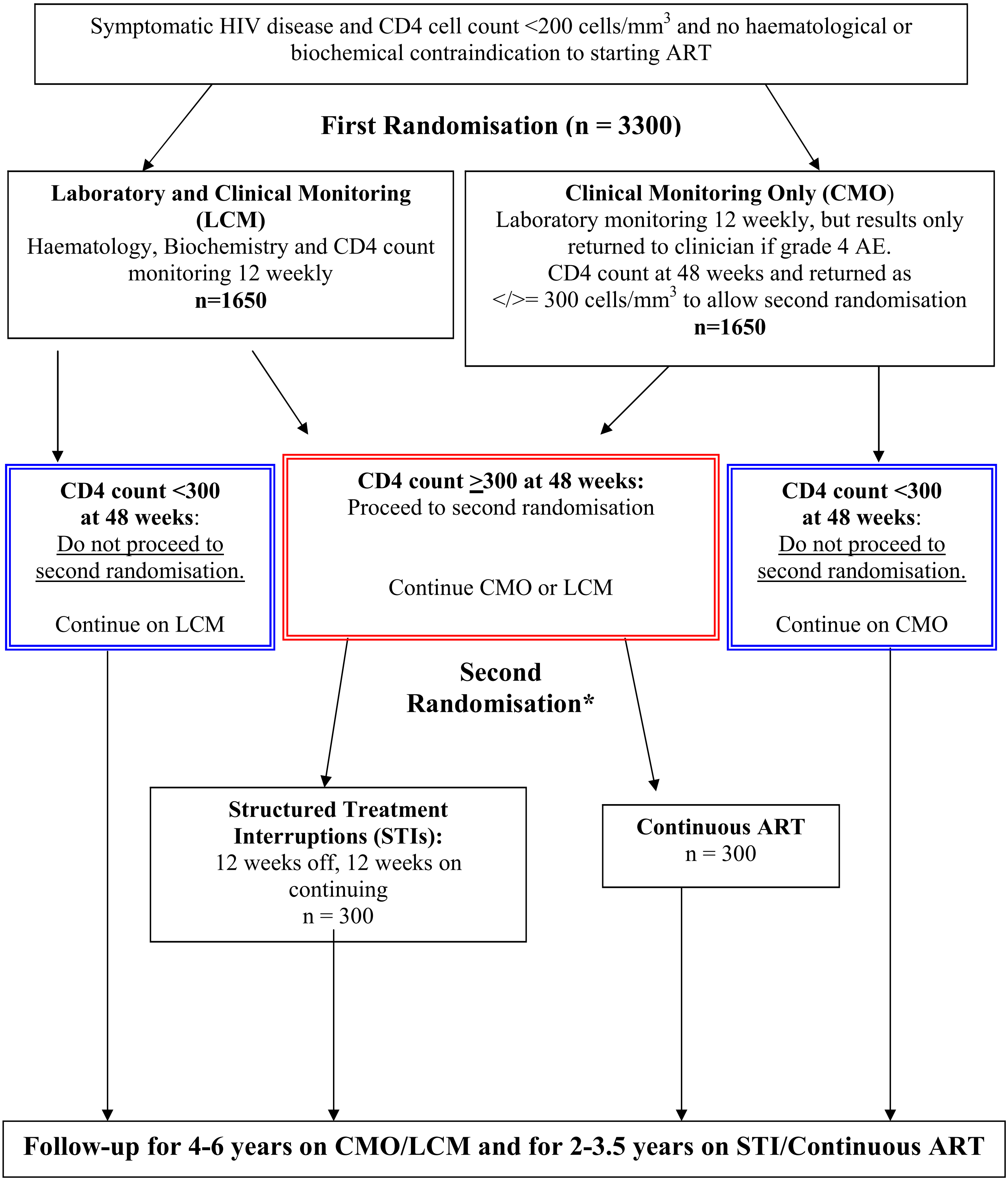

* Once second randomisation has opened after STI pilot study. **Note STI randomisation terminated 15th March 2006**

Second-line feasibility study randomisation:

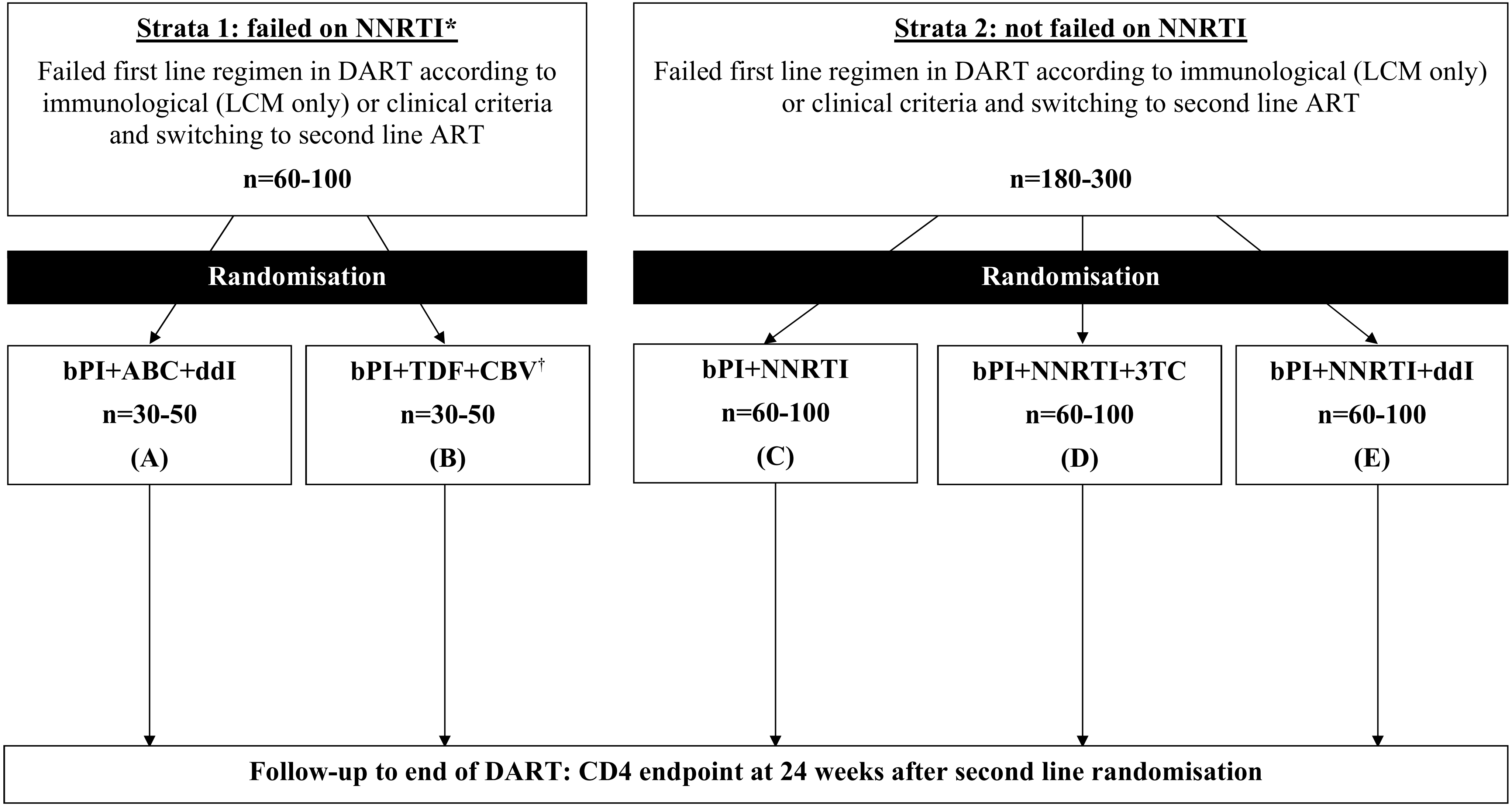

* Note: any patient who did not disclose prior NNRTI experience before entering DART & is considered to have failed immunologically and/or clinically whilst taking NNRTI should be enrolled in strata 1.

† patients with previous adverse reaction to ZDV will receive bPI+TDF+3TC only

1.3 Flow Sheets

Note that the STI arm has been terminated (15 March 2006) so no STIs will be taken after this date.

Table 1 Laboratory and Clinical Monitoring (LCM) group (+/- STI co-enrolment)

EVENTS	WEEK IN TRIAL																						
Doctor/Nurse visit * ▒<br>Nurse visit □<br>Doctor only visit * ■	Screening Week-2	Start therapy Week 0	2	4	8	12	16	20	24	28	32	36	40	44	48	52	56	60	64	68	72	76	80	Switch[7]
CD4>=300 at week 48																Start STI			Restart ART			Start STI		
Adherence assessment and 4 weeks ART supply *		X		X	X	X	X	X	X	X	X	X	X	X	X	STI or X	STI or X	STI or X	X	X	X	STI or X	STI or X	X
Consent to screening and patient information sheet	X																							
Informed Consent		X																						X
History & Physical[1]	X	X	X	X	X	X			X	X		X			X	X		X			X			X
Symptom check list		X	X	X	X	X	X	X	X	X	X	X	X	X	X	X	X	X	X	X	X	X	X	X
Pregnancy Test[2]	X								X						X						X			
Haematology[3]	X			X		X			X			X			X			X			X			X
Biochemistry[4]	X			X		X			X			X			X			X			X			X
Lymphocyte Subsets [5]	X					X			X			X			X			X			X			X
Plasma storage[6]	X	X		X		X			X			X			X			X			X			X

* **Patients will return 4-weekly to see the nurse or doctor, return used drug containers and receive 4 weeks of ART (except for STI).** Nurse should check if patient has any new symptoms since the last visit. If yes, patient should see the trial doctor BEFORE PROCEEDING with structured treatment interruption (STI). The doctor will prescribe antiretroviral therapy and make decisions on any modifications of therapy as necessary.

1 Clinical: including weight, WHO staging for HIV.

2 At screening, and subsequently at start of STI in those randomised to STI; see section 7.3.4 if positive.

3 Haematology: Hb, MCV, WBC, Lymphocytes, Neutrophils, and Platelets.

4 Biochemistry: Urea, Creatinine, AST or ALT, Bilirubin

5 CD4, CD8, CD3 percentage and absolute, total lymphocyte count

6 Take 6 ml EDTA blood. Store DNA pellet at week –2, 0, 24, 48 and then every 48 weeks (if feasible). See Appendix 7.0 for instructions about storage. Take plasma (DNA pellet if feasible) at time of switch. Take plasma as near as possible to time of delivery for any women giving birth in DART

7 At time of switch to 2nd line reset flow sheet to visit week 0.

Table 2 LCM group (+/- STI co-enrolment): weeks 84-180

EVENTS	WEEK IN TRIAL																									
Doctor/Nurse visit * ■<br>Nurse visit □	84	88	92	96	100	104	108	112	116	120	124	128	132	136	140	144	148	152	156	160	164	168	172	176	180	12 weekly	Switch[7]
CD4>=300 at week 48		Restart ART			Start STI			Restart ART			Start STI			Restart ART			Start STI			Restart ART			Start STI				
Adherence assessment and 4 weeks ART supply*	STI or X	X	X	X	STI or X	STI or X	STI or X	X	X	X	STI or X	STI or X	STI or X	X	X	X	STI or X	STI or X	STI or X	X	X	X	STI or X	STI or X	STI or X		X
Informed Consent																											X
History & Physical[1]	X			X			X			X			X			X			X			X			X	X	X
Symptom check list	X	X	X	X	X	X	X	X	X	X	X	X	X	X	X	X	X	X	X	X	X	X	X	X	X	X	X
Pregnancy Test[2]				X						X						X						X					
Haematology[3]	X			X			X			X			X			X			X			X			X	X	X
Biochemistry[4]	X			X			X			X			X			X			X			X			X	X	X
Lymphocyte Subsets [5]	X			X			X			X			X			X			X			X			X	X	X
Plasma storage[6]	X			X			X			X			X			X			X			X			X	X	X

* **Patients will return 4-weekly to see the nurse or doctor, return used drug containers and receive 4 weeks of ART (except for STI).**

The doctor will prescribe antiretroviral therapy and make decisions on any modifications of therapy as necessary.

1 Clinical: including weight, WHO staging for HIV

2 At screening, and subsequently at start of STI in those randomised to STI; see section 7.3.4 if positive.

3 Haematology: Hb, MCV, WBC, Lymphocytes, Neutrophils, and Platelets

4 Biochemistry: Urea, Creatinine, AST or ALT, Bilirubin

5 CD4, CD8, CD3 percentage and absolute, total lymphocyte count

6 Take 6 ml EDTA blood. Store DNA pellet at week 96 and then every 48 weeks (if feasible). See Appendix 7.0 for instructions about storage. Take plasma (DNA pellet if feasible) at time of switch. Take plasma as near as possible to time of delivery for any women giving birth in DART

7 At time of switch to 2nd line reset flow sheet to visit week 0.

Table 3 Clinical Monitoring Only (CMO) Group (+/- STI co-enrolment)

EVENTS	WEEK IN TRIAL																						
Doctor/Nurse visit* (light grey)<br>Nurse visit (white)<br>Doctor only visit* (dark grey)	Screening Week-2	Start therapy Week 0	2	8	12	16	20	24	28	32	36	40	44	48	52	56	60	64	68	72	76	80	Switch[7]
CD4>=300 at week 48															Start STI			Restart ART			Start STI		
Adherence assessment and 4 weeks drug supply*		X		X	X	X	X	X	X	X	X	X	X	X	STI or X	STI or X	STI or X	X	X	X	STI or X	STI OR X	X
Consent for screening and patient information	X																						
Informed consent		X																					X
History & Physical[1]	X	X	X	X	X			X	X		X			X	X		X			X			X
Symptom check list		X	X	X	X	X	X	X	X	X	X	X	X	X	X	X	X	X	X	X	X	X	X
Pregnancy Test[2]	X							X						X						X			
Haematology[3]	X				X			X			X			X			X			X			X
Biochemistry[4]	X				X			X			X			X			X			X			X
Lymphocyte Subsets – results returned to clinician [5a]	X													X									
Lymphocyte Subsets –results not returned to clinician[5b]					X			X			X						X			X			X
Plasma storage	X	X			X			X			X			X			X			X			X

* Patients will return 4-weekly to see the nurse or doctor, return used drug containers and receive 4 weeks of ART (except for STI). **Nurse should check if patient has any new symptoms since the last visit, if yes patient should see the trial doctor BEFORE PROCEEDING with structured treatment interruption (STI)** The doctor will prescribe antiretroviral therapy and make decisions on any modifications of therapy as necessary.

1 Clinical: including weight, WHO staging for HIV.

2 At screening, and subsequently at start of STI in those randomised to STI; see section 7.3.4 if positive

3 **Blinded** Haematology: Hb, MCV, WBC, Lymphocytes, Neutrophils, Platelets, except at screening (week –2) when results returned to clinician.

4 **Blinded** Biochemistry: Urea or Creatinine, AST or ALT, Bilirubin, except at screening (week –2) when results returned to clinician

5a Return results at baseline. At week 48 return result as </>=300 cells/mm^3. If result at week 48 is >=300 cells/mm^3, randomise to STI or no STI . A result taken at time of switch to second-line therapy should not be made available to clinician. After switch, all patients receive at least 48 weeks of continuous ART. At the next scheduled visit where lymphocyte subsets are taken prior to a scheduled STI but at least 48 weeks post switch, CD4 cell count will again be made available to the clinician (as </>=300 cells/mm^3). If this result is >=300 cells/mm^3, patients in the STI arm may recommence STI. If CD4 count is <300 cells/mm^3, then patient continues ART with no further STI.

5b **Blinded** CD3, CD4, CD8 percentage and absolute, total lymphocyte count.

6 Take 6 ml EDTA blood. Store DNA pellet at week –2, 0, 24, 48 and then every 48 weeks (if feasible). See Appendix 7.0 for instructions about storage. Take plasma at time of switch (and DNA pellet if feasible). Take plasma as near as possible to time of delivery for any women giving birth in DART

7 At time of switch to 2nd line reset flow sheet to visit week 0.

Table 4 CMO Group (+/- STI co-enrolment): weeks 84 - 180

EVENTS	WEEK IN TRIAL																									
Doctor/Nurse visit * ▩<br>Nurse visit ☐	84	88	92	96	100	104	108	112	116	120	124	128	132	136	140	144	148	152	156	160	164	168	172	176	180	12 Weekly	Switch[7]
CD4>=200 at week 48		Restart ART			Start STI			Restart ART			Start STI			Restart ART			Start STI			Restart ART			Start STI				
Adherence assessment and 4 weeks drug supply*	STI or X	X	X	X	STI or X	STI or X	STI or X	X	X	X	STI or X	STI or X	STI or X	X	X	X	STI or X	STI or X	STI or X	X	X	X	STI or X	STI or X	STI or X		X
Informed Consent																											X
History & Physical[1]	X			X			X			X			X			X			X			X			X	X	X
Symptom check list	X	X	X	X	X	X	X	X	X	X	X	X	X	X	X	X	X	X	X	X	X	X	X	X	X	X	X
Pregnancy Test[2]				X						X						X						X					
Haematology[3]	X			X			X			X			X			X			X			X			X	X	X
Biochemistry[4]	X			X			X			X			X			X			X			X			X	X	X
Lymphocyte Subsets- return results to clinician[5a]																											
Lymphocyte Subsets- do not give results to clinician[5b]	X			X			X			X			X			X			X			X			X	X	X
Plasma storage[6]	X			X			X			X			X			X			X			X			X	X	X

* **Patients will return 4-weekly to see the nurse or doctor return used drug containers and receive 4 weeks of ART (except for STI).** The doctor will prescribe antiretroviral therapy and make decisions on any modifications of therapy as necessary.

1 Clinical: including weight, WHO staging for HIV.

2 At screening, and subsequently at start of STI in those randomised to STI; see section 7.3.4 if positive

3 **Blinded** Haematology: Hb, MCV, WBC, Lymphocytes, Neutrophils, Platelets, except at screening (week –2) when results returned to clinician

4 **Blinded** Biochemistry: Urea or Creatinine, AST or ALT, Bilirubin, except at screening (week –2) when results returned to clinician

5a Return results at baseline. At week 48 return result as </>=300 cells/mm^3. If result at week 48 is >=300 cells/mm^3, randomise to STI or no STI. A result taken at time of switch to second-line therapy should not be made available to clinician. After switch, all patients receive at least 48 weeks of continuous ART. At the next scheduled visit where lymphocyte subsets are taken prior to a scheduled STI but at least 48 weeks post switch, CD4 cell count will again be made available to the clinician (as </>=300 cells/mm^3). If this result is >=300 cells/mm^3, patients in the STI arm may recommence STI. If CD4 count is <300 cells/mm^3, then patient continues ART with no further STI.

5b **Blinded** CD3, CD4, CD8 percentage and absolute, total lymphocyte count. .

6 Take 6 ml EDTA blood Store DNA pellet at week –2, 0, 24, 48 and then every 48 weeks (if feasible). See Appendix 7.0 for instructions about storage. Take plasma at time of switch and DNA pellet if feasible. Take plasma as near as possible to time of delivery for any women giving birth in DART

7 At time of switch to 2nd line reset flow sheet to visit week 0.

2.0 Background and Rationale

2.1 Introduction

The prevalence of HIV infection is very high (>20%) and still rising in many Sub-Saharan African countries. Prevention messages in the absence of treatment appear to have limited impact and the effect of the epidemic on the social and physical well-being of individuals and on the economy of these countries is devastating. Combination antiretroviral therapy (ART) has had a dramatic effect on the morbidity and mortality of HIV-infected adults and children in well-resourced countries. It may also reduce transmission of HIV, through reduction in HIV viral load. Until recently very high costs were an absolute barrier to the introduction of ART on a wide scale in resource-poor countries. However, these have recently been reduced substantially, making the provision of ART to HIV-infected persons in Africa a realistic possibility. However, the costs and infrastructure required for monitoring patients taking these drugs for efficacy and toxicity, as carried out in developed countries, are also extremely high. There are concerns about the lack of infrastructure to administer ART and to sustain adherence and monitor toxicity in developing country settings. Trials are needed to evaluate strategies for using ART, which are tailored to the circumstances of developing countries.

DART aims to address two major questions. The first is whether ART can be safely given with clinical monitoring alone, in the absence of regular viral load and CD4 measurements and laboratory monitoring for toxicity. The second question concerns the use of pulse compared with continuous ART, the hypothesis being that pulse therapy would reduce the cost and toxicity of ART and may improve adherence, without increasing the risk of developing resistance or disease progression.

2.2 Monitoring for efficacy and toxicity

In persons starting ART in well-resourced countries, monitoring of prognostic markers of HIV disease (HIV RNA viral load and CD4 cell counts) is routinely undertaken in order to make decisions about starting and switching therapy. In addition, routine monitoring of laboratory markers is undertaken in order to detect bone marrow, liver and other toxicities to ART.

Although trials have been carried out, and others are currently ongoing, to ascertain the value of resistance testing in the management of HIV, no trials have been undertaken in developed countries to determine the efficacy and cost-effectiveness or the optimum frequency of HIV RNA viral load or CD4 count monitoring. There have been no studies evaluating the need for, or frequency of laboratory tests required for monitoring toxicity of ART. HIV RNA and CD4 count tests are particularly expensive (although the cost may come down), and unavailable outside major centres in Africa. In addition there are important issues of quality control. Requirements for monitoring schedules similar to those in developed countries are likely to be a barrier to the administration of ART to large numbers of persons in the African setting. Although HIV RNA viral load is an important prognostic marker and is widely used to monitor therapy when first introduced, recently greater emphasis has been placed on monitoring CD4 cell counts as these are better predictors of the immediate risk of clinical disease progression and mortality, particularly in late disease. Furthermore, with the current therapeutic options, it is difficult to maintain HIV RNA below the limit of detection of current assays, over a long period of time, although CD4 count increases may be maintained.

Many would therefore agree that HIV-RNA viral load monitoring is unlikely to be cost-effective or necessary in the African setting. However, it is unclear whether decisions about management of ART toxicity or switching ART can be made on the basis of clinical signs and symptoms alone, or should be on the basis of clinical monitoring plus regular laboratory monitoring for toxicity and CD4 cell counts.

2.3 Structured Treatment Interruptions (STIs)

Structured Treatment Interruptions (STIs), whereby periods on ART are followed by periods off treatment, are being explored in a number of circumstances in developed countries. First, following recent infection with HIV, there is some evidence from small uncontrolled studies that STIs lead to control of viral replication even during periods of no therapy in some patients [1-4]. Second, in patients who have failed several ART regimens and have resistant virus, there is some evidence that an STI leads to the re-emergence of wild-type sensitive virus which may result in a better response to a subsequent new ART regimen [5, 6]; this is being explored in ongoing randomised trials (e.g. OPTIMA Trial). Finally, in chronically infected patients, the possibility of reducing toxicity, improving adherence and reducing costs, while maintaining the clinical and immunological well-being of patients has been reported in observational studies of STI [7,8].

Different periods of interruption of ART are being explored in a number of trials of pulse compared with continuous ART in patients with suppressed viral replication in developed countries. One approach is to cycle on and off ART rapidly (7-days on, 7-days off). This has been reported in small studies to prevent an increase in viral load and the re-emergence of resistance, but may be difficult to implement. Other approaches being evaluated include longer cycles (e.g. 1, 2, 3 or 4 months on and off therapy), and trials where the length of treatment interruption is individualised and driven by the rate of fall in CD4 cell count after stopping ART (e.g. TILT Trial). To date, no evidence is available to define the most appropriate approach. This may anyway be different in the African setting, where many patients are likely to start ART with much lower CD4 cell counts than in developed countries and where CD4 count monitoring is expensive and not readily available. The limited data available from STI studies suggest that the average fall in CD4 count is about 20 cells a month, but may be faster, up to 50-60 cells/month [9], in patients starting ART with a very low CD4 count.

STI trials in developed countries aim to keep CD4 cell counts above a threshold of 200 cells/mm^3. However, most opportunistic infections occur when the CD4 count falls below 50 cells/mm^3. It would be unreasonable not to start ART in patients in most need in Africa, nearly all of whom will have symptomatic disease and many of whom will have CD4 counts below 50 cells/mm^3. It could be argued that it would not be appropriate to interrupt ART until CD4 has increased to above 200 cells/mm^3.

2.3.1 STI randomisation termination

On 15th March 2006 following the recommendation of the DSMC the STI randomisation was terminated early. All patients were transferred to continuous therapy.

2.4 Antiretroviral Therapy Regimens

The role of regular CD4 count monitoring could not be evaluated if the trial did not incorporate a second line ART regimen. The choice of first-line therapy is in line with recently developed WHO guidelines on the use of antiretroviral therapy in resource-poor settings [10]. These recommend use of a double NRTI plus an NNRTI or a triple NRTI regimen, preserving PI-containing regimens for a second-line regimen. The key questions being addressed in the DART trial do not necessitate the use of the same first-line regimen for all patients in the trial. Further, including more than one first-line regimen would enhance the generalisability of results.

2NRTI + NNRTI Regimens

Possible options for this first-line regimen include combivir (zidovudine and lamivudine, CBV plus an NNRTI; e.g. nevirapine (NVP) or efavirenz (EFZ). Both combinations have proven first line efficacy. EFZ may be teratogenic if used in pregnancy, which would be a major issue for women of childbearing age. NVP is being used increasingly as a single dose in women during labour (and one dose to the baby at birth) to reduce mother-to-child HIV transmission (MTCT) and it is unclear to what extent the widespread use of NVP for treatment could impact MTCT programs. Further, if women have already received NVP during a previous pregnancy, it is possible (although unproven) that its subsequent efficacy as part of a treatment regimen may be reduced, as it is known that resistance can develop rapidly. Both NVP and EFZ have long half-lives and resistance develops rapidly. Stopping the NNRTI some days before the NRTIs during treatment interruptions therefore needs to be considered. It is planned to obtain additional pharmacokinetic data to facilitate best management of this issue.

Triple NRTI Regimens

The formulation of a triple NRTI combination of zidovudine, lamivudine and abacavir (Trizivir, TZV), given as one tablet twice a day, provides the possibility of a well-tolerated, simple regimen with the advantage that there is a reduced possibility of developing resistance (as all drugs are in one tablet) and there are likely to be fewer interactions with other drugs (e.g. anti-tuberculosis drugs) than with other anti-HIV drugs, particularly protease inhibitors. However, TZV has potential hazards related to abacavir (ABC), which is associated with hypersensitivity in about 3% of patients. This can be fatal if the patient is re-challenged after stopping the drug for symptoms of hypersensitivity. There is no evidence that interruption of therapy not preceded by hypersensitivity symptoms leads to problems but there are relatively few data on this. Any patient stoppingABC for hypersensitivity would need to receive a replacement drug and would then have to take therapy as separate formulations (two formulations if using CBV or three if all three drugs are provided separately). Giving ABC as part of a regimen with STIs will require close monitoring, particularly in the early phase of treatment. However, the symptoms of hypersensitivity to ABC are predominantly clinical (fever and rash) and careful monitoring will ensure safety of patients.

Information on whether symptoms of immune reactivation post ART or symptoms of tropical diseases could be mistaken for the toxicity of ABC, when used in combination with CBV or as TZV, will be valuable in terms of the widespread use of this combination in developing countries and will be obtained from the NORA substudy in the DART trial (see Appendix 2.0, page 52).

Other possible triple NRTI regimens include CBV plus didanosine (ddI) or CBV plus a nucleotide reverse transcriptase inhibitor, tenofovir (TDF). TDF is available as a single tablet to be taken once daily. It has an excellent safety profile, and has recently been shown to have efficacy at least similar to NRTIs in a 48-week trial comparing the effect on HIV RNA of D4T+3TC+EFV with that of TDF+3TC+EFV [11]. In addition, HIV resistance against TDF develops very slowly, making it an attractive drug to use in resource-poor settings in the absence of HIV RNA resistance testing.

Second line ART

Following the WHO public health approach to ART in resource limited settings, patients in DART continue on first-line ART to clinical/immunological failure rather than switching to second-line at virological failure, as in industrialised countries. There is considerable interest in determining the most appropriate second-line strategies for such patients who may have acquired significant resistance whilst continuing first-line treatment to clinical/immunological failure. There are currently no robust data on the optimal treatment strategy for such patients, because clinical practice in industrialised countries is to switch patients earlier for virological failure based on frequent, routine viral load tests which are currently both technically complex and expensive. (Of note, there are no data comparing switch at virological versus clinical/immunological failure to determine the long-term impact in any setting.)

Further, the number of patients who have taken the most commonly used DART regimen of CBV plus TDF (3 NRTI drugs) world-wide is small, and so there are even fewer data to inform possible choice of second-line therapies for the majority of the DART population. Triple NRTI regimens such as CBV plus TDF are attractive for resource limited settings because of their low pill burden, low frequency of adverse reactions and compatibility with other drugs including anti-tuberculosis therapy, and are being viewed as increasingly important in the WHO Public Health approach.

The first 20 patients identified as failing clinically/immunologically on CBV plus TDF in DART were switched to a second line regimen (bPI+NNRTI+-NRTI) based on clinical opinion at the time. Genotyping was carried out on these patients and revealed extensive nucleoside/nucleotide resistance mutations. This is not unexpected as clinical/immunological failure is likely to occur after many months of viral replication, with accumulation of resistance mutations. It should be noted however that these early failures may not be representative and could have had baseline resistance from undisclosed ART before entry to the DART trial. As yet there are no genotype data from patients failing on 2NRTI plus NNRTI first line in DART, but from other studies in resource-limited settings it is likely that similar extensive NRTI mutations will be seen as well as key NNRTI mutations (rendering this class ineffective).

Therefore, in many patients with clinical or immunological failure of first-line therapy, there may be at best limited activity from any NRTIs included in second line. Expert opinion agrees that a boosted PI (bPI) based regimen will be the most effective second line, but the value of adding NRTIs or NNRTIs is less clear. The use of a new currently licensed NNRTI following failure of a first line regimen containing an NNRTI is unlikely to be beneficial as there is cross-resistance among all currently licensed NNRTIs (which also have a low genetic barrier to resistance). However, two drug, two class, NNRTI plus PI regimens have been used successfully in NNRTI naïve patients (the BIKS study [12]). Non-inclusion of an NRTI in second line could preserve any residual activity for a third line/salvage regimen and would reduce the pill burden and possibly the toxicity from the second line regimen. Of note,

changing to a bPI as part of a regimen is a substantial increment in regimen complexity and tolerability compared to the first line regimens used in DART.

Another question about second-line ART is whether potency of either a bPI or bPI plus NNRTI (for those that have not failed on a NNRTI containing regimen) could be augmented by the addition of new NRTIs, or alternatively by continuing one or more of the NRTI(s) used in first-line therapy, in order to maintain the presence of a highly resistant, but less fit virus with significantly reduced potency. Such viruses with large numbers of nucleoside associated mutations (NAMs) have been reported to have increased susceptibility to NNRTIs. Thus there are interesting questions around the effect of resistance on viral fitness and induction of NNRTI hypersusceptibility which could be addressed if one or more additional NRTI(s) were added to either a bPI or PI/NNRTI regimen. This concept could be particularly valuable for patients failing an NNRTI regimen, who are likely to have extensive NNRTI and NRTI resistance at clinical/immunological failure.

2.5 Rationale

The DART trial is addressing a very important question about safe management of patients taking ART in an environment where routine toxicity and efficacy tests may not be available in compliance with the principles of Good Clinical Practice and the Declaration of Helsinki.

In the African setting, many patients adopt a practice of interrupting therapy because of limited funds. However, this is usually done in a haphazard way and it is unclear whether it is safe. STI trials are being undertaken in chronic HIV infection in developed countries with the aims of reducing toxicity, costs, and possibly improving adherence, without compromising efficacy. In Africa reduction of costs, in particular, would have major advantages. However, as the natural history of HIV in Africa differs from that in developed country settings (e.g. a much higher proportion of TB) and patients are likely to start ART later in the course of HIV disease, it is necessary to evaluate the safety of STI in an African setting. The optimum period for STI is not yet known but rapid cycling of drugs would be too complex. A 3-month cycle has been chosen for DART but may be altered following data from the pilot phase (see Appendix 1.0, page 49).

ART administration in developed countries is accompanied by tests for monitoring ART efficacy and toxicity. However, no trials evaluating the need for laboratory monitoring over and above clinical monitoring alone have been undertaken in developed countries. The cost of tests to measure CD4 cell count, and also of haematology and liver function tests is a major barrier to administration of ART to large numbers of persons in resource-poor settings. Even if costs are reduced, these tests are unlikely to be available outside major centres. Therefore there is a need to explore the question of the necessity of these laboratory tests with the introduction of ART in Africa.

The advantages of combining both the STIs and monitoring questions into a three-centre trial are 3-fold: first, a larger trial can be undertaken than would be possible in a single clinical centre; second, the logistics of supplying drugs for a single trial to address both questions, rather than two separate trials, would be simpler and cheaper; thirdly, collaboration across sites in Africa would be likely to build capacity and enhance regional knowledge about the use of ART.

A simple ART regimen is important for use in the African setting. At a recent meeting of HIV experts at WHO to develop guidelines on use of ART in resource-poor countries, the combination of ZDV, 3TC and ABC in one preparation (trizivir) was considered as a front-

runner for first-line therapy because of ease of administration (one pill twice daily), and lack of interaction with other drugs, in particular anti-TB drugs [10]. However, although there is good data to guide the management of hypersensitivity reactions to ABC in developed countries, this may be different in the African setting because immune reconstitution syndromes may be more common after starting ART in patients with advanced disease living in areas with high prevalence of other infectious diseases. A 600-patient, double blind substudy comparing the toxicity of ABC and NVP (both given with combivir) will therefore be undertaken within the DART trial (see Appendix 2.0, page 52). (This will require placebos for both NVP and ABC.) For the remaining patients in the main study (2700 patients), combivir + TDF will be the first-line regimen for 2400 patients and combivir + NVP for 300 patients.

DART provides an important opportunity to address issues around second-line regimens. In view of the paucity of data on this issue in these settings, the aim would be to capitalise on the infrastructure of DART to conduct a feasibility study to explore the activity of a number of such regimens, using surrogate endpoints (CD4) to identify the most appropriate second-line regimens to take forward into a clinical endpoint trial to assess their **clinical** effectiveness, while minimising resistance and preserving options for third-line (salvage) regimens. All strategies considered will include a boosted PI, in combination with either an NNRTI (with or without a NRTI) or with 2 NRTIs.

3.0 Objectives

3.1 Main DART Trial Objectives

To compare, in terms of clinical HIV disease progression or death:

1. Clinical monitoring only (CMO) versus routine regular laboratory and clinical monitoring (LCM)
2. Structured Treatment Interruptions during first line ART (STIs: 12 weeks on, 12 weeks off therapy) versus continuous ART, initiated if the CD4 count has increased to above 300 cells/mm^3 (after 48 weeks on ART).

The hypothesis is that CMO will result in similar outcomes to LCM, and that ART administered as pulse therapy (STI) will result in similar outcomes to continuous ART, in terms of progression of clinical HIV disease or death.

3.2 STI Pilot Study Objectives

The initial non-randomised pilot study of STIs will inform on the safety of the 12 weeks on, 12 weeks off STI strategy (see Appendix 1.0, page 49) and only after the completion of this substudy will the second randomisation commence.

3.3 Abacavir Safety Substudy Nevirapine OR Abacavir (NORA) Objectives

This randomised sub-study of 600 patients will address issues of safe administration of abacavir (ABC) in resource poor settings and will compare the safety of ABC with that of NVP when used in combination with combivir (see Appendix 2.0, page 52).

3.4 Second line feasibility study objectives

This randomised feasibility study of patients failing first-line regimens of DART will provide preliminary, surrogate endpoint (CD4) data to select the most promising second-line regimens/strategies to be evaluated in a large-scale clinical endpoint trial. Specifically, the feasibility study aims to compare, in terms of CD4 response

1. Strategies of using bPI+NNRTI or all three classes following clinical/immunological failure of regimens not containing an NNRTI
2. Strategies of using bPI plus either new NRTIs (potential residual activity despite possible cross-resistance) or recycled NRTIs (to maintain drug pressure and presence of variants with lower fitness) following clinical/immunological failure of NNRTI containing regimens.

4.0 Endpoints

4.1 Primary Endpoints (CMO/LCM and STI/CT):

- *Efficacy*: Progression to a new WHO stage 4 HIV event or death (see Appendix 5.0, page 76).
- *Safety*: Any serious adverse event, which is not HIV related (see section 7.9, page 38 and 0, page 70).

4.2 Secondary Endpoints (CMO/LCM and STI/CT):

- Progression to a new or recurrent WHO stage 4 HIV event or death
- Progression to a new WHO stage 4 HIV event or death from 6 weeks after randomisation
- Progression to a new or recurrent WHO stage 4 HIV event or death from 6 weeks after randomisation
- Any grade 3 or 4 adverse events (see 0, page 70)
- Number and class of anti-HIV drugs received by 3 years
- Time to cessation of first-line regimen for failure
- Adherence as measured by questionnaire and pill counts
- CD4 count at 3 years (provided that it is at least 2 months after restarting ART for those in the STI group)
- HIV RNA viral load (performed retrospectively) at 3 years (providing that it is at least 2 months after restarting ART for those in the STI group)
- HIV resistance profiles at 3 years in those with detectable viral load (providing that it is at least 2 months after restarting ART for those in the STI group)

4.3 Primary Endpoints (second-line randomisation feasibility study):

- *Efficacy*: change in CD4 count from time of switch to second-line to 24 weeks.
- *Safety*: Any serious adverse event, which is not HIV related (see section 7.9, page 38 and 0, page 70).

4.4 Secondary Endpoints (second-line randomisation feasibility study):

- Progression to a new or recurrent WHO stage 4 HIV event or death
- Progression to a new or recurrent WHO stage 3 or 4 HIV event or death
- Change in CD4 count from time of switch to second-line to 48, 72 and 96 weeks
- Any grade 3 or 4 adverse events (see 0, page 70)
- HIV RNA viral load (performed retrospectively) at 48, 72 and 96 weeks
- Adherence as measured by questionnaire and pill counts
- Health economic outcomes

5.0 Design

5.1 Type of design

DART is an international, three-centre open-label randomised trial in symptomatic HIV infected adults in Africa. There will be two randomisations and the total duration of the trial will be 6 years.

5.1.1 Randomisation to CMO or LCM arm

3300 patients will be randomised to CMO or LCM over a period of -two years. Randomisation will be stratified by CD4 count (0-99, 100-199) and by clinical sites.

5.1.2 STI Pilot

Because there are no data on STI in the African setting, where patients are likely to have low CD4 cell counts before starting ART, a non-randomised pilot study of the first 100 patients eligible for the STI randomisation will be undertaken (see Appendix 1.0, page 49). This would be expected to delay the onset of the second randomisation by a minimum of 6 months (the time taken to enrol patients (estimated to be 2-3 months), and at least 3 months follow-up). During this 24-week period the patients will be seen by the doctor every 4 weeks and 4-weekly CD4 cell counts will be performed on all 100 patients and reported to the clinicians in the LCM arm and to the subgroup of DSMC in the CMO arm

5.1.3 STIs or continuous ART

Of the 3300 patients randomised to CMO or LCM, we would expect at least 600 to be eligible for the second randomisation (STIs versus continuous ART) commencing after the STI pilot (see section 5.1.2 above and Appendix 1.0, page 49), which should be completed by

about 12 months after the start of the trial. Randomisation to STIs or continuous ART would be completed by the end of year 3.

5.1.4 Feasibility randomisation for second line ART for those failing first line ART

As data on optimal treatment of patients failing clinically and/or immunologically on a first-line regimen do not exist, all patients switching to second-line therapy in DART will be invited to consent to be randomised within a feasibility study using surrogate (CD4) endpoints to identify the most appropriate strategies to take forward into a clinical endpoint trial.

Patients failing first-line ART in DART and switching to second-line, who are eligible and consent, will be randomised to one of the following arms:

Strata 1: failure on an NNRTI containing regimen*

(A) bPI + ABC + ddI

(B) bPI + TDF + CBV†

Strata 2: failure on a regimen not containing an NNRTI

(C) bPI + NNRTI

(D) bPI + NNRTI + 3TC

(E) bPI + NNRTI + ddI

*Note: any patient who did not disclose prior NNRTI experience before entering DART & is retrospectively considered to have failed immunologically and/or clinically whilst taking NNRTI should be enrolled in strata 1

† patients with previous adverse reaction to ZDV may receive bPI+TDF+3TC only

Patients failing first line ART will be randomised 1:1 across arms A and B in strata 1 and 1:1:1 across arms C, D and E in strata 2. The decision of whether a patient has failed on an NNRTI (likely to have led to resistance) will be left up to the treating clinician. For example a patient failing immunologically/clinically whose only NNRTI experience is single dose NVP for PMTCT should go into strata 2.

As currently available NNRTIs (NVP, EFZ) are not active against NNRTI-resistant strains, and as NNRTIs have a low genetic barrier to resistance, the majority of patients failing NNRTI-containing regimens will have NNRTI-resistant virus. Therefore such patients in DART will be randomised between A and B only (Strata 1). For most DART patients that have failed an NNRTI regimen the first line NRTIs will have been ZDV and 3TC, the NRTIs with most residual activity are likely to be ABC, ddI and TDF (note that co-treatment with ddI and TDF is contraindicated) – arm A. However, rather than maximising residual activity an alternative strategy is to maintain virus with lower fitness due to extensive TAMs and M184V – arm B.

In contrast NNRTIs are likely to be highly active in patients who have failed on a triple NRTI first line regimen: therefore bPI+NNRTI will be the backbone for all such patients (Strata 2). Patients initiating second line with bPI+NNRTI will not have used ddI in DART. Although there is likely to be some resistance to this drug arising from cross-resistance to the first line NRTIs it is not known whether adding ddI to a 2-drug, 2-class regimen improves outcome or merely increases regimen complexity – arm E. An alternative strategy is to maintain virus with lower fitness due M184V by keeping 3TC in the regimen – arm D. A third strategy is

not to use an NRTI, to reduce pill-burden and retain any residual activity for salvage therapy – arm C.

In view of contraindications for EFZ in pregnancy, NVP should be used as the NNRTI wherever possible for women of childbearing age.

5.2 Antiretroviral Therapy

See section 8.0, page 39 and Appendix 3.0, page 66 for full details. First and second-line regimens will be available for all patients as well as the option of changing individual drugs (within the same class) for toxicity (see sections 7.6, page 37 and 8.0, page 39, Appendix 3.0, page 66 and 0, page 70). For patients in whom second-line therapy fails, the most appropriate combination of available drugs will be offered (see section 8.0, page 39). Every effort will be made to make further ART available after the trial but it is agreed that local health authorities will commit to providing adequate care for the participating patients (see Appendix 9.0, page 90).

6.0 Patient Population

6.1 Inclusion Criteria (CMO/LCM)

1. Documentation of HIV-1 infection: antibody positive serology by ELISA test (confirmed by licensed second ELISA or Western Blot).
2. Age $\geq$ 18 years
3. Symptomatic WHO stage 2, 3 or 4 HIV disease ***and*** CD4 < 200 cells/mm^3
4. ART naïve (except for ART use during pregnancy for the prevention of mother-to-child HIV transmission).
5. Agreement and documented informed consent to be randomised to CMO or LCM and to STI or continuous ART, if eligible.
6. Life expectancy of at least 3 months.

6.2 Exclusion criteria (CMO/LCM)

1. Cannot, or unlikely to attend regularly (e.g. usual residence too far from Study Centre)
2. Likelihood of poor compliance
3. Presence of acute infection (e.g. malaria, acute hepatitis, pneumococcal pneumonia, non-typhoid salmonella septicaemia, cryptococcal meningitis). Patients may be admitted after recovery of an acute infection. Patients with tuberculosis (TB) will not be enrolled while on the intensive phase of anti-tuberculosis therapy, but should be re-evaluated after the intensive phase and a decision made then about starting ART. Patients starting ART whilst on anti-tuberculosis therapy after the intensive phase will not receive NVP, nor will they be randomised into the NORA substudy.
4. On chemotherapy for malignancy
5. Laboratory abnormalities which are a contra-indication for the patient to start ART (e.g. Haemoglobin <8g/dl, neutrophils <0.50x10^9/l, AST or ALT >5 x the upper limit

of normal (ULN), grade 3 renal dysfunction – creatinine >360 µmol/l and/or urea >5 x ULN).

6. Pregnancy or breast-feeding

6.3 Number and source of subjects (CMO/LCM and STI/CT)

Patients will be recruited from three sites: PPD Uganda, PPD Uganda; and PPD Zimbabwe. It is expected that approximately 1000 patients will be recruited from each site, plus 300 from a satellite clinic in PPD The STI pilot (see Appendix 1.0, page 49) will be conducted in all three sites. The NORA substudy (see Appendix 2.0, page 52) will be conducted in PPD and PPD

This trial requires that patients commit to long-term follow-up. Therefore priority will be given to those who can regularly attend for follow-up. Patients will be recruited from 3 sources:

1. patients who have been tested for HIV infection in the past and are in regular follow-up;
2. following ***recovery*** from a hospital admission during which HIV infection has been diagnosed, and followed by attendance at a follow-up outpatient clinic (N.B. no patient should be recruited during a hospital admission);
3. from outpatient clinics (e.g. general medical, TB clinics). For newly HIV diagnosed patients, the HIV counselling and testing process will be fully carried out.

To avoid inequity in access to the trial, the recruitment will be carried out as eligible patients visit the outpatient clinics to consult over a recent health problem or who come for a previously scheduled follow up visit.

6.4 Number and source of subjects (second-line feasibility study randomisation)

All patients failing first-line therapy in DART and switching to second-line will be eligible. Although additional informed consent will be sought for this randomisation, it is anticipated that refusal rates will be low, as the alternative is for the clinician to choose between the randomised arms based on no data.

The sample size available for this feasibility study is therefore constrained by the fact that only patients failing first-line therapy in DART and switching to second-line therapy will be enrolled. Currently the failure rate is approximately 5% per annum after the first year on first-line therapy. Assuming this rate increases at 2% per annum until the end of the trial, we estimate that 240 patients will switch to second-line therapy during 2007 (and will have 48 weeks follow-up by the end of 2008), although the exact numbers and split between NNRTI and NRTI failures are unknown – and could increase dramatically if the failure rate increased.

6.5 Co-enrolment in other studies

DART participants may co-enrol in any other study provided this does not compromise the integrity of the DART trial. Specifically the restrictions relating to laboratory monitoring must be maintained.

7.0 Procedures and Management of Subjects

7.1 Screening Procedure for CMO/LCM

At screening, HIV infected adults will be given an information sheet about the DART trial and asked to give consent to screening (see Appendix 6.0, page 77). Interested patients will have clinical information including medical history, examination, confirmation of WHO stage 2, 3, or 4, and weight recorded, and T cell subsets (CD4, CD8, CD3 and total lymphocyte count), haematology, biochemistry and pregnancy tests performed. Plasma and DNA pellets will be saved. Women of reproductive age will be given information about the risks of pregnancy in the trial and encouraged to avoid pregnancy.

7.2 Baseline procedure (Week 0 for CMO/LCM)

The time between screening and randomisation should preferably be within 2 weeks and not exceed 4 weeks.

Patients eligibility for enrolment will be confirmed. Patients with CD4 ≥200 cells/mm^3 can be enrolled later if the CD4 count falls to <200 cells/mm^3 and they are still in follow up. Those in whom the CD4 count is <200 cells/mm^3, with no haematological or biochemistry contraindications to starting ART, will be eligible for the trial.

Fully informed signed consent will be obtained just before randomisation (see Appendix 6.0, page 77). The patient must consent to both randomisations and agree to participate in the STI pilot if required (see Appendix 1.0, page 49). Consent forms should be kept securely at the clinical site and be available for monitoring.

When possible, and particularly if there is doubt about the place of residence of a patient, a study nurse/field worker, preferably the same nurse who gave information about the trial to the patient, will accompany the patient home and draw a map indicating the place of residence.

A trial register should be kept at the clinical site and will record all patients who are eligible and invited to join the trial. Those accepting will have name, date of birth (DOB), date of randomisation and trial number recorded. Those who refuse will have name, DOB, and reason for refusal recorded. The register will be kept in a secure place in each clinical site and will be the responsibility of the trial investigator at that site.

7.3 Randomisation and Enrolment

7.3.1 Randomisation List

A randomisation list for CMO/LCM will be prepared, by computer staff at MRC CTU under the direction of the trial statistician. Patient randomisation numbers will be prepared

separately for each centre and further stratified within centre by baseline CD4 cell count (0-99, 100-199). A single member of staff at each site who is not directly involved in patient care will be responsible for carrying out the randomisation process using a secure electronic system. A reliable back-up system will also be available. Randomisation will not take place until after the patient has given informed consent and is ready to receive therapy.

A randomisation list will be similarly prepared for STI/continuous ART after completion of the STI pilot. This will be stratified by centre, by week number at entry (52 or 76, see section 7.3.3 below), and by first randomisation to CMO/LCM.

Another randomisation list will be prepared for the second line feasibility study randomisation; this will be stratified by centre and CMO/LCM.

7.3.2 First Randomisation to CMO or LCM

The first randomisation is to CMO or LCM. The patient's eligibility for enrolment will be reviewed by the doctor at baseline (week 0, see Flow Sheets, section 1.3, page 16), having completed the screening CRF, including all laboratory results. Patients should start ART and be told their allocation on the day of randomisation. Those allocated to LCM will have CD4 cell counts undertaken 12-weekly and haematology and biochemistry tests undertaken at 4, and 12 weeks, and 12-weekly thereafter (see Flow Sheets, section 1.3, page 16).

7.3.3 Second Randomisation – STIs or continuous ART

The second randomisation **will not commence** until the pilot STI study has been completed (see Appendix 1.0, page 49). To determine whether a patient is eligible for the second randomisation, CD4 counts will be undertaken in all patients irrespective of the monitoring arm, at 48 weeks (or at 72 weeks if the patient was not randomised at week 52) and the results will be returned to the clinician. For patients allocated to CMO, these results will be reported as CD4<300 or ≥300 cells/mm^3. Eligibility for the second randomisation will depend on the CD4 cell count being 300 cells/mm^3 or higher. If the CD4 count is <300 cell/mm^3 at 48 weeks (72 weeks if the patient was not randomised at week 52), the patient will **not be eligible** for the second randomisation, but will continue to be followed for the evaluation of the monitoring strategies (see Trial Schema, section 1.2, page 14).

As the results of the STI pilot study have been reviewed by the Trial Steering Committee and Data and Safety Monitoring Committee (DSMC), and STI is still deemed to be safe (see Appendix 1.0, page 49), subsequent patients who are clinically well with CD4 cell counts of 300 cells/mm^3 or higher at 48 weeks (72 weeks if the patient is was not randomised at week 52) will be allocated in a 1:1 ratio to continuous ART or to ART with STI (12 weeks off followed by 12 weeks on ART, repeating the schedule until the end of the trial).

<u>Inclusion Criteria:</u>

1. CD4 count 4 weeks prior to STI randomisation visit ≥300 cells/mm^3

<u>Exclusion criteria:</u>

1. Pregnant or breast-feeding
2. WHO stage 3 or 4 illness in the 12 weeks prior to STI randomisation visit
3. Other illness at the STI randomisation visit such that the clinician believes it could be harmful to stop ART at this timepoint

4. Participation in the STI pilot study

Patients will return to the clinic at week 52 (or week 76 if the patient was not randomised at week 52), four weeks after the week 48 (or 72) CD4 cell count has been taken. Eligible patients will see the doctor at this visit (see Flow Sheets, section 1.3, page 16). They will be told that they are eligible to be included in the second randomisation, and will be randomised at that visit to the continuous ART arm or the STI arm. They will be requested to bring all unused medication with them, which will be taken from them if they are in the STI arm. If the doctor has clinical concerns about the patient at this visit, such that he/she feels that the STI should not commence despite the CD4 cell count being ≥300 cells/mm^3 four weeks previously, then he/she may defer randomisation and record using the appropriate form. In all patients undergoing the second randomisation and randomised to STI, this should commence immediately and should be within 5 weeks of the week 48 (or 72) CD4 cell count test (see Trial Schema, section 1.2, page 14 and Flow Sheets, section 1.3, page 16). NVP will be stopped seven days before combivir to avoid the risk of developing NVP resistance because of its long half-life. In practice NVP will be stopped on the day of commencement of STI and combivir alone continued for 7 more days.

The first 100 patients randomised to STI or CT will have plasma stored and CD4 cells measured 4 weekly, including at the time of randomisation; these counts will only be returned to clinicians for LCM patients. Two members of the TSC who are not involved with clinical management of DART patients will closely monitor individual CD4 counts and any WHO 3 and 4 events for these 100 patients on a monthly basis, **without** knowledge of CMO/LCM allocation. They could decide whether they need to inform the DSMC and TSC immediately, prompting a full analysis of all data accrued in the STI randomisation to this point in time for urgent consideration by the DSMC.

Note that the STI randomisation was terminated on 15th March 2006; all patients were offered continuous therapy.

7.3.4 Third randomisation – second line regimen feasibility study

Prior to randomisation to the second line feasibility study patients will go through counselling and information sessions and signed, informed consent will be obtained. A trial register for the second-line feasibility study should be kept at the clinical sites and will record all patients who are eligible (failing first-line therapy clinically/immunologically) and invited to join the feasibility study. Those accepting will have name, date of birth (DOB), date of randomisation in the second-line feasibility study and main DART trial number recorded. Those who refuse will have name, DOB, and reason for refusal recorded. The register will be kept in a secure place in each clinical site and will be the responsibility of the trial investigator at that site.

Randomisation to a second line regimen may be carried out at any clinic visit (scheduled or extra) beyond week 48 after enrolment in DART. Randomisation will be stratified by clinical site and CMO/LCM allocation.

Inclusion Criteria:

1. Completed 48 weeks in DART
2. Clinical and/or immunological assessment of failure whilst currently receiving first line regimen following section 7.6.2, page 37 and decision made that patient is to be switched to second-line

Exclusion criteria:

1. Continuing or starting treatment with any medications incompatible with any arm of the randomisation to which the patient could be assigned. [Note: it is recommended that patients remain on first-line therapy whilst tuberculosis treatment is received, but may be enrolled in the second line study if not taking rifampicin]

7.3.5 Pregnancy

Eligible women who are found to be pregnant at screening may be re-screened to ascertain eligibility to be enrolled in the trial after the baby is born and breast-feeding has finished, provided that enrolment is still open in that site.

7.3.6 Enrolment of family members into the trial

If more than one person from the same family is eligible and gives consent to enrolment in the trial, they will be allocated to CMO or LCM without consideration as to the randomisation of the family member already enrolled. The same will be true for the STI/continuous ART randomisation. This must be fully explained to family members where more than one is recruited into the trial.

No patient will be enrolled into NORA if there is already a household member in NORA.

7.4 Follow-up Evaluations

After randomisation to CMO/LCM (week 0) all patients will have follow-up visits to see both the study doctor and study nurse at weeks 2, 4, 8, 12 and then every 12 weeks from randomisation. The patient will collect a supply of drugs every 4 weeks from the nurse at the follow-up and intermediate visits (see Flow Sheets, section 1.3, page 16). For patients randomised to the STI arm, for the first 2 visits at start of STIs (weeks 52 and 76 or 76 and 100), patients will see both a doctor and a nurse at an extra visit, in order for decisions to be endorsed by the doctor about undertaking the next STI (see Flow Sheets, section 1.3, page 16).

At each full assessment, the following will be undertaken (see Flow Sheets, section 1.3, page 16):

- Administration of a symptom checklist by a nurse to detect intercurrent illness, HIV disease progression or adverse events to ART. The severity and likely relationship of events to ART, will be documented by a doctor.
- Medical history since last visit including signs and symptoms of HIV disease and WHO stage.
- Weight.
- Haematology and biochemistry (as at baseline). However, for patients in the CMO arm; for haematology grade 4 results (see 0, page 70), only the grade 4 result will be returned to the clinic; for biochemistry, if any of AST/ALT/bilirubin are grade 4, all 3 results will be returned to the clinic and, if either of urea/creatine are grade 4, both will be returned to the clinic. Clinicians may request biochemistry and/or haematology investigations for patients in the CMO arm who experience symptoms suggestive of severe toxicity to antiretroviral drugs they are receiving if the test result is essential in order to make decisions about clinical management (including switching drugs for

toxicity, e.g. suspected hepatitis (biochemistry), renal disease (biochemistry), pancreatitis (biochemistry)). Reasons for requesting laboratory tests must be clearly stated on the appropriate form.

- CD4 cell counts (in the CMO arm, results will not be returned to the clinician except at 48 weeks (72 weeks if the patient was not randomised at week 52) as <300 or ≥300).
- Plasma store and DNA pellets (see Appendix 7.0, page 88); stored plasma samples and DNA pellets may be used subsequently for HIV RNA viral load measurements, measurements of cell associated HIV DNA, HIV/HLA typing and analyses of drug levels.
- Assessment of adherence by pill counts and nurse administered questionnaire (see Appendix 8.0, page 89).
- Changes in ART, OI prophylaxis and other concomitant medication.
- For females of childbearing age allocated to the STI group, pregnancy tests will be performed one month before commencement of STI (see Flow Sheets, section 1.3, page 16).
- All women of childbearing age will be given continuing advice about avoiding pregnancy.
- If a woman becomes pregnant during the course of the trial, she will continue in the LCM or CMO arm like other patients, but if randomised in the STI arm, will ***NOT undergo*** a STI at any time during pregnancy, for 24 weeks after the end of pregnancy or while breast-feeding (STIs would be deferred until after the baby has been weaned). Advice on breast-feeding will be given according to national guidelines.
- Recording of compliance with the allocated management strategies on the follow-up form

At each 'drug collection' visit, the study nurse will:

- Administer the symptom check-list
- Assess adherence by questionnaire (see Appendix 8.0, page 89) and record numbers of returned pills
- Administer the next 4 weeks supply of drugs

If the study nurse has any clinical concerns at these visits, the patient should be referred to see a study doctor.

Patients switching to second-line (including those randomised in the second-line feasibility study) will have the visit schedule reset to week 0 at the time of switch. They will have the same visits as early after first randomisation primarily to provide additional checks for any adverse events associated with new drugs and to identify any problems with dosing etc.

7.5 Management of patients allocated to the STI arm

More details of management for the STI arm have been developed after the pilot phase of STI (see Appendix 1.0, page 49). Of note, STIs will take place during first-line ART only and patients in the STI arm will revert to continuous ART when they switch to second-line ART. For patients in the STI arm, there are two issues to consider: first, when to shorten an STI; second, when not to recommence an STI during follow-up.

7.5.1 Timing of STIs

Among patients randomised to STIs, follow-up visits will be due 4 weeks before commencing a STI. Thus CD4 count results at weeks 48 will be available at the week 52 visit and, after review by the study doctor, the STI will commence from week 52 (see Flow Sheets, section 1.3, page 16). For patients taking CBV+NVP, at each STI, CBV will be continued for 7 days after NVP is stopped.

7.5.2 Modifying the STI regimen

Criteria for early restart of ART include:

- Always restart for CD4 cell count <50 cells/mm^3, 8 weeks into STI (LCM only)
- Always restart for development of a WHO stage 3 or 4 diagnosis (Appendix 5.0, page 76).
- Consider restart for development of HIV related symptoms that do not meet the criteria for WHO stage 3 or 4 events, at the discretion of the treating clinician

If a patient develops clinical symptoms during a planned STI (detected at a nurse visit), then the nurse will refer the patient to be seen by the doctor. If the doctor assesses that the patient has a WHO stage 3 or 4 event, ART should be restarted. If the doctor assesses that the patient has clinical symptoms that do not meet the criteria for WHO stage 3 or 4 events, ART *may* be restarted at the discretion of the treating clinician but if the patient is in LCM, then the decision to restart therapy may also be deferred to take account of the CD4 cell count result (depending on the patient's health status).

For patients restarting ART early during a planned STI, consideration will be given to switching to second line ART if symptoms persist after ART is re-initiated, or CD4 count criteria (<50 cells/mm^3) continue to be met (LCM only), at the next scheduled CD4 count after recommencing ART (see section 7.6.2, page 37), because any symptoms or low CD4 count are likely to be a consequence of the interruption rather than failure of ART (which the patient was not receiving).

Patients who undergo a shortened STI for reasons other than WHO stage 3 or 4 or CD4<50 (LCM) may be reconsidered for further STIs at the next scheduled STI start: however, patients should only interrupt again if the clinician is confident that the patient has made a good recovery.

7.5.3 Cessation of STIs

Patients who experience a WHO stage 3 or 4 event or LCM patients who have a CD4 count <50 at any time after STI randomisation will not have any further STIs in the DART trial.

Patients who have switched to second line will not have any further STIs in the DART trial.

7.5.4 Deferring STI during follow-up

In the following situations an STI should always be deferred:

- CD4 count is <200 cells/mm^3 at 4 weeks before the scheduled STI start (for those who are randomised to LCM)
- Patient is pregnant, breastfeeding or within 24 weeks of the end of a pregnancy.
- The patient has not received 12 weeks back on continuous ART e.g. did not attend clinic for ART restart as scheduled.

In the event of any of the above, STI will be deferred until the next scheduled STI start (24 weeks later) before reassessment for consideration of another STI (or consideration of switching ART regimen – see section 7.6.2, page 37).

7.5.5 Termination of the STI study

On 16th March 2006, following a review of the STI data to 15 January 2006 by the DSMC and its subsequent report to the TSC, the STI randomisation was terminated and it was recommended that all clients randomised to STI be transferred to CT.

7.6 Changing ART

7.6.1 Substituting for Toxicity

Switching for severe clinical or laboratory toxicity will follow guidelines, based on clinical and laboratory grading of toxicities (0, page 70). A symptom checklist will include questions on nausea/vomiting, rash, headache, fever, jaundice, abdominal pain etc. In the CMO arm, laboratory tests will be done (at 4 and 12 weeks and then 12-weekly) but results will ***not be given back to clinicians*** unless a laboratory grade 4 adverse event occurs. A system will be set up at each site (with standard operating procedures (SOPs)) for feedback of grade 4 results only to clinicians for patients in the CMO group. Clinicians caring for patients allocated to CMO may request laboratory tests if indicated by clinical symptoms, as in routine practice. For guidelines on switching of individual drugs for toxicity, see section 8.1.4, page 40.

7.6.2 Switching for failure of first-line therapy

Physicians will be ***encouraged not to switch*** ART before 48 weeks on continuous ART, or within 12 weeks after recommencing ART after a planned STI. Every attempt should be made to ensure that patients are adherent to ART before a switch of ART is considered.

After 48 weeks, switching therapy can be considered at any time for those on continuous ART, and after at least 8 weeks back on ART for those on STI arm.

Clinical criteria for consideration of switching therapy include:

- The development of a new WHO stage 4 diagnosis (see Appendix 5.0, page 76)

CD4 cell count criteria (LCM arm only):

- CD4 cell count <50 cells/mm^3 on 2 occasions, while on ART (at least 8 weeks after restarting ART). I.e. if a CD4 cell count is <50 cells/mm^3 then it should be repeated as soon as practical and if still <50 cells/mm^3, the patient should switch ART.
- Consideration should be given to switching if the CD4 count on 2 consecutive occasions is below 100 cells/mm^3.

When a decision to change therapy has been taken, blood will be taken for haematology and biochemistry screens from all patients (CMO and LCM) to ensure there is no laboratory toxicity that would preclude the patient from taking the second-line therapy. A CD4 cell count will also be taken, but the result will not be returned to the clinician in the CMO arm, and a plasma specimen for future evaluation of HIV-RNA viral load and drug resistance will be collected.

When the clinical decision to switch has been made the patient (if they consent and are eligible) will be randomly assigned to a second line regimen/strategy according to section 7.3.4, page 33.

7.7 Assessment of adherence

A study nurse will assess adherence at each 4-weekly visit by pill counts and a nurse administered adherence questionnaire (see Appendix 8.0, page 89).

7.8 Withdrawal from allocated strategy

Patients may voluntarily withdraw from the allocated trial treatment for any reason. If this occurs, the trial researchers are not under obligation to provide ART. The patient's withdrawal from the trial will not affect their access to the best standard of care within the national health system. Follow-up for documentation of any clinical endpoints should continue, if possible.

Clinical data, including weight, presence of signs or symptoms, and other concomitant medications should be recorded at the time of withdrawal. In addition, a blood sample for plasma storage should be taken for storage (for subsequent viral load and possible resistance testing). Blood should also be taken for FBC, biochemistry and T-cell subsets (not returned to clinicians of patients in the CMO arm).

If a patient has an unscheduled period off treatment or not in follow-up, this should be fully recorded on follow-up forms.

7.9 Recording and reporting of Adverse Events, Death, HIV progression and protocol violations

If the patient has died, experienced any new or recurrent WHO stage 4 illness or experienced an adverse event (serious, Grade 3 or 4, or one leading to a modification of ART) since the last visit, the investigator/study co-ordinator will complete specific case report forms.

If there is any violation of the protocol for any reason, this should be fully recorded.

7.9.1 Serious Adverse Events (SAE):

According to the ICH Harmonised Tripartite Guidelines for Clinical Safety Data Management: Definitions and Standards for Expedited Reporting (1994), a Serious Adverse Event (SAE) is defined as 'any untoward medical occurrence' that:

1. **results in death**
2. **is life-threatening** (patient was actually at risk of death at the time of the event)
3. **requires unplanned inpatient hospitalisation** or **prolongation of existing hospitalisation**
4. **results in persistent or significant disability/incapacity** or is a congenital anomaly/ birth defect
5. any **other important medical condition, which, though not included in the above,** may jeopardise the subject and may require medical or surgical intervention to prevent one of the outcomes listed (e.g. intensive treatment in hospital or at home for allergic bronchospasm; blood dyscrasia or convulsions that do not result in hospitalisation, or the development of drug dependency)

In the context of the DART trial SAEs need only be reported if considered **not** HIV-related **only.** All such SAEs must be recorded on the Serious Adverse Event form and reported to the MRC CTU as soon as possible after the event.

The MRC CTU will report SAEs that are definitely or probably related to antiretrovirals to the relevant pharmaceutical companies and/or to the regulatory authorities in timely fashion, as appropriate.

National requirements for reporting of SAEs will be followed.

Data on SAEs will be tabulated and presented to the Data and Safety Monitoring Committee (DSMC; see section 9.4, page 43).

Other grade 3 and 4 adverse events (see 0, page 70) should be reported on the next scheduled follow-up.

8.0 Drug Regimens

8.1 Antiretroviral Therapy

Table 5 First and second-line drug regimens for DART (For details see Appendix 3.0, page 66)

Patient numbers	First-line treatment	Second-line treatment
2700	CBV plus TDF or ABC (and no evidence of prior failure on an NNRTI)	boosted PI* plus NNRTI** ±ddI/3TC
600	CBV plus NVP	boosted PI* plus ABC+ddI or TDF+CBV

* boosted PI will normally be Lopinavir/r (Aluvia or Kaletra)

** NNRTI will preferably be nevirapine for women of childbearing age.

8.1.1 First-line antiretroviral drugs

All patients will receive a triple combination of ART throughout the trial. All patients will receive combivir as part of their first-line regimen. The third drug for 600 patients enrolled in the NORA substudy will be blinded ABC or NVP plus placebo (see Appendix 2.0, page 52). For all patients not enrolled in the substudy TDF and NVP will be available as the third first-line drug for patients. Patients with any absolute (e.g. on concomitant anti-tuberculosis therapy) or relative (e.g. receipt of previous NVP in pregnancy) contra-indications to receipt of NVP, should receive TDF as the third drug.

TDF and NVP will be allocated equally between Zimbabwe and Uganda. It is expected that patients will stay for about 2.5 years on each regimen. Upon failure of a second-line regimen, the best possible combination will be selected from first and second-line drugs for the remaining duration of the trial. After trial completion follow-up healthcare will be provided by the national health systems (see Appendix 9.0, page 90, and letters from the Ministries of Health).

8.1.2 Second-line antiretroviral drugs

Second-line ART will be provided according to the feasibility randomisation strategy assigned (section 7.3.4, page 33). For those patients who have already switched to second-line ART prior to the introduction of the second line randomisation or are not eligible or choose not to consent for randomisation, second line ART will be provided according to the decision of the treating clinician from the regimens included in the feasibility study

8.1.3 Provision of trial drugs

Patients will be provided with a 4-weekly supply of drugs throughout the trial. Patients will be requested to return all empty bottles and to bring any bottles in use to the follow-up clinic.

On no account should any drug assigned to one patient be used by another patient. Unused drug must be returned to the site if a patient withdraws from treatment.

All drug dispensed and returned to the site should be documented on a treatment log for each patient. At each site, a named person (trial pharmacist or research nurse) will be required to maintain complete records of all study medication dispensed. The procedures to be followed will adhere to the Good Clinical Practices (GCP) guidelines on drug accountability. MRC CTU will monitor drug accountability at site visits.

8.1.4 Modification of therapy for toxicity

For reactions where the cause may be attributed to one or more trial drugs, all drugs should be stopped temporarily and may be restarted if the symptoms resolve and this is appropriate. If the symptoms are intolerable or do not resolve, an alternative drug **of the same class** may be substituted if this is considered appropriate by the investigator (see Table 6 below) and other drugs restarted. ZDV and 3TC will be available as separate drugs for patients who need to stop one drug for toxicity. See also 0, page 70.

Table 6 Guidelines for Substituting for Toxicity in First-Line

Event	Switch
ZDV toxicity	Substitute ZDV with d4T (replace combivir with d4T + 3TC)
3TC toxicity	Substitute 3TC with ddI (replace combivir with ZDV + ddI)
NVP toxicity	Substitute NVP with TDF
ABC toxicity	Substitute ABC with NVP or TDF
TDF toxicity	Substitute TDF with NVP
ddI toxicity	likely to be 3TC (individual judgement)
d4T toxicity	likely to be ZDV (individual judgement)

Note: due to potential for lymphopenia ddI and TDF should not be used together. If absolutely necessary to use them together the dose of ddI should be reduced. Substitutions in second-line will follow this where possible, but will depend on individualised drug history.

8.2 Prophylaxis against Opportunistic Infections

Decisions on prophylaxis strategies will be made taking into account National guidelines. All prophylaxis therapy should be recorded on forms.

8.3 Medications not permitted/ Precautions

Patients should not be co-enrolled in other ART trials or receive ART outside the trial. Patients will be encouraged to seek advice prior to taking any other medication. See Appendix 3.0, page 66 for details of drug interactions.

8.4 Data on concomitant medications

At each visit, information on other medications, including start dates, and reason(s) for taking should be documented on follow-up forms.

9.0 Management of the Trial

9.1 Trial Steering Committee

The trial will be managed by a Trial Steering Committee (TSC) with an independent chairperson (Professor PPD independent members and one Principal Investigator or key investigator from each site and from the MRC CTU and Imperial College.

A subgroup of the TSC will undertake monitoring of the STI pilot data, see Appendix 1.0, page 49.

In addition to the above, a subcommittee of the TSC will be formed to undertake close monitoring of CD4 cell counts on a monthly basis among patients in the CMO and STI arms of the study (after completion of the STI pilot). The reason for this is to ensure that asymptomatic patients with very low CD4 cell counts are not continuing on STIs and therefore being at high risk of developing clinical progression. Should this occur, the subcommittee could convene a meeting of the DSMC to consider its findings and would advise the Trial Steering Committee about appropriate action, e.g. change of trial design to shorten the period of STIs.

9.2 Trial Management Groups

An International Co-ordinating Group (ICG) of principal and key investigators from each site, as well as members from MRC CTU will communicate regularly to ensure that the trial is proceeding well across all 3 sites. Local Trial Management Committees (LTMCs) composed of investigators at each site will meet regularly. There will be regular teleconferences to ensure good communication across sites and with MRC CTU and Imperial College.

9.3 Data Management and Monitoring

An Analysis and Data Management Committee will be set up with data management, computing and statistician members from each site and the MRC-CTU, chaired by the Trial Statistician. This Committee will be responsible for setting up the databases at each site and for co-ordination of timely merging of data from each site at MRC CTU, where the central database will be held. The committee will be responsible for ensuring that the system for data collection is working consistently across the sites, for developing the trial analysis plan and ‘shell’ tables to be provided to the DSMC, and for making decisions about analyses.

Each site will be responsible for maintaining its own database and for timely transfer of checked data to the MRC CTU for merging of data with those from the other sites. Staff from MRC CTU will visit clinical sites to validate and monitor data and this may also be done across sites (e.g. a data manager from Zimbabwe may visit Uganda), under the oversight of the Analysis and Data Management Committee. The clinical investigators and participants, by giving consent, agree that within the host country's Data Protection Law, the MRC CTU may consult and /or copy source records (clinical notes, laboratory values) in order to do this. Such information will be treated as strictly confidential and will in no circumstances be made publicly available. The monitoring will adhere to MRC Good Clinical Practice guidelines (based on ICH guidelines). The following data should be verifiable from source documents: signed consent forms; dates of visits including laboratory results; eligibility and baseline values for all patients; all clinical endpoints; all serious/severe adverse events; an ongoing random 10% sample of routine patient clinical and laboratory data; drug compliance; dates drug dispensed and (if necessary) drugs returned; pharmacy/clinic drug logs; concomitant medication.

9.4 Data and Safety Monitoring Committee (DSMC)

An independent Data and Safety Monitoring Committee (DSMC) will be established and will monitor all aspects of the trial including the NORA substudy (see Appendix 2.0, page 52). The DSMC will consider findings from any other relevant studies and review trial data on recruitment, safety, adherence to randomised strategies and efficacy, in strict confidence approximately every 6 months. The DSMC will report to the DART Trial Steering Committee and to the Ethics Committee in each country, if in their view the data provide proof beyond reasonable doubt that one of the allocated strategies is better than its comparator in terms of a difference of clinically significant magnitude in a primary outcome. The guiding statistical criteria for "proof beyond reasonable doubt" is a Haybittle-Peto type rule based on the 99.9% confidence interval of the relative hazard of disease progression in each interim analysis. The DART Trial Steering Committee will then decide whether to amend or stop the trial before the end of the planned follow-up. In addition, the DSMC will meet with the DART Trial Steering Committee to consider results from the STI pilot study. The decision whether to go ahead with the evaluation of the planned STI strategy or an amended strategy will be taken jointly by the two committees in that meeting. The MRC CTU will undertake the organisation of the DSMC meetings.

9.5 Endpoint Review Committee

An Endpoint Review Committee will be appointed whose remit will be to determine the validity of potential endpoints that do not clearly satisfy the standard criteria, as defined by the protocol. It will have an independent Chair and will include Project Leaders from each site as well as other clinicians. No member will review endpoints from their own site. Terms of reference for the Endpoint Review Committee will be drawn up.

10.0 Statistics

10.1 Sample Size

The aim is to recruit 3000 patients (1000 from each of the 3 sites – 2 in Uganda, 1 in Zimbabwe) during the first year. Patients will be allocated in a 1:1 ratio to LCM or CMO. Data from several studies co-ordinated by the MRC Clinical Trials Unit (CTU) in the UK in adults show that among patients starting triple ART with CD4 counts <50, 50-99, 100-199, the proportions with CD4 >200 cells/mm^3 are 20%, 43%, 73% respectively at 6 months after starting ART and 33%; 68%, 77% respectively at 12 months. Thus if the proportions of patients with CD4 counts in these three ranges are equal, we would expect 60% of patients to have CD4≥200 cells/mm^3 and to qualify for the second randomisation to STI or no STI, based on a threshold of 200 cells/mm^3 (n=1800).

The estimation of the sample size is based on the following assumptions:

1. Progression rate to a new stage 4 disease or death is 15% per year in the LCM arm. This is based on data from the PPD cohort which suggest that the cumulative proportion of untreated patients with CD4 count less than 200 cells/mm^3 progressing to WHO stage 4 or death is 0.55, equivalent to a progression rate of 80% per year. A progression rate of 15% per year therefore represents a reduction of more than 80% under ART.

2. Progression rate to a new stage 4 disease or death in patients eligible for the second randomisation on continuous ART is 10% per year (this is lower than the overall progression rate as these patients have achieved CD4 cell counts >200 cells/mm^3).
3. Recruitment is over 1 year and follow-up for a further 4 years. For the second randomisation recruitment is over 2 years and follow-up for at least 3 years.
4. Loss to follow-up rate 3.3% per year, equivalent to approximately 15% cumulative proportion of patients lost to follow-up by 5 years.
5. Type I error probability (alpha) 0.05 (two sided)
6. A progression rate of up to 17.5% per year in the CMO arm would be considered 'equivalent' to that in LCM (15%).
7. A progression rate of up to 13% per year in the STI arm would be considered 'equivalent' to that in the no STI arm (10%).
8. Power 80% to detect equivalence of LCM and CMO, i.e. the upper limit of the 95% confidence interval of the hazard ratio (CMO relative to LCM) will be no greater than 1.17 (a relative increase of 17%, equivalent to an annual rate of progression of 17.5% in the CMO arm compared to 15% in the LCM arm) with probability 0.80 if CMO and LCM were truly equivalent.
9. Power 80% to detect equivalence of STI and no STI, i.e. the upper limit of the 95% confidence interval of the hazard ratio (STI relative to no STI) will be no greater than 1.3 (a relative increase of 30%, equivalent to an annual rate of progression of 13% in the STI arm compared to 10% in the no STI arm) with probability 0.80 if STI and no STI were truly equivalent.

Under these assumptions, a total of 2862 would be required for the LCM versus CMO comparison and 1520 for the STI versus no STI comparison. The target number of events (progression to a new stage 4 event or death) is 1392 for the LCM versus CMO comparison and 501 for the STI versus no STI comparison. An additional 100 patients would be required for the STI pilot study.

Assuming that recruitment into the LCM versus CMO comparison takes place through the first two years rather than one year, a total of 3101 patients (rather than 2862) would be required for the LCM versus CMO comparison.

Assuming the STI versus CT randomisation is based on a threshold of 300 CD4 cells/mm^3 rather than 200 cells/mm3, thus in DART so far we would expect around 20% of patients to be eligible for the second randomisation at 48 weeks. Excluding those already included in the STI pilot this would leave at least 600 patients eligible for the second randomisation.

Assuming:

10. A progression rate of up to 16% per year in the STI arm would be considered 'equivalent' to that in the no STI arm (10%).
11. Power 80% to detect equivalence of STI and no STI, i.e. the upper limit of the 95% confidence interval of the hazard ratio (STI relative to no STI) will be no greater than 1.6 (a relative increase of 60%, equivalent to an annual rate of progression of 16% in the STI arm compared to 10% in the no STI arm) with probability 0.80 if STI and no STI were truly equivalent.

Then 556 patients would be required for the STI versus no STI comparison (target number of events 159).

Sample size for trial extension to end 2008

At the time the trial was designed (2001) there were no data on likely rates of disease progression/death in patients in Africa receiving effective ART: the 15% progression rate per year described above represented a 80% reduction from the rates observed in patients with CD4 <200 cells/mm^3 in the (natural history) PPD cohort, similar reductions to those observed on a population level in cohorts in industrialised countries (e.g. CASCADE, EuroSIDA).

However, it has become apparent that the overall rate for the primary endpoint - progression to new WHO stage 4 or death - is considerably lower than predicted (approx 10% rather than 15% per year for the randomised groups combined), reducing the power from 80% to only 65% with the existing sample size and a 5 year trial. The one year extension of total trial duration (from 5 to 6 calendar years, to end 2008), maintains 80% power to demonstrate equivalence with a definition of 10% per year ±1.8% (hazard ratio 1.18) (or to demonstrate equivalence according to the original design (10% per year ±1.7%, hazard ratio 1.17) with power of 72%).

Trial closeout: the power calculations above are based on an additional year follow-up for each participant (i.e. until end 2008). However, given the size of DART, it will not be possible for the trial to close quickly in December 2008 as participants will need to be transferred into national ART programmes. Therefore, participants who have been in the trial longest will start to move onto national ART programmes through the last quarter of 2008, with final transfer of all participants by the end of the first quarter of 2009 (which would retain the total number of additional person years of follow-up required).

Second-line randomisation feasibility study:

The sample size available for this feasibility study is constrained by the fact that only patients failing first-line therapy in DART and switching to second-line therapy will be eligible. Thus the sample size calculation below shows the differences that could be detected given the expected number of patients failing first-line during mid-2007 to last quarter 2008 and thus having at least 24 weeks follow-up. **The intention of the feasibility study is to identify the most promising regimens, based on CD4 response, to potentially take forward into a larger, subsequent clinical endpoint trial.** Currently the failure rate is approximately 5% per annum after the first year on first-line therapy. Assuming this rate increases at 2% per annum until the end of the trial, we estimate that at least 240 patients will switch to second-line therapy during mid-2007 to 2008 (and will have 24 weeks follow-up by the end of 2008), although the exact numbers and split between NNRTI and NRTI failures are unknown (estimated 15-20% versus 85-80%) – and could increase dramatically if the failure rate increased.

Optimal HAART regimens

If we assume (as observed from 0 to 24 weeks in DART) that the standard deviation for the change in CD4 from switch to second-line to 24 weeks is 100, a total of 190 participants that had not failed on an NNRTI, randomised between 3 arms (C,D,E), would provide at least

80% power to detect a difference in change of CD4 from baseline of more than 55 cells/mm^3 between any 2 of the 3 groups (based on 2-sided alpha=0.025 to allow for multiple testing of 3 pairwise comparisons) assuming 5% missing data (loss to follow-up/death during the first year after randomisation to second-line therapy plus failure to attend the week 48 visit/missing sample). A total of 50 patients that had failed on an NNRTI, randomised between 2 arms (A&B) would provide 80% power to detect a difference of 80 cells/mm^3 between these 2 arms as significant at the 2-sided 5% level, again assuming 5% missing data. The choice of 24 weeks as endpoint is based on large increases in CD4 observed at initiation of 2nd line in DART, therefore any ineffective regimens are likely to be identified quickly.

10.2 Analysis Plan

The primary analysis will compare LCM with CMO and STI with no STI as allocated (intention to treat (ITT)) in terms of:

a) Progression to a new HIV stage 4 event or death and

b) Drug-related grade 4 serious adverse events

Time-to-event methods (Kaplan-Meier plots, stratified log rank test and Cox proportional hazard regression) will be used for these comparisons. The frequency of grade 4 adverse events will be tabulated by body systems and randomised group and the groups will be compared using the X^2 test. Wilcoxon's rank-sum test will be used to compare median CD4 cell counts at 3 years in the different allocated strategies.

For the second-line feasibility study, the primary analysis will compare

(1) Arm A versus Arm B (optimised HAART: NNRTI failures)

(2) Arm C versus Arm D versus Arm E (optimised HAART: NRTI failures)

(All analyses will be conducted based on all randomised patients (ITT).

11.0 Regulatory/ Ethics Approval

This document, along with any subsequent modifications and with the sample informed consent documents will be reviewed by the Ethics Committee from each participating site as well as by Ethics Committees in the UK (Liverpool School of Tropical Medicine and Imperial College).

Regulatory approval for conduct of the trial and use of antiretrovirals in the trial will be obtained.

12.0 Confidentiality

A unique trial number will identify all laboratory specimens, case record forms, and other records and no names will be used, in order to maintain confidentiality. All records will be kept in locked locations. Clinical information will not be released without written permission, except as necessary for monitoring by the trial monitors.

13.0 Publication

The ICG will develop guidelines for the preparation of papers (including abstracts) for presentation at national and international meetings, as well as the preparation of manuscripts for peer-reviewed publication. Any publication or presentation during the active phase of the study must have prior approval of the TSC. The TSC will define the strategy for, and resolve any problems of authorship and maintain the quality of publications. All publications will acknowledge appropriate funding sources. The DART TSC is the custodian of the data and specimens generated from the DART trial; DART trial data are not the property of individual participating investigators or health care facilities where the data were generated.

14.0 References

1. Altfeld M, Rosenberg ES, Walker BD. Cellular Immune Responses and Viral Diversity in Individuals Treated during Acute and Early HIV-1 Infection**.** *J. Exp. Med.* 2001 193: 169-180.
2. Lori I, Maserati R, Foli A et al. Structured treatment interruptions to control HIV-1 infection. *Lancet* 2000, 354, 287.
3. Lisziewicz J, Rosenburg E, Lieberman J et al. Control of HIV despite the discontinuation of antiretroviral therapy. *N Engl J Med*, 1999, 340, 1683.
4. Rosenburg ES, Altfeld M, Poon SH, et al. Immune control of HIV-1 after early treatment of acute infection. *Nature* 2000, 407: 523-526.
5. Amie C et al. Treatment interruptions in heavily antiretroviral pre-treated patients: clinical and genotypic evolution. *First International AIDS Society Conference on HIV Pathogenesis and Treatment*, Buenos Aires, abstract 445, 2001.
6. Deeks SG, Wrin T, Liegler T, Hoh R, Hayden M, Barbour JD, Hellmann N, Petropoulos CJ, McCune JM, Hellerstein MK, Grant RM. Virologic and Immunologic Consequences of Discontinuing Combination Antireroviral-Drug Therapy in HIV-Infected Patients with Detectable Viremia. *N Eng J Med*, Feb 2001; 344:472-480.
7. Ruiz L, Martinez-Picardo J, Romeu J, Paredes R, Khalil Zayat M, Marfil S, Negredo E, Sirera G, Tural C and Clotet B. Structured treatment interruption in chronically HIV-1 infected patients after long-term viral suppression. *AIDS* 2000, 14: 397-403
8. Wallace M et al. Structured treatment interruption: effect on triglyceride levels. *First International AIDS Society Conference on HIV Pathogenesis and Treatment*, Buenos Aires, abstract 448, 2001.
9. Youle M, Janossy G, Turnbull W, Tilling R, Loveday C, Mocroft A, Tyrer M, Madge S, Wilson D, Dykhoff A, Johnson M, Phillips A, for the Royal Free Centre for HIV Medicine. Changes in CD4 lymphocyte counts after interruption of therapy in patients with viral failure on protease inhibitor-containing regimens *AIDS* 2000, 14(12): 1717-20
10. Scaling up antiretroviral therapy in resource limited settings: guidelines for a public health approach. 2002. http://www.who.int/hiv/pub/prev_care/en/ScalingUp_E.pdf ISBN 92 4 154570 4

11. Schooley RT, Ruane P, Myers RA et al for the Study 902 Team. TDF DF in antiretroviral-experience patients: results from a 48-week, randomised, double-blind study. *AIDS* 2002, 16: 1257-1263.

12. C Allavena et al. 2-Year Follow-Up Lopinavir/ritonavir (LPV/r-Efavirenz (EFV) Combination. Abstract WePe6.3C06 (poster). Program and Abstracts of the 3rd IAS Conference on HIV Pathogenesis and Treatment. July 24-27, 2005. Rio de Janeiro, Brazil

Appendix 1.0 Structured Treatment Interruptions (STI) Pilot Study

A pilot study will be undertaken to provide preliminary data on the safety of STI in this patient population in whom antiretroviral therapy is started at a relatively late stage of HIV disease. Therefore, ***before commencing the second randomisation to either structured treatment interruption (STI) or to continuous ART (no STI),*** the first 100 patients (from either the CMO arm or the LCM arm, at any of the three study sites) with CD4 cell counts >=200 cells/mm^3 at week 24 will be enrolled into a non-randomised pilot study and undergo a treatment interruption of 12 weeks, followed by restarting therapy for 12 weeks.

1.1 Monitoring of individual patients

During this 24-week period the patients will be seen by the doctor every 4 weeks and 4-weekly CD4 cell counts will be performed on all 100 patients and reported to the clinicians in the LCM arm.For CMO patients in the STI pilot the Trial Manager at each site will check all CD4 counts while on STI and if any are <50 cells/mm^3 they will report the result to the clinic as <50 cells/mm^3). These 100 patients will subsequently be excluded from the analysis of the STI versus continuous ART randomisation, **but not from the CMO versus LCM randomisation.** At the end of the 24 weeks, patients will remain in their original LCM/CMO arm and will continue with STIs. Patients will continue to receive drugs and follow-up as in the main DART study.

1.2 Monitoring of the group data

Two members of the TSC who are not involved with clinical management of DART patients will look at individual CD4 counts for all patients enrolled in the STI pilot after each 2-weekly data merge, **without** knowledge of CMO/LCM allocation.

The data will be summarised by CD4 at baseline (before ART) and before the STI, to describe the following:

- the rate of decline of CD4
- the proportion with new stage 3 or 4 WHO events,
- the proportion of patients with CD4 <50 cells/mm^3
- the proportion with a reduction in CD4 of 150 cells/mm^3 or greater from the start of the STI.

If the rate of decline were considered too fast (given baseline and pre-STI values) by the 2 TSC members, or if, for example, >10% patients in the STI experienced any of the above outcomes, this would be further discussed with the chair of the TSC. They could decide whether they need to inform the DSMC and TSC immediately, prompting a full analysis of all data accrued in the STI pilot to this point in time in the STI pilot for urgent consideration by the DSMC.

After the 100th patient has been enrolled and completed 12 weeks STI, the data will immediately be analysed and considered jointly by the Trial Steering Committee and the DSMC, to determine whether it is safe to start the second randomisation (STI or continuous ART). The Committees may be satisfied about the safety of STI either after reviewing data

on 12 weeks of STI on all 100 patients, or they may recommend waiting until the results of the effect on CD4 count of restarting ART for a further 12 weeks after the STI are available on all 100 patients before making a decision about commencing the second randomisation.

1.3 Guidelines for management during STI pilot.

1. If CD4 count falls to <50 cells/mm^3, repeat within 2 weeks, and if still <50 cells/mm^3, then the patient should be restarted on ART for LCM arm
2. Clinical criteria (see Appendix 5.0, page 76) for restarting ART during a STI would include for LCM and CMO arms:
    - ♦ the development of a new WHO stage 3 or 4 diagnosis

(NB. if CD4 cell count is not low (e.g. >150 cells/mm^3), despite the presence of symptoms consistent with stage 3 or 4 disease, the clinician may decide to continue STI, and repeat CD4 cell count in 4 weeks. If by then the CD4 count has declined or if symptoms do not resolve, ART should be restarted.

1.4 Guidelines for the DSMC and Steering Committees.

If any of the following occurred during the pilot STI study, this would be of concern to the TSC and DSMC and would suggest that a revision of the STI trial design would be necessary:

1. If a significant number of patients (e.g. >10%) develop the following during an STI:
    a. Progression to new stage 3 or stage 4 event
    b. CD4 count <50 cell/mm^3
    c. Reduction in CD4 cell count of 150 cells or more from the value at the start of the STI

2. Analyses would be undertaken to explore the relationship between development of the above and baseline characteristics, including baseline CD4 cell count. The committees may recommend a change to the trial design, depending on the group of patients in whom this occurs. For example, they could recommend:
    a. Only enrol those with CD4 cell counts above 100 cells/mm^3 at baseline into the trial
    b. Shorten the period of STI e.g. 12 weeks on and 8 weeks off ART
    c. In any event, they may recommend continuation of the pilot study with the new design.

1.5 Flow Sheet for STI pilot

Table 7 STI Pilot for 100 patients (completed 24 weeks in CMO or LCM and with CD4 cell counts at week 24 of 200 cells/mm^3 or higher.

EVENTS	WEEK of STI pilot study						
	Start of STI<br>0<br>(Week 28 in trial)	4<br>(32)	8<br>(36)	Restart ART<br>12<br>(40)	16<br>(44)	20<br>(48)	Restart STI<br>24<br>(52)
History & Physical[1]	**X**	**X**	**X**	**X**	**X**	**X**	**X**
Symptom check list	**X**	**X**	**X**	**X**	**X**	**X**	**X**
Pregnancy Test[2]	**X**					**X**	
Haematology[3]	**X**	**X**	**X**	**X**	**X**	**X**	**X**
Biochemistry[4]	**X**		**X**	**X**		**X**	**X**
Lymphocyte Subsets [5]	**X**	**X**	**X**	**X**	**X**	**X**	**X**
Plasma storage[6] + DNA pellet	**X**	**X**	**X**	**X**	**X**	**X**	**X**
Adherence assessment[7]	**X**				**X**	**X**	**X**

1 Clinical: including weight, WHO staging for HIV. Doctor sees patient at every visit.
2 If positive, exclude from STI pilot study.
3 Haematology: Hb, MCV, WBC, Lymphocytes, Neutrophils, and Platelets.
4 Biochemistry: Blood Urea Nitrogen or Creatinine, AST or ALT, Bilirubin,– results to be given back for all patients in the pilot.
5 CD4, CD8, CD3, percentage and total lymphocyte count.
6 Take 6 ml blood into EDTA. Store DNA pellet (if feasible) See Appendix 7.0, page 88 for instructions about storage.
Take plasma at the time of change to second-line regimen (and DNA pellet if feasible).
7 See Appendix 8.0, page 89.

Appendix 2.0 Nevirapine OR Abacavir (NORA) Substudy

A randomised, double-blind, phase II (substudy) trial to evaluate the toxicity of Abacavir compared with Nevirapine, both in combination with ZDV+3TC (combivir), as first-line antiretroviral therapy in patients participating in the DART trial

2.1 Objective

To assess the safety of abacavir (ABC) compared with nevirapine (NVP), (both in combination with ZDV+3TC (combivir)) in African patients.

2.2 Background and Rationale

2.2.1 Hypersensitivity reaction to Abacavir

In clinical studies, approximately 3-5% of patients receiving ABC develop a hypersensitivity reaction. This is characterised by symptoms which usually appear within the first six weeks of initiation of treatment with ABC (median time to onset is 11 days) and most often include fever, rash, gastrointestinal symptoms (nausea, vomiting, diarrhoea, or abdominal pain), and lethargy or malaise. Other signs and symptoms may include respiratory symptoms (dyspnoea, sore throat, cough), musculoskeletal symptoms (myalgia, rarely myolysis, arthralgia), headache, paraesthesiae and oedema. Some patients with hypersensitivity reactions were initially thought to have respiratory disease (pneumonia, bronchitis, pharyngitis) or a flu-like illness.

The rash is variable and may be absent, but often appears maculopapular or urticarial. Laboratory abnormalities that may accompany ABC hypersensitivity include abnormal liver function tests or elevated creatinine phosphokinase or creatinine or lymphopenia.

Symptoms related to the hypersensitivity reaction worsen with continued therapy but usually resolve upon discontinuation of ABC. Restarting ABC following a hypersensitivity reaction results in a prompt return of symptoms within hours. This recurrence of the hypersensitivity reaction may be more severe than on initial presentation and may include life-threatening hypotension and death. Subjects who develop a hypersensitivity reaction must not be re-challenged with ABC.

As ABC hypersensitivity is heralded by the onset of relatively vague symptoms and signs, it could be mistaken for an acute intercurrent infection (e.g. malaria or flu), or be confused with immune reconstitution disease. Standard practice in the West (where rates of intercurrent infection are much lower, and there is likely to be less endemic sub-clinical infection to precipitate immune reconstitution disease), is to stop ABC for symptoms consistent with hypersensitivity and not to restart the drug. High rates of possible reactions that cannot be delineated further clinically could render ABC difficult to use in Africa, or substantially reduce its role. However, it is possible that even if ABC is difficult to use as first-line therapy because of difficulties distinguishing hypersensitivity from immune reconstitution disease, it might be possible to use it sometime after 3 or more months into ARV treatment when the risk of immune reconstitution (IR) disease is lower. Reasons for doing this might include the desire to switch to a simpler regimen (ABC is available in combination with ZDV and 3TC as a single formulation, taken as 1 tablet twice daily) or the need to start antituberculosis therapy. It could also be useful in a second-line regimen. No study has been carried out to address these questions and no data are available.

2.2.2 Adverse reactions to NVP

Signs and symptoms of NVP reaction include rash with or without fever, general malaise, fatigue, muscle or joint aches, blisters, oral lesions, conjunctivitis, facial oedema, and/or hepatitis, eosinophilia, granulocytopenia, and renal dysfunction.

The most common clinical toxicity of NVP is rash, which occurred in 15-20% of subjects in phase I/II trials. Severe and life-threatening skin reactions, including fatal cases, have occurred in 2-5% subjects treated with NVP. These have included cases of Stevens-Johnson syndrome, toxic epidermal necrolysis, and hypersensitivity reactions characterised by rash, constitutional findings and organ dysfunction.

Rashes are usually mild to moderate, maculopapular erythematous cutaneous eruptions, with or without pruritus, located on the trunk, face and extremities. The majority of rashes occur within the first 6 weeks of therapy, and severe rashes occur earlier, usually within the first 28 days of treatment; 25% of the patients with severe rashes required hospitalisation. Overall, 7% of patients in clinical trials discontinued NVP due to rash.

In one clinical trial, concomitant use of prednisone to prevent NVP-associated rash increased the incidence and severity of rash during the first 6 weeks of NVP therapy. The use of prednisone to prevent NVP-associated rash is not recommended.

Elevation of liver enzyme levels frequently occurs during drug therapy with NVP. Hepatitis and cases of fatal hepatic toxicity have been reported but are rare.

2.3 Trial Design

It is proposed to undertake this substudy in 600 patients enrolled in the DART trial. Separate consent will be sought for the substudy. Patients will be randomised to ABC (plus NVP placebo) or NVP plus ABC placebo in a 1:1 ratio, stratified by allocation to CMO/LCM arms. The blinded part of the trial will take place over 6 months, during which no patients will undergo STIs.

The trial sites will be in PPD Uganda and PPD Uganda. These are 2 of the 3 sites for the DART trial.

2.4 NORA Trial Schema

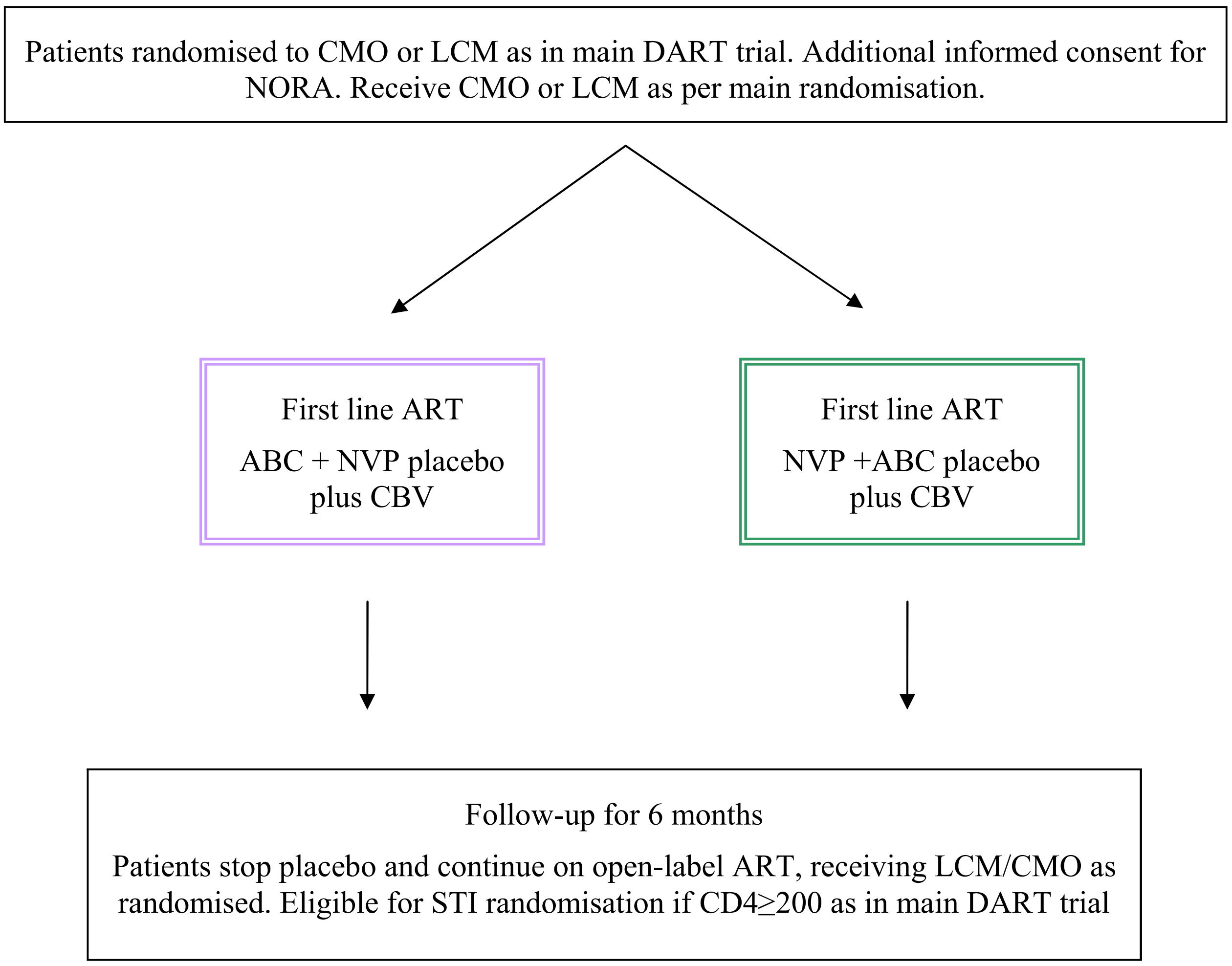

2.5 Endpoints

2.5.1 Primary endpoint:

- Any serious adverse event that is definitely/probably or uncertainly related to blinded trial drugs (blinded ABC or blinded NVP) where the adverse event occurs while on ART or within 30 days of receiving ART. A SAE in the context of the DART trial is defined as any untoward medical occurrence that is not definitely HIV related only and either:
    - results in death
    - is life-threatening (patient was actually at risk of death at the time of the event)
    - requires unplanned inpatient hospitalisation or prolongation of existing hospitalisation
    - results in persistent or significant disability/incapacity
    - is any other important medical events considered serious by the investigator (e.g. intensive treatment in an emergency room or at home

for allergic bronchospasm; blood dyscrasia or convulsions that do not result in hospitalisation)

All suspected hypersensitivity reactions must be reported as a SAE, these should be categorized as “other important medical conditions” unless they fall into one of the more specific categories.

2.5.2 Secondary Endpoints

- Adverse events of any grade leading to permanent stopping of trial drug:
- Fever, rash or raised liver enzymes (blinded in CMO except grade 4) leading to discontinuation of trial drug.
- Grade 4 events irrespective of whether they result in stopping trial drug

Adverse events will be classified by whether they are definitely/probably related, uncertainly related or unrelated/unlikely to be related to trial drug.

2.6 NORA Criteria for Inclusion/Exclusion

2.6.1 Inclusion Criteria

1. Documentation of HIV-1 infection: antibody positive serology by ELISA test (confirmed by licensed second ELISA or Western Blot)
2. Age > 18 years
3. Symptomatic WHO stage 2, 3 or 4 HIV disease and CD4 < 200 cells/mm3
4. ART naïve (except for ART use during pregnancy for the prevention of mother-to-child HIV transmission)
5. Agreement and documented informed consent to participate in DART main study and willingness to be randomised to ABC or NVP, if eligible
6. Life expectancy of at least 3 months

2.6.2 Exclusion criteria

1. Cannot or unlikely to attend regularly (e.g. usual residence too far from Study Centre)
2. Likelihood of poor compliance
3. Presence of acute infection (e.g. malaria, acute hepatitis, pneumococcal pneumonia, non-typhoid salmonella septicaemia, cryptococcal meningitis, acute hepatitis). Patients may be admitted after recovery of an acute infection. Patients with tuberculosis (TB) will not be enrolled while on the intensive phase of anti-tuberculosis therapy, but should be re-evaluated after the intensive phase and a decision made then about starting ART. Patients starting ART whilst on anti-tuberculosis therapy after the intensive phase will not receive NVP, nor will they be randomised into the NORA substudy.
4. On chemotherapy for malignancy
5. Laboratory abnormalities which are a contra-indication for the patient to start ART (e.g. Haemoglobin <8g/dl, neutrophils $<0.50 \times 10^9/l$, AST or ALT >5 x the upper limit of normal (ULN), grade 3 renal dysfunction - creatinine >360 μmol/l and /or urea >5 x ULN).
6. Pregnancy or breast-feeding

7. Family/household members already enrolled in NORA

Patients included in this substudy should not be included in the STI pilot substudy

2.7 Sample size

Data from a cohort of 70 patients on ddI/d4T/NVP, over an average follow-up of 6 months; suggest a cumulative proportion of 15% of patients experiencing an SAE during the first 24 weeks (unpublished data from Cascade). 300 patients per arm will be sufficient to detect a difference between 15% in one arm and 8% in the other arm with power 80% using a two-sided chi-square test of difference in proportions with alpha = 0.05.

The substudy will last for 6 months. After that patients will switch to unblinded drug and will remain on the monitoring arm they were randomised to before the substudy and will become eligible for the STI randomisation, providing their CD4 count is 200cells/mm^3 or higher. Drug supply, patient management and follow-up will continue as in the main DART study.

2.8 Analysis Plan

The frequency of SAEs, all grade 4 adverse events and all adverse events leading to discontinuation of trial drug will be tabulated by body systems and the two randomised groups will be compared in terms of the proportion of patients experiencing any of the primary and secondary outcomes of the substudy by week 24, using the X^2 test, stratified by allocation to CLM or CMO. Kaplan-Meier plots and log-rank test will be used to compare the two groups in terms of time to first SAE.

2.9 Management of Adverse reactions

All patients will be followed up as in the main DART trial and given advice about reactions to ZDV, 3TC and NVP. They will also be given standard advice and warnings about possible reactions to ABC.

2.9.1 Management of reactions to combivir

Patients developing adverse reactions that can reasonably be attributed to CBV (e.g. anaemia or neutropenia as reaction to ZDV) may switch the responsible drug to an alternative as in the main part DART trial (e.g. ZDV to d4T). In such a case there would be no need for unblinding and the appropriate CRFs should be completed as in the main part of the trial.

2.9.2 Management of reactions to blinded drug

Patients will be instructed to seek advice from the clinic within 12-24 hours if they develop any of the following symptoms: fever, rash, gastrointestinal symptoms (nausea, vomiting, diarrhoea, or abdominal pain), fatigue, lethargy, malaise, respiratory symptoms (dyspnoea, sore throat, cough), musculoskeletal symptoms (myalgia, myolysis, arthralgia), headache, paraesthesia, oedema, oral lesions, conjunctivitis, blisters, general ill feeling or “flu-like” symptoms, dark urine, tiredness, pale stools, pain, ache, or sensitivity to touch on the right side below the ribs, lack of appetite, yellowing skin or whites of the eyes.

On attending the clinic they should be assessed clinically with the standard symptom checklist and examination and CRFs completed as appropriate. In the CMO arm, results of scheduled laboratory tests can be requested if indicated by symptoms; documented approval for this should be obtained from the Project Leader as in the main DART trial. As with standard management of adverse reactions, if a reaction is felt to be possible but other causes have not been excluded, the patient may be maintained on treatment under close observation. However, if, after investigations and discussion with the Project Leader, the clinical event is considered a potential reaction to ABC or NVP, it is likely that unblinding will be necessary for patient management (after completion of SAE and Hypersensitivity CRFs). This would avoid the possibility of restarting ABC or NVP in a patient who is experiencing a drug-related reaction. It will also facilitate allowing for the long half-life of NVP when stopping drugs.

2.9.3 Management of nevirapine reactions

NVP should be discontinued and not restarted if patients experience hypersensitivity characterized by rash with constitutional symptoms (such as fever, blistering, oral lesions, conjunctivitis, swelling, muscles or joint aches or general malaise) plus visceral involvement such as hepatitis, eosinophilia, granulocytopenia and renal dysfunction.

Patients experiencing mild to moderate rash without constitutional symptoms or visceral involvement during the 14-day lead-in period of 200 mg/day should not have their NVP dose increased until the rash has resolved.

If stopping NVP, if the patient is well enough to tolerate any drugs, CBV should be continued and TDF added as soon as possible. Note that if the reaction is mild it may be possible to start with TDF immediately. If necessary, all drugs should be stopped and CBV/TDF started soon after toxicity symptoms resolve.

2.9.4 Management of abacavir reactions

If, on unblinding, a patient is found to have experienced a reaction to ABC, ABC should be permanently discontinued and not restarted after recovery. If the patient is well enough to take medication, CBV should be continued and TDF added as soon as possible. If necessary, all drugs should be stopped and CBV/TDF started soon after toxicity symptoms resolve.

See also section 2.10 below for further guidelines on the management of ABC reactions.

2.9.5 Safety monitoring

All serious adverse events must be recorded on the Serious Adverse Event Form and reported to the Local Trials Centre and MRC CTU. Grade 3 adverse events should be reported on the next scheduled follow-up unless they lead to treatment modification.

Data on SAEs will be reported, if required, to ethics committees (treatment assignment blinded) and will be tabulated and presented to the Data Safety Monitoring Committee (DSMC) (treatment assignment unblinded).

2.10 Hypersensitivity reaction to abacavir and management of safety - as provided by GSK.

Fatal hypersensitivity reactions have been associated with therapy with ABC. Patients developing signs or symptoms of hypersensitivity (which include fever; skin rash; fatigue; gastrointestinal symptoms such as nausea, vomiting, diarrhoea, or abdominal pain; and respiratory symptoms such as pharyngitis, dyspnoea, or cough) should discontinue ABC as soon as a hypersensitivity reaction is suspected. To avoid a delay in diagnosis and minimize the risk of a life-threatening hypersensitivity reaction, ABC should be permanently discontinued if hypersensitivity cannot be ruled out, even when other diagnoses are possible (e.g., acute onset respiratory diseases, gastroenteritis, or reactions to other medications). There have been infrequent reports of hypersensitivity reaction following reintroduction of ABC, where the interruption was preceded by a single symptom (e.g., rash, fever or gastrointestinal symptoms). On very rare occasions, hypersensitivity reactions have been reported in subjects who have stopped and restarted therapy, and who had no preceding symptoms of a hypersensitivity reaction.

ABC must not be restarted following a hypersensitivity reaction because more severe symptoms will recur within hours and may include life-threatening hypotension and death.

2.10.1 Description of hypersensitivity to Abacavir

In clinical studies, approximately 5% of patients receiving ABC develop a hypersensitivity reaction that in rare cases has proved fatal. This is characterized by the appearance of symptoms indicating multi-organ/body system involvement. The reaction can occur at any time during treatment with ABC, but the symptoms usually appear within the first six weeks of initiation of treatment (median time to onset is 11 days) and most often include fever, rash, gastrointestinal symptoms (nausea, vomiting, diarrhea, or abdominal pain), and lethargy or malaise. Other signs and symptoms may include respiratory symptoms (dyspnoea, sore throat, cough), musculoskeletal symptoms (myalgia, rarely myolysis, arthralgia), headache, paraesthesiae and oedema. Some patients with hypersensitivity reactions were initially thought to have acute onset respiratory diseases, gastroenteritis, or reactions to other medications. This delay in diagnosis of hypersensitivity has resulted in ABC being continued or re-introduced, leading to more severe hypersensitivity reactions or death. Therefore, the diagnosis of hypersensitivity reaction should be carefully considered for patients presenting with symptoms of these diseases. Renal failure, hepatic failure and anaphylaxis have also been reported in association with hypersensitivity reactions.

Physical findings include lymphadenopathy and, occasionally, mucous membrane lesions (conjunctivitis and/or mouth ulceration) and hypotension. The rash is variable and may be absent, but often appears maculopapular or urticarial. Laboratory abnormalities that may accompany ABC hypersensitivity include elevated liver function tests, creatinine phosphokinase, creatinine or lymphopenia.

Symptoms related to this hypersensitivity reaction worsen with continued therapy and usually resolve upon discontinuation of ABC. Restarting ABC following a hypersensitivity reaction results in a prompt return of symptoms within hours. This recurrence of the hypersensitivity reaction may be more severe than on initial presentation and may include life-threatening hypotension and death. Subjects who develop a hypersensitivity reaction must discontinue ABC and must NEVER be re-challenged with ABC (ziagen or trizivir).

2.10.2 Information for patients

Subjects must be informed of the risk of a hypersensitivity reaction to ABC and provided with information to help them recognize the symptoms and signs associated with possible hypersensitivity reactions. Patients must be advised to contact the clinic immediately if they experience such symptoms.

Warning Card - All subjects receiving ABC should receive a wallet-size warning card provided by GlaxoSmithKline (GSK). As each subject is enrolled, the study site must assure that:

a) The subject receives the warning card.(Information will be translated into local languages where necessary)

b) The designated health care provider (e.g., physician, study nurse-coordinator, or pharmacist) reviews the signs and symptoms of hypersensitivity with the subject.

c) The subject verbalizes an understanding of the steps to take in the event of a suspected hypersensitivity, including contacting the study site.

2.10.3 Medical management for possible hypersensitivity to abacavir

If a subject reports symptoms consistent with the diagnosis of ABC hypersensitivity, s/he should be instructed not to take any additional doses of ABC and should be evaluated at the clinic within 12 to 24 hours. All possible, suspected, implied, and/or probable cases of "ABC hypersensitivity" are to be reported as a SAE on the SAE pages of the CRF. ABC hypersensitivity CRF pages (Abacavir Hypersensitivity Reaction Record for Non-GlaxoSmithKline Clinical Trials Only) should also be completed and reported to GSK.

If the clinical presentation cannot be differentiated between hypersensitivity and another medical event (e.g. respiratory infection, gastroenteritis, or reaction to another medication), ABC must be discontinued.

Once the diagnosis of a hypersensitivity reaction has been made, the investigator should take the following steps:

- The patient should permanently discontinue ABC study medication
- Counsel the patient that ABC should never be re-started, as a life-threatening reaction may occur within hours. The patient should never be prescribed any ABC-containing product (e.g. ziagen or trizivir)
- Complete the Hypersensitivity CRF provided by GSK
- Complete the SAE form
- Obtain all hypersensitivity laboratory evaluations including chest x-ray if respiratory symptoms are present.
- The patient should return all unused ABC for disposal to prevent an accidental re-challenge.
- Symptomatic support for the acute reaction may include antihistamines and corticosteroids.
- Symptoms usually start to resolve within 24 hours after stopping therapy. Symptomatic support, such as intravenous fluids for those who develop hypotension, is advised. There are no clinical data demonstrating the benefit of antihistamines or

corticosteroids in the management of hypersensitivity. Nevertheless, symptomatic and/or supportive treatment may be reasonable.

2.10.4 Medical management for Abacavir rash not accompanied by systemic symptoms

The following guidance is provided for clinical management of subjects who experience rash alone in the absence of accompanying diagnosis of ABC hypersensitivity, systemic or allergic symptoms or signs of mucosal or target lesions. The toxicity ratings must be used to appropriately grade cutaneous events when recording AEs.

- ♦ For grade 1 or 2 rash, antihistamines or topical corticosteroids may be prescribed. Use of a Medrol pack is not allowed.
- ♦ Study medications should be continued. The patient's symptoms should be evaluated and followed aggressively for one week or until symptoms resolve or change. If additional symptoms develop, the patient should be re-evaluated in clinic.
- ♦ If the etiology of the rash can be definitively diagnosed as due to concurrent illness or other medical event, routine management should be performed and the documentation of the diagnosis provided.
- ♦ Subjects who develop a grade 3 or 4 rash (e.g., exfoliation, mucosal involvement, or target lesions [erythema multiforme]) or any evidence of Stevens - Johnson syndrome should have all study drugs discontinued and assessed further.
- ♦ Patients should be followed, even though study medications have been discontinued.

2.10.5 Fever and hypersensitivity to Abacavir

The onset of fever may herald hypersensitivity. Subjects reporting fever should be evaluated as above for the possibility of a hypersensitivity reaction to ABC. In the event of a clinical presentation consistent with hypersensitivity, ABC should be discontinued permanently, and the steps taken as stated above.

2.10.6 Special considerations following an interruption of abacavir therapy

If therapy with ABC has been discontinued and restarting therapy is under consideration, the reason for discontinuation should be evaluated to ensure that the subject did not have symptoms of a hypersensitivity reaction. Subjects who have stopped ABC due to possible adverse reactions or illness should be advised to contact their doctor before restarting.

If hypersensitivity reaction cannot be ruled out, ABC should not be restarted.

There have been infrequent reports of hypersensitivity reaction following reintroduction of ABC, where interruption was preceded by a **single symptom**. When subjects who have discontinued ABC present with an indeterminate diagnosis of hypersensitivity (single symptom: rash, fever, malaise/fatigue, gastrointestinal symptoms or a respiratory symptom), the investigator should:

- ♦ Assess the probability that hypersensitivity preceded the interruption. It may be difficult to fully assess a patient's signs and symptoms if there has been a delay of more than a day between the patient stopping therapy and presenting to the physician. If there is doubt about the diagnosis, ABC should not be restarted.
- ♦ Assess the risk:benefit of reinitiating ABC

On very rare occasions hypersensitivity reactions have been reported in subjects who have stopped and restarted ABC therapy and who **had no apparent preceding symptoms** of a hypersensitivity reaction. If a decision is made to restart ABC in this setting, it should be assured that the subject can readily contact the site in the event that symptoms develop. Single missed doses require no special precautions. Subjects should be instructed to resume their normal treatment schedule. If an interruption of 3 or more days is noted, the subject should contact the study investigator. If it is determined that a hypersensitivity reaction is not present, the subject may resume treatment.

2.11 NORA SUBSTUDY FLOW SHEET

Table 8 NORA flow sheet

EVENTS		SUBJECT'S WEEK OF TREATMENT							
	Screening Week -2	Entry Week 0	2	4	8	12	16	20	24
Signed Consent		X							
History & Physical[1]	X	X	X	X	X	X	X	X	X
Symptom check list	X	X	X	X	X	X	X	X	X
Pregnancy Test[2]	X								X
Haematology[3]	X			X*		X*			X*
Biochemistry[4]	X			X*		X*			X*
Lymphocyte Subsets [5]	X					X*			X
Plasma storage[6]	X	X		X		X			X
Adherence Determination[7]				X	X	X	X	X	X

* Not returned to clinician in CMO arm.

1 Clinical: including weight, WHO staging for HIV.

2 See section 7.3.4 if positive.

3 Haematology: Hb, MCV, WBC, Lymphocytes, Neutrophils, Platelets.

4 Biochemistry: Blood Urea Nitrogen or Creatinine, AST or ALT, Bilirubin.

5 CD4, CD8, CD3 percentage and total lymphocyte count.

6 Take 6 ml blood into EDTA. Store DNA pellet (if feasible) See Appendix 7 for storage instructions.

7 See Appendix 8.0.

NB **In addition to the above, patients will return 4-weekly to see the nurse, return used drug containers, and receive 4 weeks of ART**

2.12 DART NORA Substudy Patient Information sheet

(to be given in addition to the DART information sheet for those eligible to participate in the NORA substudy. Patients participating in this substudy should not take part in the STI pilot)

Patient Information sheet for

Nevirapine or Abacavir (NORA) Substudy

You have already been invited to take part in the DART study. If you consent you may be asked if you would like to be enrolled in the nevirapine (NVP) or abacavir (ABC) substudy (NORA). This information sheet gives you details about the substudy. Discuss it with others if you wish. Ask us if anything is not clear or if you would like more information. You will be given a copy of this information sheet to keep.

What is the purpose of NORA?

The purpose of this substudy is to compare the safety of abacavir (ABC) with nevirapine (NVP), both in combination with combivir (ZDV & 3TC) in 600 patients.

What will happen if I wish to take part in the substudy?

You will be required to give separate signed consent to take part. You will first be assigned to one of the two main DART study monitoring arms and then to either combivir with ABC and NVP placebo (inactive drug) or combivir with NVP and ABC placebo. Both allocations will be done by a computer programme to ensure an even balance of patients in each group. You and your doctor will not know which combination of drugs you are going to take (although if your doctor needs to find out s/he can do so). You will take the drugs and placebo every day for 6 months, following the instructions given and after 6 months you will continue taking drugs but without placebo.

How often will I need to attend the clinic?

You will have to come to the clinic 2 and 4 weeks after enrolment. After that you will be followed every 4 weeks. You will be asked if you have any problems and if you have taken all the drugs you were given (you must return any drugs you have not taken). You can ask questions and discuss any problems you have at these visits. Blood tests will be taken after the first 4 weeks and then at 12 and 24 weeks.

What are the disadvantages and risks of taking part?

All anti-HIV drugs can cause side-effects. If you experience what could be a side effect you should tell your doctor at your next clinic visit. Rarely, a reaction or hypersensitivity to ABC may develop. The earliest signs are mild fever, weakness and nausea. A rash may then develop, followed by vomiting, diarrhoea and muscle pain.

If you think you may have this hypersensitivity you must not take any more of your anti-HIV drugs and must come to the next weekday clinic. Your doctor will keep you under close observation. If it is found that your symptoms are due to a particular drug, that drug will be replaced with another anti-

HIV drug. If your doctor thinks you may have had hypersensitivity to abacavir, you will be told NEVER to take abacavir again.

What are the possible benefits of taking part?

The information we get from the substudy will help us to make the best use of these anti-HIV drugs for you and other people with HIV infection.

What happens at the end of the substudy?

The substudy will last for 6 months. After that, if you have improved with the treatment you will continue on your drugs and remain on the monitoring arm you were randomised to before the substudy. If you had any problems with treatment, your doctor would have changed your treatment and you will remain on your new drugs. You will continue to receive drugs and follow-up as in the main DART study.

2.13 DART NORA Substudy Patient consent form

Fill in with Forms 2-4 at Enrolment	DART TRIAL<br>DART NORA CONSENT

Centre: PPD

	Day	Month	Year		
Trial Consent Date			2 0	Clinic/Hospital Number	
Date/Year of Birth			1 9	Age at Screening (if DOB not available)	Initials / Male ○ Female ○

DART Trial Number

DART NORA SUBSTUDY CONSENT

A randomised double-blind phase II substudy trial to evaluate the toxicity of Abacavir compared with Nevirapine in combination with Combivir as first-line therapy in patients participating in the DART trial

NORA

(Nevirapine OR Abacavir)

Please initial (or mark) box if you agree:

1. I have read/been read the information sheet for the NORA substudy and I understand what will be required of me if I participate in the substudy.
   My questions concerning this substudy have been answered by:
   ____
2. I understand that I will be given anti-HIV drugs for 4-5 years while I am in the DART study. After the study, my healthcare will be provided by the national health system.
3. I understand that my participation is voluntary and that I am free to withdraw at any time, without giving a reason and without affecting my normal care and management.
4. I agree to allow blood samples to be taken and stored for testing later. I understand that I may not be given results of tests performed on stored samples.
5. I am willing to allow access to my medical notes to check that the trial is being carried out correctly but understand that strict confidentiality will be maintained.
6. **I agree to take part in the NORA substudy.**

Individual's signature (or thumbprint)	Print name	Date

Witness's signature	Print name	Date

Doctor's signature	Print name	Date

IMPORTANT: **One signed original to be given to patient**
One signed original to be kept on file by the researcher
One signed original to be kept in the clinic notes

CRF Version 1.4, December 2003

Appendix 3.0 Details of Antiretroviral Therapy

Detailed information on all drugs will be provided on regularly updated disk from EMEA website and in individual drug brochures

**Drug (recommended dosing)**	**Class and major toxicities**	**Contraindications**	**Drug interactions**
**Zidovudine (Retrovir) 300mg twice daily (600mg daily)**	NRTI<br>Anaemia, leucopenia, neutropenia, nausea, vomiting	Cannot be used in patients with abnormally low neutrophil counts (< 0. 75 x 10^9/litre) or abnormally low haemoglobin levels (< 7.5 g/dL) *<br>Switch to another drug recommended if patient experiences grade 3 or 4 haematological toxicity.<br>No co-administration with stavudine!	Co-administration with rifampicin decreases AUC of ZDV by 48%: in clinical practice doses are not adjusted.<br>Products inhibiting glucuronidation or directly inhibiting hepatic microsomal metabolism (eg codeine, morphine, indomethacin, naproxen, oxazepam, cimetidine, dapsone) should be administered with caution.
**Lamivudine (Epivir) 150mg twice daily or 300 mg once daily (300 mg daily)**	NRTI<br>Can be administered with or without food<br>Nausea, vomiting , abdominal pain or cramps, headache, arthralgia	In moderate to severe renal impairment with creatinine clearance 30-50 ml/min change to 150mg once daily and if <30ml/min: dose reduction recommended to below 150mg daily using oral solution.<br>Switch to another drug is often practised.	High doses of co-trimoxazole for treatment of PCP should be avoided<br>Co-administration with intravenous ganciclovir or foscarnet is not recommended
**Combivir 300mg zidovudine/ 150mg lamivudine twice daily**	See Retrovir and Epivir	Dosage reduction recommended in reduced renal function (creatinine clearance <50ml/min)<br>In severe hepatic impairment separate Epivir and Retrovir recommended to facilitate Retrovir dose adjustment.<br>Switch to alternative drug is often practised.	See Retrovir and Epivir

* Note that, in DART, patients should not be recruited if neutrophils <0.5x10^9/litre; patients with counts 0.5-0.75x10^9/litre may be included (see section6.2, page 29).

**Drug (recommended dosing)**	**Class and major toxicities**	**Contraindications**	**Drug interactions**
**DDI (Didanosine, Videx)**<br>**200 mg twice daily (or 400mg once daily)**<br>**if <60kg: 125mg twice daily (or 250mg once daily)**	Ingestion with food reduces the amount of didanosine absorbed by approximately 50%.<br>Major toxicities: pancreatitis, peripheral neuropathy, abnormal liver function tests; cases of lactic acidosis (in the absence of hypoxemia), sometimes fatal, usually associated with severe hepatomegaly and hepatic steatosis	Extreme caution in patients with a history of pancreatitis	Medicines which can be affected by stomach acidity (eg. oral azoles such as ketoconazole and itraconazole), should be given at least 2 hours prior to dosing with Videx<br>Co-administration of ddI with medicines that are known to cause peripheral neuropathy or pancreatitis may increase the risk of these toxicities.<br>If treatment with other drugs known to cause pancreatic toxicity is required (e.g. i.v. pentamidine), didanosine should be suspended during such therapy wherever possible<br>Plasma concentrations of some quinolone antibiotics (eg. ciprofloxacin) are decreased by administration with antacids contained in or administered with Videx. Tetracycline antibiotics (e.g.doxycycline) should not be taken with Videx.
**D4T (Stavudine, Zerit)**<br>**40mg twice daily**<br>**if <60kg, use 30mg twice daily**	NRTI<br>Should be taken on empty stomach<br>Headache, chills, fever, abdominal pain, nausea, vomiting, myalgia, arthralgia, malaise, insomnia, depression<br>Peripheral neuropathy, lactic acidosis, pancreatitis, hepatic impairment	In patients with ALT/AST >5 x ULN, D4T should be discontinued<br>Patients with previous history of pancreatitis should be carefully monitored<br>Treatment with D4T should be interrupted in peripheral neuropathy and re-introduced after resolution of symptoms (50% dose level may be considered)<br>No co-administration with ZDV.	

Drug (recommended dosing)	Class and major toxicities	Contraindications	Drug interactions
**Tenofovir (Viread)**<br>**245 mg once daily**<br>**245 mg tablet is equivalent to 300mg of tenofovir disoproxil (as fumarate)**	NRTI<br>Consider interruption of TDF therapy if serum creatinine > 2.0 mg/dl (177umol/l) or serum phosphate < 1.0 mg/dl (0.32 umol/l)<br>Most common side effects: diarrhoea, nausea, vomiting, flatulence (1 % discontinuance rate for GI side effects); grade 1 and 2 serum phosphate decreases requiring phosphate supplementation is some patients	Tenofovir should not be administered to patients with severe renal impairment.<br>No data on patients with mild or moderate renal impairment: caution advised.	Nephrotoxic agents should be avoided but if they have to be administered concomitantly, weekly monitoring of renal toxicity should be considered.<br>No interaction with lamivudine, indinavir or efavirenz<br>In co-administration:<br>AUC for ddI increased by 44%, with lopinavir/ritonavir: 30% increase in Cmax and AUC for tenofovir and about 15% decrease in the same parameters for lopinavir.<br>However, in clinical practice dose adjustments are not made.
**Nevirapine (Viramune)**<br>**200mg once daily for 14 days, followed by 200mg twice daily**<br>**After treatment interruptions lasting >7days, restart with 200mg once daily**	NNRTI<br>Within first 6 weeks:<br>Potential severe and life threatening skin reactions: Stevens Johnsons syndrome and toxic epidermal necrolysis or serious hepatitis/hepatic failure<br>The majority of NVP associated rashes appear within first 6 weeks: dose escalation should NOT be instituted till rash resolves.	Severe hepatic failure<br>NVP should not be re-administered to patients who discontinued this drug for severe rash, rash accompanied by constitutional symptoms, NVP induced hypersensitivity or clinical hepatitis. If AST or ALT > 5xULN, NVP should be stopped	Oral contraceptives should not be used as sole method of contraception as NVP co-administration may decrease the concentration of these agents. Ketoconazole should not be co-administered (replace with fluconazole)<br>Compounds that are enzyme substrates for Cyp3A and CYP2B6 may have altered metabolism when co-administered with NVP and need to be carefully monitored: clarithromycin, cimetidine<br>Rifampicin is Not recommended: rifabutin can be used instead<br>Prednisone DOES NOT decrease NVP related rash.
**Hard gel Saquinavir (Fortovase) (boosted)**<br>**1600mg plus 100mg ritonavir once daily**	Protease Inhibitor<br>Major toxicities:<br>Hyperglycaemia and fat redistribution	Do not administer in severe renal or hepatic impairment	Should not be co-administered with rifabutin or rifampicin .<br>Due to inhibition of CYP3A4, saquinavirshould not be administered with compounds that are substrates for this enzyme; e.g. terfenadine, , midazolam, pimozide, ergot derivatives

Drug (recommended dosing)	Class and major toxicities	Contraindications	Drug interactions
**Lopinavir/ Ritonavir (Kaletra) 133mg/33.3mg capsules, three capsules twice daily**	Protease Inhibitor<br>Major toxicities: Hyperglycaemia and fat redistribution<br>Pancreatitis has been reported in patients with hyperglycaemia	Do not administer in severe hepatic impairment. Caution with severe renal impairment	No co-administration with rifampicin!<br>Due to inhibition of CYP3A4, Kaletra should not be administered with compounds that are substrates for this enzyme; e.g. terfenadine, midazolam, pimozide, ergot derivatives<br>Caution required when used with products known to induce GT internal prolongation e.g. clarithramycin, erythromycin<br>Oral contraception effectiveness may be decreased.
**Lopinavir/ Ritonavir (Aluvia) 200mg/50mg tablets, 2 tablets twice daily**	Protease Inhibitor<br>Major toxicities: Hyperglycaemia and fat redistribution<br>Pancreatitis has been reported in patients with hyperglycaemia	Do not administer in severe hepatic impairment. Caution with severe renal impairment	No co-administration with rifampicin!<br>Due to inhibition of CYP3A4, Aluvia should not be administered with compounds that are substrates for this enzyme; e.g. terfenadine, midazolam, pimozide, ergot derivatives<br>Caution required when used with products known to induce GT internal prolongation e.g. clarithramycin, erythromycin<br>Oral contraception effectiveness may be decreased.
**Indinavir (Crixivan) 800mg three times daily**	Protease Inhibitor<br>Major toxicities: nephrolithiasis (adequate hydration of patients taking the drugs is recommended);<br>Fat redistribution and hyperglycaemia	Do not administer in severe renal or hepatic impairment	No co-administration with rifampicin!<br>Dose increase of rifabutin and dose reduction of indinavir are recommended when two compounds are co-administered.<br>Due to inhibition of CYP3A4, Indinavir should not be administered with compounds that are substrates for this enzyme; e.g. terfenadine, midazolam, pimozide, ergot derivatives

Appendix 4.0 Toxicity Grading and Management

4.1 Table of Common Toxicity Criteria

Note: ULN denotes upper limit of local reference range ("upper limit of normal")

	Grade 1	Grade 2	Grade 3	Grade 4
**HAEMATOLOGICAL**				
Haemoglobin	8.0-9.4 g/dl	7.0-7.9 g/dl	6.5-7.0 g/dl	<6.5 g/dl
Leucopenia	3.0-3.9 $x10^3$ cells/μl	2.0-2.9 $x10^3$ cells/μl	1.9-1.0 $x10^3$ cells/μl	<1.0 $x10^3$ cells/μl
Neutrophils	1.00-1.50 $x10^3$ cells/μl	0.75-0.99 $x10^3$ cells/μl	0.50-0.74 $x10^3$ cells/μl	<0.50 $x10^3$ cells/μl
Platelets	75-99 $x10^3$ cells/μl	50-74 $x10^3$ cells/μl	20-49 $x10^3$ cells/μl	<20 $x10^3$ cells/μl
Prothrombin time	>1.0-1.25 x ULN	>1.25-1.5 x ULN	>1.5-3.0 x ULN	>3.0 x ULN
Partial prothrombin time	>1.0-1.66 x ULN	>1.66-2.33 x ULN	>2.33-3.0 x ULN	>3.0 x ULN
Methaemoglobin	5-10.0 %	10.1-15.0 %	15.1-20.0 %	>20 %
**BIOCHEMISTRY**				
Hyponatraemia	130-135 mmol/l	123-129 mmol/l	116-122 mmol/l	<116 mmol/l
Hypernatraemia	146-150 mmol/l	151-157 mmol/l	158-165 mmol/l	>165 mmol/l
Hypokalaemia	3.0-3.4 mmol/l	2.5-2.9 mmol/l	2.0-2.4 mmol/l	<2.0 mmol/l
Hyperkalaemia	5.6-6.0 mmol/l	6.1-6.5 mmol/l	6.6-7.0 mmol/l	>7.0 mmol/l
Hypocalcaemia (corrected for albumin)	1.95-2.10 mmol/l<br>7.8-8.4 mg/dl	1.75-1.94 mmol/l<br>7.0-7.7 mg/dl	1.53-1.74 mmol/l<br>6.1-6.9 mg/dl	<1.53 mmol/l<br><6.1 mg/dl
Hypercalcaemia (corrected for albumin)	2.70-2.93 mmol/l<br>10.8-11.7 mg/dl	2.94-3.19 mmol/l<br>11.8-12.7 mg/dl	3.20-3.44 mmol/l<br>12.8-13.7 mg/dl	>3.44 mmol/l<br>>13.7 mg/dl
Hypomagnesaemia	0.47-0.59 mmol/l<br>1.2-1.4 mg/dl	0.35-0.46 mmol/l<br>0.9-1.1 mg/dl	0.25-0.34 mmol/l<br>0.6-0.8 mg/dl	<0.25 mmol/l<br><0.6 mg/dl
Hypophosphataemia	0.58-0.72 mmol/l<br>2.0-2.4 mg/dl	0.44-0.57 mmol/l<br>1.5-1.9 mg/dl	0.30-0.43 mmol/l<br>1.0-1.4 mg/dl	<0.3 mmol/l<br><1.0 mg/dl
Hypoglycaemia	3.1-3.6 mmol/l<br>55-64 mg/dl	2.2-3.0 mmol/l<br>40-54 mg/dl	1.7-2.1 mmol/l<br>30-39 mg/dl	<1.7 mmol/l<br><30 mg/dl
Hyperglycaemia (fasting)	6.5-9.0 mmol/l<br>118-164 mg/dl	9.1-14.0 mmol/l<br>165-255 mg/dl	14.1-28.0 mmol/l<br>256-509 mg/dl	>28.0 mmol/l<br>>509 mg/dl<br>or ketoacidosis
Triglycerides (fasting)	1.8-2.2 mmol/l<br>150-190 mg/dl	2.3-5.6 mmol/l<br>191-470 mg/dl	5.7-10.0 mmol/l<br>471-830 mg/dl	>10.0 mmol/l<br>>830 mg/dl
Triglycerides (non-fasting)	-	4.8-8.9 mmol/l<br>400-750 mg/dl	9.0-14.4 mmol/l<br>751-1200 mg/dl	>14.4 mmol/l<br>>1200 mg/dl
Albumin	26-30 g/l<br>2.6-3.0 g/dl	20-25 g/l<br>2.0-2.5 g/dl	<20 g/l<br><2.0 g/dl	-
Bilirubin	>1.0-1.5 x ULN	>1.5-2.5 x ULN	>2.5- 5.0 x ULN	>5.0 x ULN
AST or ALT or GGT	1.25-2.5 x ULN	>2.5-5.0 x ULN	>5.0-10.0 x ULN	>10.0 x ULN
Alkaline phosphatase	1.25-2.5 x ULN	>2.5-5.0 x ULN	>5.0-10.0 x ULN	>10.0 x ULN
Amylase (total or pancreatic or salivary)	>1.0-1.5 x ULN	>1.5-2.5 x ULN	>2.5-5.0 x ULN	>5.0 x ULN
Creatinine	>1.0-1.5 x ULN	>1.5-3.0 x ULN	>3.0-6.0 x ULN	>6.0 x ULN
Creatinine kinase	>1.0-2.0 x ULN	>2.0-4.0 x ULN	>4.0-6.0 x ULN	>6.0 x ULN
Lactate	>1.0- 2.0 x ULN<br>without acidosis	≥2 x ULN<br>without acidos<br>is	>1.0 XULN<br>with acidosis , without life threatening consequenses	>1.0 XULN<br>with acidosis and life threatening consequenses
Urea	1.25-2.5 x ULN	>2.5-5.0 x ULN	>5.0-10.0 x ULN	>10.0 x ULN
**URINALYSIS**				

	Grade 1	Grade 2	Grade 3	Grade 4
Proteinuria	1+ or<br><0.3% or<br><3g/l or<br>200mg-1g loss/day	2-3+ or<br>0.3-1.0% or<br>3-10g/l or<br>1-2g loss/day	4+ or<br>>1.0% or<br>>10g/l or<br>2-3.5g loss/day	nephrotic syndrome or >3.5 g loss/day
Haematuria	microscopic only	gross, no clots	gross and clots	obstruction or requiring transfusion
**GASTROINTESTINAL**				
Stomatitis/mouth ulcers	mild discomfort, no limits on activity	some limits on eating or talking	eating/ talking very limited	requiring IV fluids
Nausea	mild or transient discomfort, maintains reasonable intake	moderate discomfort or significantly decreased intake for <3 days	severe discomfort or minimal intake for ≥3 days	hospitalisation required
Vomiting	mild or transient; 2-3 episodes per day or mild vomiting lasting <1 week	moderate or persistent; 4-5 episodes per day or vomiting lasting ≥1 week	severe vomiting of all food/fluids in 24 hours or orthostatic hypotension or IV fluids required	hypotensive shock or hospitalisation required for IV fluids
Diarrhoea	mild or transient; 3-4 loose stools/day or mild diarrhoea lasting <1 week	moderate or persistent; 5-7 loose stools/day or diarrhoea lasting ≥1 week or nocturnal loose stools	bloody diarrhoea or orthostatic hypotension or >7 loose stools/day or requiring IV fluids	hypotensive shock or hospitalisation required for IV fluids
Clinical pancreatitis	mild abdominal pain, amylase <2.5x ULN, other causes excluded	moderate abdominal pain, amylase <2.5x ULN, other causes excluded	severe abdominal pain, amylase >2.5x ULN, hospitalisation required	severe abdominal pain, shock/ hypovolaemia, amylase>5x ULN, hospitalisation required
**NEUROLOGICAL**				
Headache	mild, no treatment	moderate or requires non-narcotic analgesia	severe or responds to first narcotic	intractable or requiring repeated narcotics
Consciousness	difficulty in concentration or memory	mild confusion or lethargy <50% waking hours	disoriented or stupor >50% waking hours	coma or seizures
Mood	mild anxiety or depression	treatment required for anxiety or depression	treatment and assistance required, severe depression, mania or anxiety	acute psychosis or hospitalisation
Psychosis	mild agitation or confusion	some limitation in activities of daily living and minimal treatment required	treatment and assistance required, severe agitation or confusion	toxic psychosis or hospitalisation
Cerebellar	slight incoordination or dysdiadochokinesia	intention tremor or dysmetria or slurred speech or nystagmus	ataxia requiring assistance to walk or arm incoordination interfering with activities of daily living	unable to stand
Motor	mild weakness in muscle of feet but able to walk or mild increase or decrease in reflexes	moderate weakness in feet (unable to walk on heels or toes), mild weakness in hands but still able to do most hand tasks, or loss of previously present reflex or development of hyperreflexia or unable to do deep knee bends due to weakness	marked distal weakness (unable to dorsiflex toes or foot drop) and moderate proximal weakness (eg in hands interfering with activities of daily living or requiring assistance to walk or unable to rise from chair unassisted)	confined to bed or wheelchair because of muscle weakness
Clinical myopathy	minimal findings	moderate myalgia or difficulty climbing stairs or rising from sitting position, able to walk, may need NSAID	moderate to severe myalgia needing NSAID, assistance required for walking or general activities	severe myalgia unrelated to exercise requiring narcotics, unable to walk or necrosis or oedema

	Grade 1	Grade 2	Grade 3	Grade 4
Sensory	mild impairment (decreased sensation eg vibratory, pinprick, hot/cold in great toes) in focal area or symmetric distribution	moderate impairment (moderately decreased sensation eg vibratory, pinprick, hot/cold to ankles) or joint position or mild impairment that is not symmetrical	severe impairment (decrease or loss of sensation to knees or wrists) or loss of sensation of moderate degree in multiple different body areas (eg upper and lower extremities)	sensory loss involves limbs and trunk
Parathaesia (burning, tingling etc)	mild discomfort, no treatment	moderate discomfort, requiring non-narcotic analgesia	severe discomfort or symptoms respond to narcotic analgesia	incapacitating or not responsive to narcotics
Peripheral neuropathy	mild paraesthesia, numbness, pain or weakness, not treated	moderate paraesthesia, numbness or pain, objective weakness, requires analgesic	severe, narcotic required, interferes with normal activity	intolerable, incapacitating, unable to walk despite narcotics, paralysis
**RESPIRATORY**				
Bronchospasm	transient, no treatment, 70-80% peak flow or FEV1	requires treatment, normalises with bronchodilator, 50-<70% peak flow or FEV1	no normalisation with bronchodilator, 25-<50% peak flow or FEV1, retractions	cyanosis, intubated or <25% peak flow or FEV1
**CARDIOVASCULAR**				
Cardiac arrhythmia	-	asymptomatic, transient dysrhythmia, no treatment	recurrent or persistent dysrhythmia, symptomatic treatment required	unstable dysrhythmia, hospitalisation and treatment required
Hypertension	transient, increase >20mm/Hg, no treatment	recurrent, chronic increase >20mm/Hg, requires treatment	acute treatment required, outpatient hospitalisation possible	hospitalisation required
Hypotension	transient, orthostatic hypotension no treatment	symptoms correctable with oral fluid treatment	IV fluid required, no hospitalisation required	hospitalisation required
Pericarditis	minimal effusion	mild/moderate asymptomatic effusion, no treatment	symptomatic effusion, pain, ECG changes	tamponade or pericardiocentesis or surgery required
Haemorrhage	microscopic or occult	mild, no transfusion	gross blood loss or transfused 1-2 units	massive blood loss or transfused >2 units
Cardiomyopathy	Asymptomatic, therapy not indicated	Asymptomatic, therapy indicated	Symptomatic CHF responsive to intervention	Refractory CHF, poorly controlled; intervention such as ventricular assist device, or heart transplant indicated
**OTHER**				
Fever, oral, >12 hours	37.7-38.5^{0}C	38.6-39.5^{0}C	39.6-40.5^{0}C	>40.5^{0}C
Fatigue	normal activity reduced by <25%	25-50% decrease in normal activity	>50% decrease in activity, cannot work	unable to care for self
Hypersensitivity	pruritus without rash	localised urticaria	generalised urticaria or angioedema	anaphylaxis
Rash	erythema or pruritus	diffuse maculopapular rash or dry desquamation	vesiculation or moist desquamation or ulceration	exfoliative dermatitis or mucous membrane involvement or suspected Stevens-Johnson or erythema multiforme or necrosis requiring surgery
Lipodystrophy	Detectable by study participant	Detectable on physical exam by healthcare provider	Disfiguring OR Obvious on casual visual inspection	
General	mild, transient, easily tolerated, no treatment	moderate, discomfort, interrupts usual activity, may require minor treatment	severe, considerable interference with usual activity, requires treatment or medical intervention	incapacitating or life-threatening, requires treatment and/or hospitalisation

4.2 Table of Clinical Signs, Symptoms, Monitoring and Management of Symptoms of Serious Adverse Effects of Antiretroviral Drugs that Require Drug Discontinuation (adapted from WHO guidelines ref 10 Annex 11B)

Adverse Effect	Possible Offending Drug(s)	Clinical Signs / Symptoms	Management
Acute hepatitis	Nevirapine (NVP); more uncommon with zidovudine (ZDV), didanosine (ddI), stavudine (d4T) (<1%); and protease inhibitors (PI), most frequently with ritonavir (RTV)	Jaundice, liver enlargement, gastrointestinal symptoms, fatigue, anorexia; NVP-associated hepatitis may have hypersensitivity component (drug rash, systemic symptoms, eosinophilia)	Monitor serum transaminases, bilirubin. All ARV should be stopped until symptoms resolve. NVP may need to be permanently discontinued.
Acute pancreatitis	ddI, d4T	Nausea, vomiting, and abdominal pain	If possible, monitor serum pancreatic amylase, lipase. All ART should be stopped until symptoms resolve. Restart ART with change to different NRTI, preferably one without pancreatic toxicity (e.g., ZDV)
Lactic acidosis	All nucleoside analogue reverse transcriptase inhibitors (NRTIs)	Initial symptoms are variable: a clinical prodromal syndrome may include generalized fatigue and weakness, gastrointestinal symptoms (nausea, vomiting, diarrhea, abdominal pain, hepatomegaly, anorexia, and/or sudden unexplained weight loss), respiratory symptoms (tachypnea and dyspnea) or neurologic symptoms (including motor weakness).	Discontinue all ARV; symptoms may continue or worsen after discontinuation of ART. Supportive therapy. Regimens that can be considered for restarting ART include a PI combined with an NNRTI and tenofovir
Hyper-sensitivity reaction	Abacavir (ABC) Nevirapine (NVP)	ABC: Constellation of acute onset of symptoms including: fever, fatigue, myalgia, nausea/vomiting, diarrhea, abdominal pain, pharyngitis, cough, dyspnea (with or without rash). While these symptoms overlap those of common infectious illness, the combination of acute onset of both respiratory and gastrointestinal symptoms after starting ABC is more typical of a hypersensitivity reaction. NVP: Systemic symptoms of fever, myalgia, arthralgia, hepatitis, eosinophilia with or without rash.	Discontinue all ARVs until symptoms resolve. The reaction progressively worsens with drug administration and can be fatal. Administer supportive therapy. Do not rechallenge with ABC (or NVP), as anaphylactic reactions and death have been reported. Once symptoms resolve, restart ARVs with change to different NRTI if ABC-associated or to PI- or NRTI-based regimen if NVP-associated.
Severe rash / Stevens-Johnson syndrome	Non nucleoside reverse transcriptase inhibitors (NNRTIs): nevirapine (NVP)	Rash usually occurs during the first 2-4 weeks of treatment. The rash is usually erythematous, maculopapular, confluent, most prominent on the body and arms, may be pruritic and can occur with or without fever. Life-threatening Stevens-Johnson Syndrome or toxic epidermal necrolysis (SJS/TEN) has been reported in ~0.3% of infected individuals receiving NVP	Discontinue all ARVs until symptoms resolve. Permanently discontinue NVP for rash with systemic symptoms such as fever, severe rash with mucosal lesions or urticaria, or SJS/TEN; once resolves, switch ART regimen to different ARV class (e.g., 2 NRTIs and tenofovir or 2 NRTIs and PI).

Adverse Effect	Possible Offending Drug(s)	Clinical Signs / Symptoms	Management
Severe peripheral neuropathy	ddI, d4T, (3TC – unusual)	Pain, tingling, numbness of hands or feet; distal sensory loss, mild muscle weakness, and areflexia can occur.	Stop suspect NRTI and switch to different NRTI that does not have neurotoxicity (e.g., ZDV, ABC). Symptoms usually resolve in 2-3 weeks.
Severe anaemia	ZDV	Severe pallor, tachycardia at rest Shortness of breath on exertion (SOBOE)	Stop ZDV and switch to another drug

4.3 Guide to management of toxicities

4.3.1 Grade 1 (clinical or laboratory (LCM arm only)):

- Continue study drugs

4.3.2 Grade 2 (clinical or laboratory (LCM arm only)):

- Continue study drugs
- If relevant, monitor more closely, and for LCM arm only, consider more frequent laboratory assessments.
- Work-up to exclude other causes

4.3.3 Grade 3 (clinical or laboratory (LCM arm only)):

- Repeat clinical observation within 72 hours for confirmation.
- LCM arm: if relevant, obtain repeat confirmatory laboratory results within 72 hours
- CMO arm: if clinically indicated, laboratory results can be requested
- Work-up to exclude other causes
- Clinician has the option of immediately stopping the study drugs if a confirmatory laboratory test cannot be performed within 72 hours, or if the clinician determines that the continuation of study drugs is unsafe while awaiting test results

4.3.4 Grade 4(clinical or laboratory):*

- If relevant, obtain confirmatory laboratory results within 72 hours and fill in adverse event form
- Work-up to exclude other causes
- Clinician has the option of immediately stopping the study drugs if a confirmatory laboratory test cannot be performed within 72 hours, or if the clinician determines that the continuation of study drugs is unsafe while awaiting test results
- For all confirmed Grade 4 toxicities that can be clearly attributable to one or more antiretroviral drug(s), consider stopping relevant drug(s) and switching to alternative drug(s) as indicated in section 8.1.4, page 40. If therapy needs to be stopped, stop all drugs until toxicity resolves to ≤Grade 2. Then, restart therapy but discontinue the implicated drug(s) permanently and replace as indicated in section 8.1.4, page 40. If any doubt about management, discuss with the principal trial investigator

Appendix 5.0 Clinical progression and WHO Definitions

WHO clinical staging for HIV/AIDS

**Clinical Stage 1:**<br>Asymptomatic<br>Persistent generalised lymphadenopathy (PGL)<br>Performance scale 1: asymptomatic, normal activity
**Clinical Stage 2:**<br>Weight loss, <10% of body weight<br>Minor mucocutaneous manifestations (seborrheic dermatitis, prurigo, fungal nail infections, recurrent oral ulcerations, angular cheilitis)<br>Herpes Zoster, within the last 5 years<br>Recurrent upper respiratory tract infections (e.g. bacterial sinusitis)<br>And/or performance scale 2: symptomatic, normal activity.
**Clinical stage 3:**<br>Weight loss, >10% of body weight<br>Unexplained chronic diarrhoea, >1 month<br>Unexplained prolonged fever (intermittent or constant), > 1 month<br>Oral candidiasis (thrush)<br>Oral hairy leukoplakia<br>Pulmonary tuberculosis, within the past year.<br>Severe bacterial infections (e.g. pneumonia, pyomyositis)<br>And/or Performance scale 3: bed-ridden, <50% of the day during the last month
**Clinical stage 4:**<br>HIV wasting syndrome, as defined by CDC[1]<br>Pneumocystis carinii pneumonia<br>Toxoplasmosis of the brain<br>Cryptosporidiosis with diarrhoea, >1 month<br>Cryptococcosis, extra pulmonary<br>Cytomegalovirus (CMV) disease of an organ other than liver, spleen or lymph nodes<br>Herpes Simplex Virus (HSV) infection, mucocutaneous >1 month, or visceral any duration<br>Progressive multifocal leukoencephalopathy (PML)<br>Any disseminated endemic mycosis (e.g. histoplasmosis, coccidioidomycosis)<br>Candidiasis of the oesophagus, trachea, bronchi or lungs<br>Atypical mycobacteriosis, disseminated<br>Non-typhoid Salmonella septicaemia<br>Extra Pulmonary tuberculosis<br>Lymphoma<br>Kaposi's sarcoma (KS)<br>HIV encephalopathy, as defined by CDC[2]<br>And/or Performance scale 4: bed-ridden, >50% of the day during the last month

(note: Both definitive and presumptive diagnoses are acceptable)

[1] HIV wasting syndrome: weight loss of >10% of body weight, plus either unexplained chronic diarrhoea (>1 month), or chronic weakness and unexplained prolonged fever (>1 month).

[2] HIV encephalopathy: clinical finding of disabling cognitive and/or motor dysfunction interfering with activities of daily living, progressing over weeks to months, in the absence of a concurrent illness or condition other than HIV infection that could explain the findings.

Appendix 6.0 Patient Information sheet and Consent Forms (Templates)

6.1 Patient information sheet for DART study

DART

Development of AntiRetroviral Therapy in Africa for adults infected with HIV.

Patient information sheet

You are being invited to take part in a research study. Please read this information carefully or have someone read it to you. You will be given a copy of this form to keep. Discuss it with others if you wish. Ask us if you would like more information. Take time to decide whether or not you wish to take part. If you are already registered with a current health system provider (e.g. TASO in Uganda), joining this work will not affect your rights to any other benefits it offers.

If you decide not to take part now or to withdraw from the study later, this will not influence the care you receive now (e.g. if you are a TASO member you will still get all your benefits from TASO).

What is the reason for doing the study?

HIV infection cannot be cured, but taking anti-HIV (antiretroviral) drugs can control it. We want to find out how best to use anti-HIV drugs so that the treatment is safe and effective. At present doctors use regular blood tests to see how the drugs are working and to check for side-effects. We want to find out whether the drugs can be taken without doing many blood tests. We also want to find out if the drugs need to be taken all the time or can they safely be taken with breaks (3 months on drugs, 3 months off).

How can I join the study?

After reading this information sheet you will be asked to give consent to be screened, which will involve being seen by the doctor and giving a blood sample. After two weeks you will be told whether you are eligible for the study and asked to sign a DART study consent form if you choose to participate.

What will happen if I take part?

You will be started on 3 anti-HIV drugs and you will be assigned to one of two groups:

- **clinical monitoring-only group**

  or

- **clinical and laboratory monitoring group**

In the **clinical monitoring-only group** your doctor will make decisions about your treatment based only on your symptoms. The results of your blood tests will be monitored independently and your doctor will be informed if there is a problem.

In the **clinical and laboratory-monitoring group** your doctor will make decisions about your treatment based on your symptoms and the results of your blood tests.

You will be asked to attend clinics 2 and 4 weeks after entry to the study, then every 4 weeks to see the nurse and every 3 months to see the doctor and to provide blood. At each visit you will be given a supply of anti-HIV drugs and asked to follow exact instructions on how to take these drugs.

It is very important that you do not miss any doses and do not share your medication with others.

If anti-HIV drugs are not taken properly, they will lose their effect, as the virus becomes resistant. Other medicines should only be taken on the advice of your doctor. You will be counselled to practice safe sex, as anti-HIV drugs cannot prevent transmission of HIV infection to sexual partners. If you do not attend your clinic visit, we would like to send a field-worker to your home to find out how you are.

After you have been taking anti-HIV drugs for 6 months, if your immune system has improved (CD4 count greater than 200cells/mm^3) you will be assigned into one of two groups (by computer):

- interrupted treatment (3 months on and 3 months off treatment)

  or

- continuous treatment

If your immune system has not improved enough at 6 months, you will be examined again at 12 months. If your CD4 count then is greater than 200 cells/mm^3, you will be eligible to be assigned to either interrupted or continuous treatment.

Treatment interruption pilot study

The first 100 patients who interrupt treatment will have blood tests and visits every month. Independent experts will look at the results on these 100 patients and if they find that treatment interruption is safe, all other patients with good response to treatment at 6 or 12 months will be assigned to either interrupted or continuous treatment. The pilot study will last for 6 months. After that, if you have no problems with interrupted treatment you will continue to interrupt drugs. If you had any problems with interrupted treatment, your doctor may suggest you take drugs continuously. After the pilot, you will continue on the monitoring arm you were assigned before the pilot. You will continue to receive drugs and follow–up as in the main DART study.

Blood samples

Blood tests may be done whenever you see the doctor. This may cause some discomfort and/or bruising in some patients. About two tablespoons of blood will be taken at one time. Some blood may be stored for later testing and you may not be given these results.

What drugs will be used in the DART study?

1. combivir (zidovudine + lamivudine in one pill)
2. tenofovir
3. nevirapine
4. didanosine
5. stavudine
6. saquinavir with ritonavir or other similar drugs from the same group

When you start the trial you will be given combivir with a third drug. The third drug will be tenofovir in Uganda and tenofovir or nevirapine in Zimbabwe. We will supply drugs for the duration of the study (4-5 years). After the study, your care will be provided by the national health system (or TASO in Uganda) including provision of anti-HIV drugs in line with current government plans.

Side effects

Anti-HIV drugs, like all drugs, have side effects, which are sometimes serious (less than 5%). If you experience symptoms tell your clinic doctor at your next visit, or if you are worried you should come to the clinic as soon as possible. It may be necessary to stop the drug(s) after which the problem usually goes away. We can replace drugs causing problems with other drugs.

Effects of drug interruption

During drug interruption it is possible that your HIV disease may get worse. If you get symptoms between visits you should come to the clinic. Your doctor may restart your treatment early (before the end of 3 months) and if problems continue when you are back on treatment, your drugs may be changed.

Pregnancy

HIV treatment given early in pregnancy may harm the unborn child. Pregnant and breastfeeding women will not be entered into this trial. A pregnancy test will be carried out at screening and at regular intervals during the study and women will be encouraged to avoid becoming pregnant during the trial. If a woman does becomes pregnant while in the trial, treatment will be continued without interruptions.

Confidentiality

Information about you will be kept confidential and will not be made available to anyone who is not connected with the study without your consent.

If you would like more information about this work please ask the doctors, nurses or counsellors. If you still need more information, call:

Insert names and telephone numbers as appropriate for each trial site

Name: ……………………………………………………………..

Telephone Number: ……………………………………………….

6.2 Consent for Screening for the DART Trial

fill in with Forms 0 & 1	DART TRIAL<br>DART SCREENING CONSENT

Centre: PPD

	Day	Month	Year		
Screening Consent Date			2 0 0 _	Clinic/ Hospital Number	
Date/Year of Birth			1 9 _ _	Age (if DOB not available)	Initials ___ Male ❍ Female ❍

DART TRIAL SCREENING CONSENT

I have read the information sheet for the DART trial and would like to be screened to see whether I am eligible for this trial.

I understand this will involve being seen by the doctor, who will ask questions about my health and my household, and having some blood taken.

Individual's signature (or thumbprint)	Print name	Date

Witness's signature	Print name	Date

Doctor's signature	Print name	Date

6.3 Patient consent form for DART Trial

fill with Forms 2-4 at Enrolment	DART TRIAL<br>**DART TRIAL CONSENT**

Centre: PPD

	Day	Month	Year			
Trial Consent Date			2 0 0	Clinic/ Hospital Number		
Date/Year of Birth			1 9	Age at Screening (if DOB not available)	Initials	Male ❍ Female ❍

DART TRIAL CONSENT

A randomised trial of monitoring practice and structured treatment interruptions in the management of antiretroviral therapy in adults with HIV infection in Africa

DART

(Development of Anti-Retroviral Therapy in Africa)

Please initial box if you agree:

1. I have read/been read the information sheet for the DART study and I understand what will be required of me if I participate in the study.

   My questions concerning this study have been answered by:

   ____

2. I understand that I will be given anti-HIV drugs for 4-5 years while I am in the DART study. After the study, my healthcare will be provided by the national health system.
3. I understand that I may withdraw from the study at any time, without giving a reason and without affecting my normal care and management.
4. I agree to allow blood samples to be taken and stored for testing later. I may not be given results of tests performed on stored samples.
5. I am willing to allow access to my medical notes to check that the trial is being carried out correctly but understand that strict confidentiality will be maintained.
6. **I agree to take part in the DART study.**
7. I also agree to take part in the treatment interruption pilot study if asked to do so.

Individual's signature (or thumbprint)	Print name	Date

Witness's signature	Print name	Date

Doctor's signature	Print name	Date

IMPORTANT: **One signed original to be given to patient**
One signed original to be kept on file by the researcher
One signed original to be kept in the clinic notes

6.4 Patient information sheet for the second line randomisation feasibility study

DART

Development of AntiRetroviral Therapy in Africa

2nd Line Feasibility study

Patient information sheet

You are already taking part in DART but are being invited to additionally join an extra part of the study. This is because the anti-HIV drugs you have been taking are no longer working as well and need to be changed to different ones. Please read this information carefully or have someone read it to you. You will be given a copy of this form to keep. Discuss it with others if you wish. Ask us if you would like more information. Take time to decide whether or not you wish to take part.

If you decide not to take part in the DART 2nd line feasibility study, this will not affect the continuing care and treatment you receive now in the DART study.

What is the reason for doing the study?

With your recent clinical history it is now clear that the anti-HIV medicines that you are taking are no longer working as well as they have. It is now necessary to change to a new combination of anti-HIV/AIDS medicines. We call this second-line treatment.

We will provide the new second-line medicines within the current DART study and everything else will continue as before, except for the change of medication. You will continue to receive the same care and have the same access to the trial staff and clinic. We will continue to offer exactly the same level of services and support.

Much of the power in second-line treatment comes from Aluvia (also called Kaletra). Drugs like these are called “protease inhibitors” (PIs). They are made more powerful by adding small amounts of another drug (Ritonavir) and are then called “boosted” PIs. Although there are several different PIs available and Aluvia is currently the recommended boosted PI in Uganda and Zimbabwe.

We normally use anti-HIV drugs in combination to work together to attack the virus in different ways. Aluvia will therefore be supported by other drugs, at least for the first 24 weeks. Which other drugs you need will depend on what you have already been using up to now in your initial or first-line treatment in DART.

There are several different ways of using the new powerful second-line medicines; and it is not clear which is the best.

- ♦ One question is exactly which combination of other drugs best supports Aluvia. The options have different strengths and possible side-effects and may not be as effective when used in second-line as in first-line. The best balance between these factors is not known in second-line treatment anywhere yet.

This second-line feasibility study is therefore evaluating different combinations of the drugs to support Aluvia.

In DART, we continue to try to work out the best ways of treating HIV in Africa which is why we want to evaluate these different second line approaches - to see if all are the same or find out if some ways are better than others. The DART team emphasise that it is completely unclear to experts whether all approaches are ultimately the same for you; or whether one second line approach is in fact better for you than another.

What are the possible risks & benefits of different drug combinations to support Aluvia?

NOTE THAT FOR CLARITY DIFFERENT VERSIONS OF THE SHEET WILL BE PRODUCED WITH ONLY ONE OF THESE SECTIONS ON EACH. THE PARTICIPANT WILL BE GIVEN THE VERSION RELEVANT TO THEIR PRVIOUS TREATMENT HISTORY.

NNRTI FAILURES

You have already taken at least 2 drugs from the class called "nucleosides" (NRTIs, Combivir – actually Zidovudine and Lamivudine), together with a "non-nucleoside" (NNRTI, Nevirapine). Your HIV will have at least some resistance to these NRTI drugs and it is likely that the NNRTIs would not work at all. Therefore most of the power from your second-line drugs will come from Aluvia.

There are 2 options for support drugs we will use in DART are:

1. To use 2 new NRTI drugs that you have not taken before (Abacavir and Didanosine), even though we believe that these drugs are not likely to be fully effective because of resistance the virus has developed to the NRTIs you have taken before.
2. To exploit the idea that in becoming resistant to the drugs you have been taking the virus has probably also become weaker (less "fit"). By continuing to take Combivir we hope to keep the virus weaker. As you have already been taking Combivir this should give no new side-effects. You will also take one new NRTI (Tenofovir), which should have some activity against the virus.

NRTI FAILURES

You have only taken drugs from one class of HIV drugs – the "nucleoside" (or "nucleotides", NRTI), so we expect drugs from the other 2 classes to be as active as if you had not already taken any HIV drugs. So you will be given a boosted PI (Aluvia) and a "non-nucleoside" (NNRTI, Nevirapine or Efavirenz). It is not clear if added a third drug, another NRTI, will add any extra benefit or may increase side-effects.

The 3 options we will use in DART are:

1. To just take a boosted PI and an NNRTI
2. To take a boosted PI, an NNRTI and lamivudine. You have already taken lamivudine and your HIV will be resistant to it but is likely to also be weaker (less "fit"). By continuing to take lamivudine we hope to keep this weakness
3. To take a boosted PI, an NNRTI and Didanosine. Didanosine is an NRTI that you have not already taken, so may have some beneficial activity

How can I join the study?

After reading this information sheet you will be asked to give consent and to sign a DART 2nd Line feasibility study consent form if you choose to participate.

What will happen if I take part?

You will be allocated by chance to receive:

1. one of two or three different ART combination regimens to support Aluvia (or another PI).

All participants in the second-line study of DART trial will continue to be followed up in the DART clinic and receive exactly the same standards of care, provided free of charge.

An independent group of international experts will also continue regularly to monitor the progress of all patients in the second line feasibility study and should it be apparent that one approach or trial arm is better or worse than another then that arm or part of the trial will be stopped and all patients move to the better treatment option.

What will happen if I do not to take part?

If you do not wish to join the second line study in DART that is fine - we will still provide drugs for your second-line therapy and you will continue in current DART follow-up for the duration of the first part of DART. Declining to participate in the evaluation of second-line treatment in DART does not in any way or form affect your abilities or rights to continue to be part of the DART trial.

Whether or not you choose to take part in this study, after you have received 24 weeks of Aluvia based second-line therapy, you will be approached about co-enrolment into another study looking at the maintenance phase of second-line treatment.

If you have already switched to a second line regimen

If you have already switched to a second line regimen in DART you will not be able to join this feasibility study.

Blood samples

Blood tests may be done whenever you see the doctor, you will see the doctor as often as when you started in DART. This may cause some discomfort and/or bruising in some patients. About two tablespoons of blood will be taken at one time. Some blood may be stored for later testing and you may not be given these results. There will be no difference in the amount of blood taken if you join the DART 2nd Line Feasibility study or just continue in the main DART trial.

Side effects

Anti-HIV drugs, like all drugs, have side effects, which are sometimes serious (less than 5%). If you experience symptoms tell your clinic doctor at your next visit, or if you are worried you should come to the clinic as soon as possible. It may be necessary to stop the drug(s) after which the problem usually goes away. We can replace drugs causing problems with other drugs.

Pregnancy

Some HIV drugs (but not all) given early in pregnancy may harm the unborn child. A pregnancy test will be carried out at regular intervals during DART. If you think you might become pregnant please tell your doctors so that they can work out which drugs you should take. If a woman does become pregnant while in the trial, treatment will be continued.

Will joining this feasibility study have any effect on my treatment after the DART trial ends?

At the end of DART, all participants will be transferred into national programmes. Your government has promised that you will continue to be treated.

Confidentiality

Information about you will be kept confidential and will not be made available to anyone who is not connected with the study without your consent.

We have tried to explain clearly the different approaches being evaluated. If you feel that you still do not fully understand the different issues then it is fine to pause and ask for further information now. You also do not need to decide now but can come back later.

If you would like more information about this work please ask the doctors, nurses or counsellors. If you still need more information, call:

Insert names and telephone numbers as appropriate for each trial site

Name: ………………………………………………………………..

Telephone Number: ……………………………………………….

6.5 Patient consent form for the feasibility second line randomisation

DART TRIAL

DART 2ND LINE FEASIBILITY STUDY CONSENT

Centre: PPD

	Day	Month	Year		
Consent Date			2 0 0	Clinic/Hospital Number	
Date/Year of Birth			1 9	Age at Screening (if DOB not available)	Initials: ___ Male ❍ Female ❍

DART Trial Number

DART 2nd LINE FEASIBILITY STUDY CONSENT

A randomised trial of monitoring practice and structured treatment interruptions in the management of antiretroviral therapy in adults with HIV infection in Africa

DART

(Development of Anti-Retroviral Therapy in Africa)

Please initial (or mark) box if you agree:

1. I have read/been read the information sheet for the 2nd Line Feasibility Study of the DART Trial and I understand what will be required of me if I participate in the study.

   My questions concerning this study have been answered by:

   ____

2. I understand that I may withdraw from the 2nd Line Feasibility Study at any time and that if I still stay in DART I will be offered treatment with whichever combination of DART drugs my doctor decides.

3. I understand that I will continue to be given anti-HIV drugs in the DART study. After the study, my healthcare will be provided by the national health system.

4. **I agree to take part in the DART 2nd Line Feasibility Study.**

Individual's signature (or thumbprint)	Print name	Date

Independent witness's signature (only required if patient is unable to read)	Print name	Date

Doctor's signature	Print name	Date

IMPORTANT: **One signed original to be given to patient**
One signed original to be kept on file by the researcher
One signed original to be kept in the clinic notes

Appendix 7.0 Handling and Storage of Specimens

7.1 Bloods

7.1.1 Processing of EDTA blood for Plasma and cell store

For best results, centrifuge within 2 hours of drawing blood. If the time to processing is not within the same working day, **do not proceed**.

1. Centrifuge at 1500g for 15 minutes to separate cells from plasma.

2. Using a sterile pipette, collect the plasma more than 5mm above the buffy cell coat layer (grey layer above red cell layer).

3. Place the plasma, using up to three aliquots, in the cryovials provided for the trial. A minimum of two aliquots is required Routinely three cryovials of plasma and one cryovial containing the DNA pellet (if feasible) should be stored at each visit.

4. Label the cryovials with the patient "PLASMA" identification labels

5. Store cell pellet for later DNA isolation (if feasible). After plasma has been removed transfer the cell pellet into cryovial

6. Label the cryovials with the patient "CELLS" identification label and store at $^{-}80$°C (if not possible then $^{-}20$°C)

7.1.2 Storage of samples

1. Store within 4- 6 hours of drawing blood at -70°C (temperatures to -50°C allowed if this is not available). Place upright in the cryobox provided for the trial.

2. Add the Patient ID label to the Specimen Storage Log for sample. Complete the details for type of specimen processed, visit month, total number of tubes and storage temperature. Start a new log sheet when either the sheet is full or when starting to fill a new cryobox.

Appendix 8.0 Adherence protocol

FORM 14	DART TRIAL<br>**ADHERENCE**

Centre: PPD

	Day	Month	Year		
Date Form Completed	☐☐	☐☐	2 0 0 ☐	Clinic/ Hospital Number	☐☐☐☐☐☐☐☐
Date/Year of Birth	☐☐	☐☐	1 9 ☐☐	Age at Screening (if DOB not available) ☐☐	Initials ☐☐☐ Male ❍ Female ❍

DART Trial Number ☐☐☐☐☐☐ **CMO ☐ LCM ☐**

Please tell us if you have been able to take your anti-HIV treatment as planned.
You do not have to answer all the questions if you do not want to.
A NURSE SHOULD HELP YOU COMPLETE THIS FORM

1. **Are you late for this scheduled visit?** (tick box) Yes ☐ No ☐ If yes, specify number of days late ☐☐

2. **During the past 4 days, on how many days have you missed taking all (or part) of your HIV drug doses?**
   1 day ☐ 2 days ☐ 3 days ☐ 4 days ☐ No days ☐

3. **Indicate in the box below when you took your drugs.** Write the names of anti-HIV drugs in the first column. Put an 'X' if you took your drugs and put '0' if you missed a dose on each day.

Name of HIV drug	Did you take your drug? Yesterday		2 days ago		3 days ago		4 days ago	
	am	pm	am	am	am	pm	am	pm
	am	pm	am	pm	am	pm	am	pm
	am	pm	am	pm	am	pm	am	pm
	am	pm	am	pm	am	pm	am	pm

4. **Some people find that they forget to take their pills on the weekends.**
   Did you miss any of your anti-HIV drugs last weekend (Saturday or Sunday)? (tick box)......... Yes ☐ No ☐

5. **Do any of your anti-HIV drugs have special instructions, such as "take with food", or "on an empty stomach"?** (tick box) .... Yes ☐ No ☐
   If yes, how often did you follow these special instructions over the last 4 days? (tick box)
   Never ☐ Some of the time ☐ About half of the time ☐ Most of the time ☐ All of the time ☐

6. **Which one (if any) of your anti-HIV drugs is the easiest to take?** (write in here): ____________________________

7. **Which one (if any) of your anti-HIV drugs is the most difficult to take?** (write in here):________________________

8. **When was the last time you missed any of your anti-HIV drugs?** (tick box):
   Within the last week ☐ 1- 2 weeks ago ☐ 2- 4 weeks ago ☐ 1- 3 months ago ☐
   More than 3 months ago ☐ Never ☐

9. **(Complete at scheduled doctor visit only) People may miss taking their anti-HIV drugs for many reasons. Here is a list of possible reasons.** If you never miss your drugs do not answer this question

In the past month how often have you missed taking your anti-HIV drugs because you:	Never	Rarely	Sometimes	Often
a) Were away from home?	☐	☐	☐	☐
b) Were too busy with other things?	☐	☐	☐	☐
c) Simply forgot?	☐	☐	☐	☐
d) Had too many pills to take?	☐	☐	☐	☐
e) Wanted to avoid side-effects?	☐	☐	☐	☐
f) Did not want other people to notice you taking pills?	☐	☐	☐	☐
g) Had a change in daily routine?	☐	☐	☐	☐
h) Felt like the drug was toxic or harmful?	☐	☐	☐	☐
i) Were asleep through the dose time?	☐	☐	☐	☐
j) Felt sick or ill?	☐	☐	☐	☐
k) Felt depressed or overwhelmed?	☐	☐	☐	☐
l) Had a problem taking pills at a specified time (with meals or on an empty stomach)?	☐	☐	☐	☐
m) Ran out of pills?	☐	☐	☐	☐
n) Felt good?	☐	☐	☐	☐

Appendix 9.0 Post DART Trial issues

The DART trial, as with all clinical trials, is funded for a specific time-limited period by the funding bodies involved. Drugs will be provided for the duration of the trial for up to 6 years for each patient.

It may be expected that as all patients in the trial will be receiving HAART, many patients will be alive at the end of the trial, and still be benefiting from ART. For them cessation of therapy would not be appropriate. The various study sites in Uganda and Zimbabwe have made this dilemma clear in their ethical submissions. The DART team has initiated collaboration with the governments in Uganda and Zimbabwe and external sponsors to address these concerns and ensure that DART trial is entirely ethically sound and acceptable to the countries. Both Zimbabwe and Uganda have applied to the Global Fund and access to ARV has expanded substantially. It is expected that post-trial healthcare for the patients will be obtained within the framework of this partnership. There will be a phased transition of DART participants to other programmes over a period of up to 6 months covering quarter 4 of 2008 and quarter 1 of 2009.

This partnership with local health authorities also includes collaborations with:

- The Ugandan Ministry of Health Task Force set up to deliver more drug treatment for Ugandan citizens and to establish the mechanisms of drug provision by the Government.
- The [redacted] which has recently been established in [redacted] has set up a large treatment centre for ART.
- The large and medium sized businesses to seek to derive support from the companies for long-term ART after the trial of their employees.
- Medical insurance companies who are taking an active interest in the issues around ART and are considering ways to provide ART for their clients.
